# Berry curvature multipoles in Kagome superconductor CsV_3_Sb_5_

**DOI:** 10.1038/s41467-026-77618-6

**Published:** 2026-09-10

**Authors:** Shanshan Liu, Prasun Boyal, Enze Zhang, Qifeng Hu, Xu Han, Kian Ping Loh

**Affiliations:** 1 Department of Chemistry, National University of Singapore, Singapore, Singapore; 2 National Laboratory of Solid State Microstructures, Collaborative Innovation Center of Advanced Microstructures, School of Physics, Nanjing University, Nanjing, People’s Republic of China

**Keywords:** Superconducting properties and materials, Superconducting devices, Electronic properties and materials, Phase transitions and critical phenomena

## Abstract

Electronic nematic transitions in correlated systems often involve substantial reorganization of Berry curvature. Nonlinear transport measurements serve as a powerful tool for distinguishing between different types of Berry curvature multipoles that characterize nematic phases and topological changes. Herein, we systematically measured nonlinear responses up to the sixth harmonic in the Kagome superconductor CsV_3_Sb_5_. Our results identify the odd-order nonlinear Hall effect as the dominant signature of nematicity. During the superconducting transition, vortex dynamics induce rapid spatial variations in the phase gradient *∇*ϕ, which significantly amplify the Berry curvature quadrupole (BCQ) relative to Berry curvature dipole (BCD). The third-order nonlinear response peaks sharply near the superconducting transition, where enhanced vortex–antivortex fluctuations and nonlinear vortex dynamics amplify the BCQ-related nonlinear Hall response. First-principles calculations further support this interpretation by revealing pronounced Berry-curvature quadrupoles induced by chiral loop-current order near the Fermi level. This work establishes BCQ as a sensitive probe of electronic nematicity, particularly when enhanced by superconducting vortex dynamics.

## Abstract (web summary)

Higher-order nonlinear Hall responses can reveal Berry-curvature multipoles associated with electronic nematicity. Here, the authors identify a vortex-enhanced third-order Hall response in CsV3Sb5 and uncover a Berry-curvature quadrupole associated with chiral loop-current order

## Introduction

In the study of topological materials, the quantum geometric tensor ${{{\mathcal{Q}}}}_{{ij}}$ provides a comprehensive description of the Bloch states. This tensor is composed of a real part, the quantum metric ${g}_{{ij}}$, and an imaginary part, the Berry curvature ${\varOmega }_{{ij}}$. While the Berry curvature monopole and dipole have been extensively studied in relation to the intrinsic anomalous Hall effect^1,2^ and second-order nonlinear Hall effects (NLHE)^3–6^, recent advancements in condensed matter physics have identified higher-order moments, specifically the BCQ, as the primary drivers of third-order nonlinear transport^7–10^ in systems where lower-order responses are suppressed by symmetry. Different nonlinear orders encode distinct aspects of the momentum-space Berry curvature distribution, implying that higher-order responses can remain allowed or even become dominant when lower-order terms are suppressed by symmetry. More generally, high-order nonlinear Hall responses provide access to finer geometric information of electronic states beyond the dipolar level, thereby enriching the framework of nonlinear transport^7–10^. Related high-order transverse nonlinear responses have also been reported in thermoelectric transport, such as the fourth-order nonlinear anomalous Nernst effect^11^, underscoring the growing relevance of high-order Berry-curvature-related phenomena beyond the linear regime.

The Kagome superconductor CsV_3_Sb_5_ has emerged as a paradigmatic platform for exploring the interplay between topology, strong electronic correlations, and unconventional electronic orders^12–23^. Owing to its vanadium Kagome lattice, CsV_3_Sb_5_ hosts Dirac dispersions, flat bands, and van Hove singularities near the Fermi level, strongly amplifying interaction effects and predisposing the system to multiple competing instabilities^18,21–23^. Experimentally, CsV_3_Sb_5_ undergoes a 2×2 charge density wave (CDW) transition, followed by the onset of superconductivity at lower temperatures^20,24–28^. Importantly, a growing body of experimental evidence indicates that the superconducting (SC) state is unconventional in nature, characterized by strong-coupling behavior, nodal or anisotropic gaps, multiband participation, and an intimate entanglement with the pre-existing CDW order^15,18,21,29,30^. Beyond superconductivity, transport and spectroscopic measurements have revealed anomalous Hall effect^25,31,32^, electronic chirality^27,28^, nematic responses^26,33^, and pronounced history dependence deep inside the CDW phase^34,35^, pointing to spontaneous time-reversal symmetry breaking without conventional magnetic order. Orbital loop current order has been proposed as a possible microscopic origin of these observations, although the precise symmetry properties of the low-temperature CDW state remain under active debate^36,37^. In this context, CsV_3_Sb_5_ provides a particularly compelling setting for nonlinear Hall studies—while the nonlinear Hall effect is generally understood in terms of Berry curvature dipole in inversion-symmetry-breaking systems, the coexistence of strong correlations, unconventional superconductivity, and emergent time-reversal symmetry breaking in CsV_3_Sb_5_ raises the prospect of highly nontrivial Berry-curvature distributions and enhanced nonlinear Hall responses beyond simple band-structure expectations. Elucidating the origin and symmetry constraints of NLHE in this correlated Kagome superconductor is therefore essential for understanding how orbital physics and collective electronic order manifest in nonlinear transport.

In this work, we recorded large in-plane nonlinear transport in the Kagome superconductor CsV_3_Sb_5_ across multiple nonlinear orders (from second-order up to sixth-order). Pronounced peak features are observed in the nonlinear Hall signal in the superconducting state, with the peak current appearing close to the superconducting critical current and disappearing above the superconducting transition temperature, which are suggestive of asymmetric vortex scattering. The extracted second- and third-order nonlinear Hall susceptibility reach values of 1.1 × 10^−6 ^m/V and 4.4 × 10^−9^ m^2^/V^2^, respectively, and drop dramatically when the temperature above is SC transition temperature. First-principles calculations further identify chiral loop-current-induced Berry-curvature quadrupoles as the dominant mechanism of the third-order Hall response, establishing a direct connection between time-reversal-symmetry breaking and higher-order nonlinear transport. Broadly, this work advances the framework of nonlinear transport by demonstrating access to multipole moments like quadrupoles and beyond using third-order non-linear conductivity measurement, which is highly sensitive to electronic nematic phase transitions.

## Results and discussion

### High-order nonlinear response in the superconducting CsV_3_Sb_5_

**Fig. 1:**
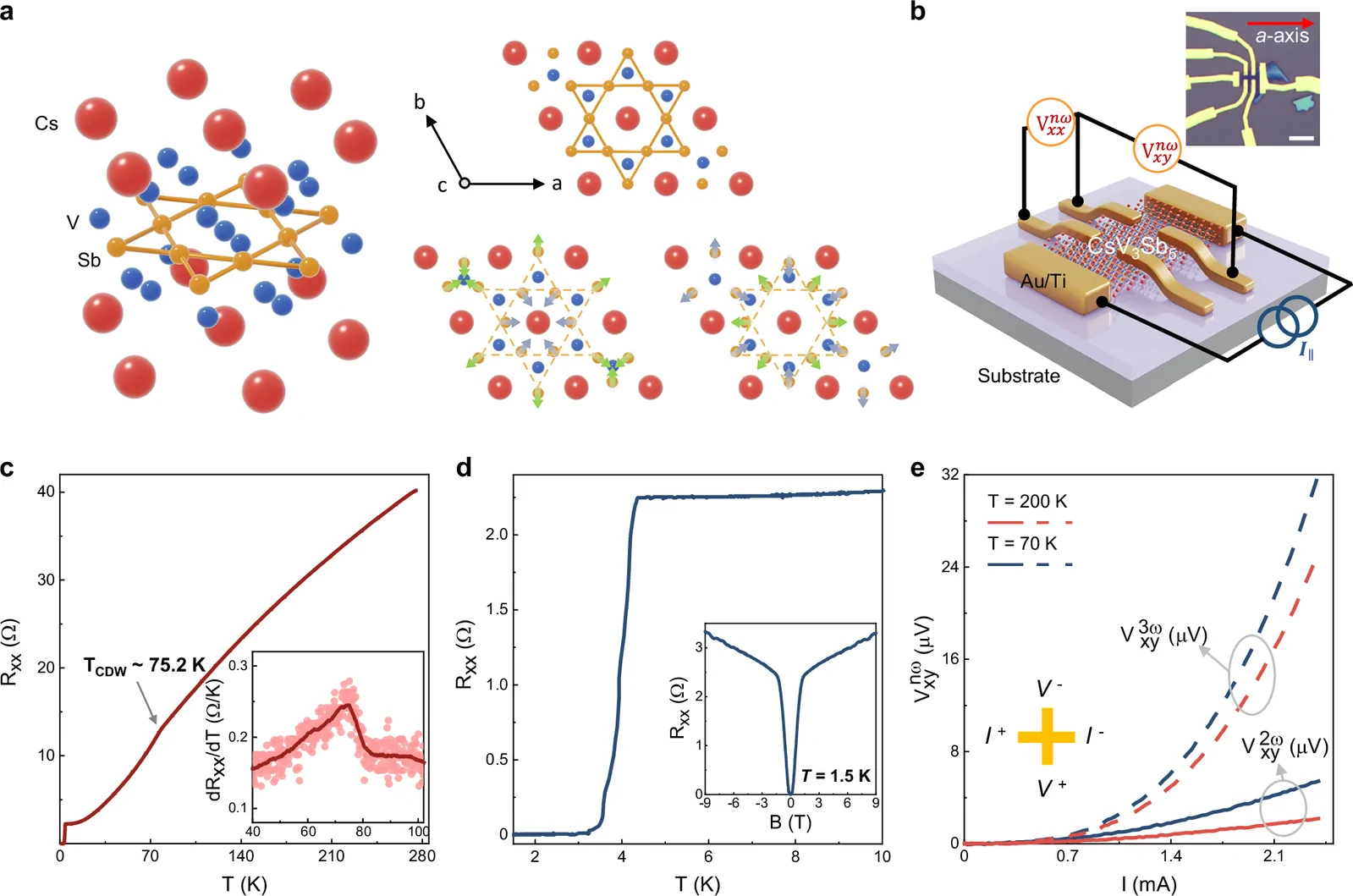
Crystal structure and basic transport properties of CsV_3_Sb_5_. **a** Crystal structure of CsV_3_Sb_5_, crystallizing in the hexagonal space group *P6/mmm* with an undistorted vanadium Kagome lattice at high temperature, and the in-plane vanadium atoms undergo small distortion displacements (as indicated by the green and blue arrows in the right panel) forming breathing Kagome patterns upon cooling below the CDW transition temperature. **b** Schematic geometry of a Hall-bar device. Top inset, optical image of a Hall-bar device. Scale bar: 5 μm. **c** Temperature dependence of the longitudinal resistance ${R}_{{xx}}$, showing a kink at the charge-density-wave transition temperature ${T}_{{{\rm{CDW}}}}\approx 75.2$ K. The inset displays the corresponding peak in ${dR}_{{xx}}/{dT}$. The circles represent the experimental data, and the solid line is a smoothed curve serving as a guide to the eye. **d** Low-temperature ${R}_{{xx}}(T)$ curve highlighting the onset of superconductivity at ~4.2 K and zero resistance below ~3 K. Inset, magneto-transport data measured at 1.5 K, where Shubnikov–de Haas oscillations emerge at high magnetic fields, and superconducting features are suppressed above ~1.3 T. **e** The second- and third-harmonic transverse voltages measured above and below the CDW transition, at 200 K and 70 K, respectively.

CsV_3_Sb_5_ crystallizes in a hexagonal structure with space group P6/mmm at high temperature, featuring an undistorted vanadium Kagome lattice (Fig. 1a). A cascade of electronic phase transitions giving rise to nematic phases unfolds as the system is cooled. Well above the superconducting transition at ~2.5 K, the system enters a charge-ordered phase characterized by star-of-David (SoD) or Tri-Hexagonal (TrH) structure that breaks the translational symmetry of the Kagome lattice^36,37^. Upon further cooling toward superconducting transition temperature, a second electronic instability occurs. This transition is marked by a pronounced breaking of the inherent six-fold rotational (C_6_) symmetry of the Kagome lattice^38^. This symmetry-broken state, which persists into the superconducting dome, manifests as the emergence of a unidirectional 4a_0_ charge order and highly anisotropic quasiparticle scattering^14,39^. Such CDW-induced reconstruction strongly modifies the Fermi surface and its associated Berry geometry. Importantly, a finite Berry curvature dipole (BCD) requires not only broken inversion symmetry but also the removal of rotational symmetries, such as an out-of-plane C_2_ rotation, that would otherwise force the in-plane BCD to vanish. The further symmetry lowering associated with the nematic/chiral CDW state can therefore permit a finite, direction-selective BCD, which may contribute to the observed one-fold second-order nonlinear Hall response. At the same time, the strongly reconstructed and anisotropic Fermi surface can generate higher-order Berry-curvature moments, including a Berry curvature quadrupole (BCQ), providing a possible geometric basis for the two-fold third-order nonlinear Hall response.

To test its transport signals, we exfoliated and fabricated standard Hall bar CsV_3_Sb_5_ devices (Fig. 1b). Upon cooling, the CDW transition first appears at ${T}_{{CDW}}\approx 75.2$ K, with a small kink appearing in the longitudinal resistance ${R}_{{xx}}$ versus temperature curve, as displayed in Fig. 1c. This transition is more clearly identified by a pronounced peak in ${{dR}}_{{xx}}/{dT}$, as displayed in the inset of Fig. 1c. With further cooling, ${R}_{{xx}}$ exhibits a sharp drop at the onset of superconductivity ~4.2 K, and becomes fully superconducting at ~3 K (Fig. 1d). The inset of Fig. 1d presents the raw magneto-transport data measured at 1.5 K, where clear Shubnikov-de Haas (SdH) oscillations are resolved at high magnetic field ( > 5 Tesla), which is superimposed with superconducting features that will be fully suppressed at field larger than 1.3 Tesla.

**Fig. 2:**
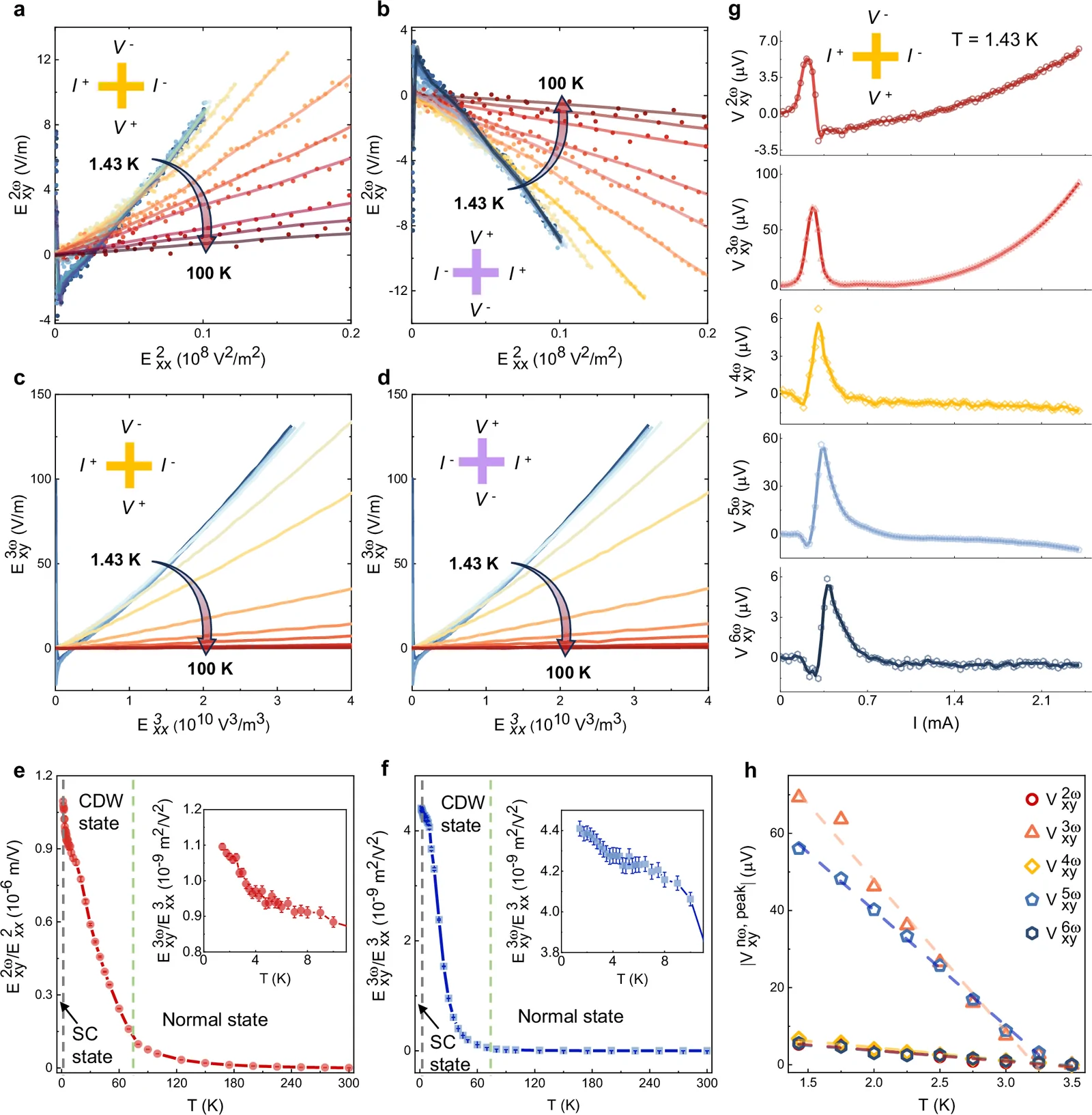
Higher harmonics nonlinear Hall response in the superconducting CsV_3_Sb_5_. Linear dependence of (**a**, **b**) ${E}_{{xy}}^{2\omega }$ on ${E}_{{xx}}^{2}$ and **c**, **d** ${E}_{{xy}}^{3\omega }$ on ${E}_{{xx}}^{3}$ ranging from 1.43 K to 100 K measured at two opposite measurement configurations with reversed current direction and voltage probe electrodes simultaneously. Yellow and purple crosses show opposite measurement setups. **e**, **f** Temperature dependence of the corresponding nonlinear susceptibilities $\frac{{E}_{{xy}}^{2\omega }}{{E}_{{xx}}^{2}}$ and $\frac{{E}_{{xy}}^{3\omega }}{{E}_{{xx}}^{3}}$, which decrease monotonically with increasing temperature and drop sharply above the CDW transition. The error bars reflect the standard fitting error of ${E}_{{xy}}^{2\omega }$ versus ${E}_{{xx}}^{2}$ (${E}_{{xy}}^{3\omega }$ versus ${E}_{{xx}}^{3}$). **g** Multiple-order harmonic transverse voltage as a function of alternating current measured at 1.43 K. In the superconducting state, pronounced peaks emerged in the second- to sixth-harmonic transverse voltages (${V}_{{xy}}^{2\omega }$, ${V}_{{xy}}^{3\omega }$, ${V}_{{xy}}^{4\omega }$, ${V}_{{xy}}^{5\omega }$, and ${V}_{{xy}}^{6\omega }$) near the superconducting transition. **h** Peak magnitude of multiple-order harmonic transverse voltage as a function of temperature. The peak feature decreases with temperature increasing, and well above superconducting transition temperature (${T}_{c}$), the peak features vanish.

Next, we studied nonlinear transport in the superconducting state and normal state. Here, ac current *I* was applied along the crystallographic a-axis at a fixed frequency (*ω* = 13.77 Hz), and the different-harmonic voltage response (2*ω* = 27.54 Hz, 3*ω* = 41.31 Hz, 4*ω* = 55.08 Hz, 5*ω* = 68.85 Hz, 6*ω* = 82.62 Hz) were recorded in the transverse directions (inset of Fig. 2a, Supplementary Note 3). The transition into the CDW state at approximately 75.2 K lowers the symmetry of the pristine Kagome lattice and produces a reconstructed 2×2 (i.e. [2a_0_]) superlattice. Such reconstruction substantially modifies the electronic structure and can generate pronounced Berry-curvature textures and their higher-order momentum-space moments. Although CsV_3_Sb_5_ is nominally non-magnetic, μSR and polar Kerr measurements have provided evidence for time-reversal-symmetry breaking within the CDW regime^35,40–42^. This hidden magnetic order has been proposed to originate from orbital loop currents associated with a chiral or complex CDW rather than conventional spin magnetism. Importantly, the precise onset of this time-reversal-symmetry-breaking component need not coincide exactly with the primary CDW transition and may develop further within the reconstructed CDW state. As shown in Fig. 1e, the nonlinear transverse voltages are substantially enhanced at 70 K, deep within the CDW phase, compared with the normal state at 200 K. The pronounced enhancement upon entering the CDW regime therefore indicates that CDW-induced Fermi-surface reconstruction and symmetry lowering strongly amplify the nonlinear transverse response, potentially assisted by the development of loop-current-induced Berry curvature, as further demonstrated in Fig. 2e–f.

To systematically examine the nonlinear response over the full temperature range, we convert the measured harmonic voltages into electric fields through ${E}_{{xy}}^{n\omega }={V}_{{xy}}^{n\omega }/{L}_{y}$ and ${E}_{{xx}}={V}_{{xx}}/{L}_{x}$, where ${E}_{{xy}}^{n\omega }$ and *E*_*xx*_ denote the *n*-order harmonic transverse and first-harmonic longitudinal electric fields, respectively. Here, ${L}_{y}$ and ${L}_{x}$ are the transverse and longitudinal dimensions of the Hall bar. As displayed in Fig. 2a, c, ${E}_{{xy}}^{2\omega }$ and ${E}_{{xy}}^{3\omega }$ scale linearly with the square of ${E}_{{xx}}$ (${E}_{{xx}}^{2}$) and cube of ${E}_{{xx}}$ (${E}_{{xx}}^{3}$), respectively, confirming the quadratic and cubic current dependence under zero magnetic field. ${E}_{{xy}}^{2\omega }$ changes its sign while ${E}_{{xy}}^{3\omega }$ preserves the same sign changes when simultaneously reversing the current direction and voltage probe electrodes (Fig. 2b, d), consistent with the expected symmetry properties of even- and odd-order nonlinear transverse responses. *E*_*xx*_ below the superconducting temperature is calculated by using the resistance at *T* = 4 K (normal state). The corresponding nonlinear Hall susceptibility, defined as ${E}_{{xy}}^{2\omega }/{E}_{{xx}}^{2}$ and ${E}_{{xy}}^{3\omega }/{E}_{{xx}}^{3}$ are summarized in Fig. 2e, f to characterize the temperature dependence of the nonlinear response. Both susceptibility terms decrease monotonically with increasing temperature and drop sharply above ${T}_{{CDW}}$, indicating that the nonlinear Hall response is intimately tied to the CDW phase. The ${E}_{{xy}}^{2\omega }/{E}_{{xx}}^{2}$ and ${E}_{{xy}}^{3\omega }/{E}_{{xx}}^{3}$ reach 1.1 × 10^−6 ^m/V and 4.4 × 10^−9^ m^2^/V^2^ at low temperatures, respectively, demonstrating pronounced nonlinear responses in CsV_3_Sb_5_. In particular, the second-order susceptibility reaches the µm V⁻¹ scale, placing the nonlinear Hall response among the large values reported in quantum materials. The substantial third-order susceptibility further indicates that higher-order nonlinear transport becomes strongly enhanced in the low-temperature symmetry-broken state.

Upon further cooling into the superconducting regime, additional nonlinear features emerge. As shown in Supplementary Fig. 1a, with increasing current the superconducting zero-resistance state is gradually suppressed and a finite-resistance state develops, as represented by ${V}_{{xy}}$ (top panel). Around this transition, a pronounced peak develops in the second-, third-, fourth-, fifth-, and sixth-harmonic transverse voltages (${V}_{{xy}}^{2\omega }$, ${V}_{{xy}}^{3\omega }$, ${V}_{{xy}}^{4\omega }$, ${V}_{{xy}}^{5\omega }$, and ${V}_{{xy}}^{6\omega }$) signals, as displayed in Fig. 2g. In contrast, the peak behavior vanishes when the temperature is above superconducting transition, as illustrated in Supplementary Fig. 1. The second-, third-, fourth-, and fifth-transverse voltages exhibit clear quadratic, cubic, quartic, and quintic dependence on the applied current under zero magnetic field, as verified by the linear relation between ${E}_{{xy}}^{n\omega }/{E}_{{xx}}^{n}$ (Fig. 2a, c, Supplementary Figs. 2-3). Although the sixth-harmonic response is clearly observable, its relatively small amplitude results in larger fitting uncertainty, therefore, we limit the quantitative power-law analysis to the second- through fifth-harmonic signals. Moreover, ${V}_{{xy}}^{2\omega }$, ${V}_{{xy}}^{4\omega }$, and ${V}_{{xy}}^{6\omega }$ reverse sign upon simultaneous reversal of the current direction and voltage probe configuration, while ${V}_{{xy}}^{3\omega }$ and ${V}_{{xy}}^{5\omega }$ retain their sign (Supplementary Fig. 1), consistent with the opposite parity expected for even- and odd-order nonlinear transverse responses under reversal of measurement configuration. Additional data of the observed overlapped multiple-order signal under varying ac frequencies, as shown in Supplementary Fig. 4, further clarifies the origin and excludes the thermal effect. The temperature evolution of the peak magnitude is summarized in Fig. 2h. The peak feature decreases continuously with increasing temperature and disappears above the superconducting transition temperature (${T}_{c}$), indicating that superconductivity further enhances the nonlinear response on top of the CDW-induced background. The peak position and overall signal evolution are largely insensitive to variations in lock-in amplifier settings and measurement conditions (Supplementary Note 4 and Supplementary Figs. 7-8), supporting the intrinsic origin of the observed peak behavior.

**Fig. 3:**
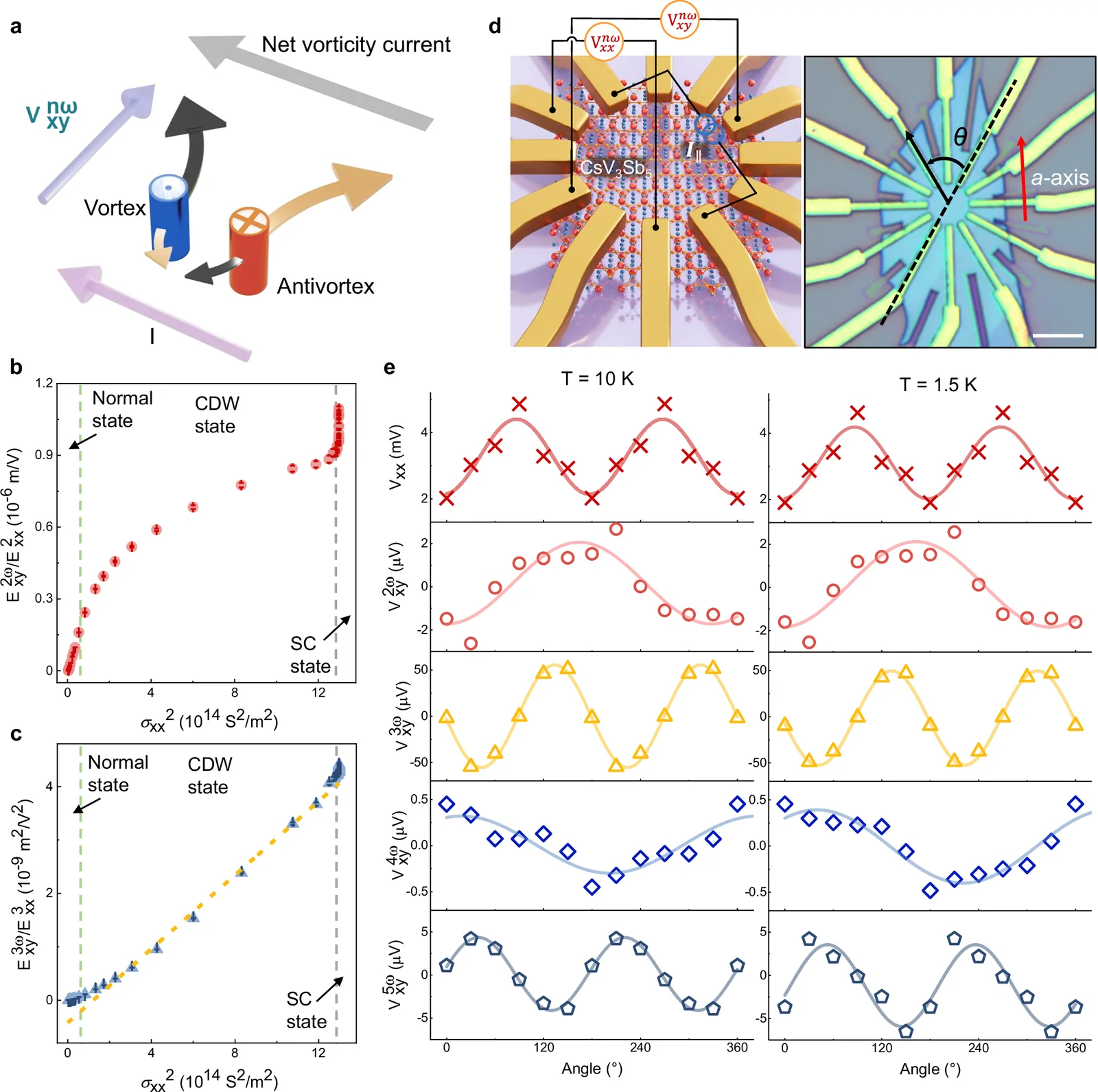
Physical origin of nonlinear transport. **a** Schematic of the vortex-dominated nonlinear transport, where asymmetric vortex and antivortex trajectories give rise to an antiparallel vorticity current and a transverse second-harmonic voltage. **b**, **c** Nonlinear susceptibility $\frac{{E}_{{xy}}^{2\omega }}{{E}_{{xx}}^{2}}$ and $\frac{{E}_{{xy}}^{3\omega }}{{E}_{{xx}}^{3}}$ as a function of the square of conductivity (${\sigma }_{{xx}}^{2}$), respectively. The error bars reflect the standard fitting error of ${E}_{{xy}}^{2\omega }$ versus ${E}_{{xx}}^{2}$ (${E}_{{xy}}^{3\omega }$ versus ${E}_{{xx}}^{3}$), respectively. **d** Schematic geometry of an angular-dependent measurement device. Right inset, optical image of disk-like device. Scale bar: 5 μm. **e** Angular dependence of the first-order longitudinal voltage (${V}_{{xx}}$) and second-, third-, fourth-, and fifth-harmonic transverse voltages measured at 1.5 K and 10 K, respectively. The solid lines are fits to sinusoidal functions with one-fold (360° period) and two-fold (180° period) angular symmetries. Odd-order transverse components exhibit a pronounced two-fold angular symmetry, while even-order transverse components show a nearly one-fold response in both the superconducting and CDW states.

A schematic illustration of the vortex dynamics is shown in Fig. 3a. Due to CsV_3_Sb_5_’s layered structure and short coherence length^43^, vortex dynamics exhibit strong two-dimensional behavior and enhanced thermal fluctuations. The small coherence length promotes thermal vortex–antivortex excitations, making the system a candidate for a Berezinskii–Kosterlitz–Thouless (BKT) transition, where vortex motion dominates dissipation near ${T}_{{BKT}}$. As temperature approaches the superconducting transition, thermal energy unbinds vortex–antivortex pairs, creating free vortices that move under an applied current, resulting in dissipation and finite resistance. Even without an external magnetic field, moving vortices generate a transverse nonlinear voltage via an intrinsic vortex Hall effect^44–47^. Near ${T}_{c}$, where vortex–antivortex fluctuations, vortex mobility, and pinning become particularly pronounced, the nonlinear current–velocity relation of the vortices can generate strong higher-harmonic transverse responses. In particular, broken inversion symmetry and asymmetric vortex pinning can introduce a nonreciprocal component of the vortex motion and thereby contribute to the second-harmonic transverse voltage, whereas nonlinear vortex dynamics may give rise to a third-harmonic response even without such rectification. The strong enhancement of the nonlinear transverse signals near ${T}_{c}$ is therefore consistent with nonlinear vortex dynamics superimposed on the underlying Berry-geometric response of the CDW state.

### Nonlinear transport and band geometry scaling

While vortex motion significantly shapes nonlinear transport near the superconducting transition, these dynamical phenomena must be distinguished from intrinsic contributions stemming from electronic band geometry. To evaluate these separate effects, the scaling behavior of nonlinear Hall susceptibilities with longitudinal conductivity is analyzed, as depicted in Fig. 3b, c. Over a broad temperature range, the third-order nonlinear Hall susceptibility displays an approximately linear relationship with ${\sigma }_{{xx}}^{2}$. For a BCQ-mediated response, the third order nonlinear Hall susceptibility is expressed phenomenologically as χ^3^^ω^ ∝ Qσ_xx_, using ${\sigma }_{{xx}}\propto \tau$ within the Drude picture and where Q denotes the BCQ. The experimentally observed χ^3^^ω ^∝ σ^2^_xx_ scaling therefore suggests that effective BCQ contribution itself evolves with temperature approximately in proportion to σ_xx_ over the measurement regime. Such evolution is plausible in CsV_3_Sb_5_ because its berry curvature distribution and higher-order moments are strongly reconstructed by the temperature-dependent CDW and loop-current orders. Accordingly, the observed scaling should not be interpreted solely as ${\tau }^{2}$ dependence, but rather as reflecting the combined evolution of scattering and the underlying Berry geometry^5,10^. In contrast, the second-order nonlinear Hall susceptibility exhibits a substantially weaker, sublinear dependence on ${\sigma }_{{xx}}^{2}$, which cannot be captured by a simple skew-scattering-dominated contribution expected to scale approximately as ${\sigma }_{{xx}}^{2}$. This deviation may be associated with the reconstructed CDW state, where the BCD can evolve with the CDW order parameter, Fermi-surface topology, and chemical potential, while contributing a comparatively weakly scattering-time-dependent component to the nonlinear Hall susceptibility. Even though pristine bulk CsV_3_Sb_5_ maintains global inversion symmetry, spatial inversion symmetry can be broken in thin-film architectures due to asymmetric substrate interfaces, device environments, or surface electric fields, alongside further symmetry reduction from CDW or nematic order. These symmetry-lowering mechanisms allow a nonzero BCD, establishing a microscopic foundation for the detected second-order response.

### Berry curvature quadrupole from chiral loop-current order

**Fig. 4:**
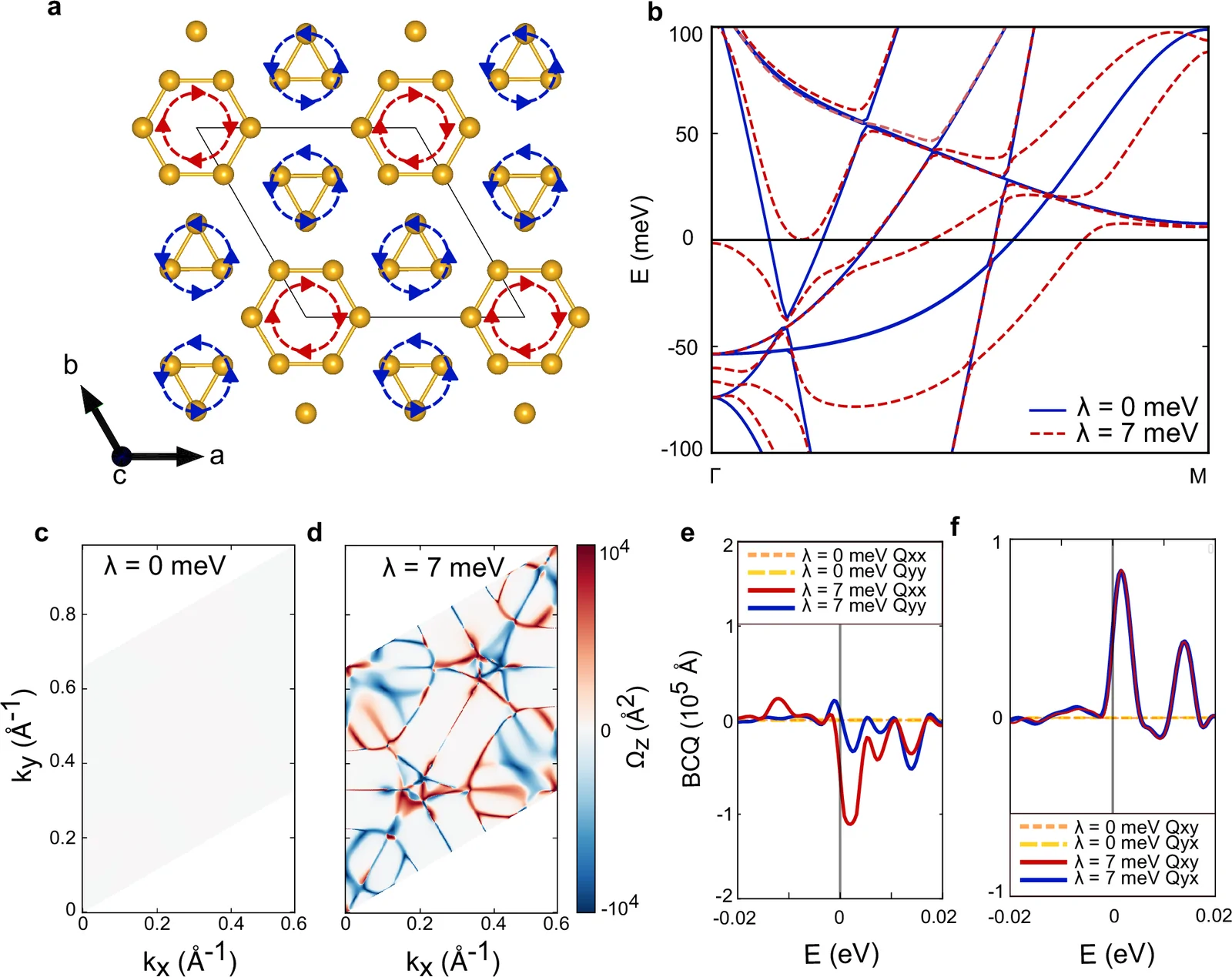
Berry curvature quadrupole (BCQ) induced by chiral loop-current order. **a** Schematic illustration of the complex CDW phase in CsV_3_Sb_5_. Three V sublattices of Kagome network are shown with the 3Q lattice distortions and the associated chiral loop currents. **b** Electronic band structure along the Γ−M direction calculated without (λ= 0 meV, blue solid lines) and with (λ= 7 meV, red dashed lines) loop-current order. The loop-current order reconstructs the low-energy electronic structure and lifts the time reversal band degeneracies near Fermi energy. Here, the λ denotes the loop current strength. **c**, **d** Berry curvature distribution of CsV_3_Sb_5_ in the CDW phase without (λ= 0 meV) and with loop currents (λ= 7 meV). Finite Berry curvature emerges only in the presence of loop-current order, indicating time-reversal-symmetry breaking. **e**, **f** Fermi energy dependence of diagonal and off-diagonal components of BCQ in the absence (λ= 0 meV, dashed line) and presence of loop-current order (λ= 7 meV, solid lines). The introduction of loop currents dramatically enhances both diagonal and off-diagonal BCQ components, generating pronounced peaks in the vicinity of the calculated Fermi energy (defined by E = 0), whereas the BCQ remains nearly vanished in the absence of loop-current order. The components carry an extra third index ‘z’ to denote that only the z component of the Berry curvature is relevant for our 2D Kagome lattice.

To gain microscopic insight into the band-geometric origin of the nonlinear Hall response, we performed first-principles calculations for the complex CDW phase of CsV_3_Sb_5_, which has been shown to be a 3Q ordered phase^37,48^. The complex CDW order parameter can be decomposed into real and imaginary components. The real component (rCDW) leads to either trihexagonal or star of David type atomic distortions within the V Kagome lattice, while the imaginary part of the order parameter (iCDW) is associated with the chiral flux generated by time reversal symmetry breaking loop currents. Starting from the experimentally determined CDW supercell structure reported by X-ray diffraction studies^37^, we constructed a Wannier-based tight-binding model and incorporated loop-current order through the nearest neighbor hopping interactions between V atoms as ${t}_{{ij}}\to {t}_{{ij}}+\lambda {e}^{i{\phi }_{{ij}}}$ where λ denotes the loop-current strength and ϕ is the directional phase assigned to each specific bond to dictate the flow of the loop currents (see Supplementary Note 5). To isolate the contribution of the purely imaginary hopping channel, we set ${\phi }_{{ij}}=\pm \pi /2$, such that ${\lambda e}^{{i\phi }_{{ij}}}=\pm i\lambda$. The signs are assigned according to the prescribed loop-current pattern around the Kagome plaquettes. This construction explicitly breaks time-reversal symmetry while introducing only a single parameter λ, controlling the strength of the chiral flux phase. Guided by the experimentally relevant energy scale associated with the CDW reconstruction, we use $\lambda=7{\rm{meV}}$ for the calculations presented below. A schematic illustration of the 3Q CDW ordered state with chiral flux phase is shown in Fig. 4a. The calculated electronic band structures along the high symmetric Γ−M direction are displayed in Fig. 4b. In the absence of loop-current order ($\lambda=0{\rm{meV}}$), the electronic structure is determined solely by the static CDW reconstruction. Introducing the chiral flux term ($\lambda=7{\rm{meV}}$) further reconstructs the low-energy bands, breaks time-reversal symmetry, and lifts symmetry-protected or accidental degeneracies near the Fermi level. This evolution is illustrated by the $\lambda=0{\rm{meV}}$ bands shown as black solid lines and the $\lambda=7{\rm{meV}}$ bands shown as red dashed lines. The modification of the band structure is accompanied by a pronounced reconstruction of the Berry curvature. As shown in Fig. 4c, d, the loop-current term generates substantial out-of-plane Berry-curvature hotspots, ${\varOmega }_{{{\rm{z}}}}\left({{\boldsymbol{k}}}\right)$, near the reconstructed low-energy bands. Under time reversal, ${\varOmega }_{{{\rm{z}}}}\left({{\boldsymbol{k}}}\right)=-{\varOmega }_{{{\rm{z}}}}\left(-{{\boldsymbol{k}}}\right)$ whereas inversion symmetry imposes ${\varOmega }_{{{\rm{z}}}}\left({{\boldsymbol{k}}}\right)={\varOmega }_{{{\rm{z}}}}\left(-{{\boldsymbol{k}}}\right)$. When both symmetries are present, these constraints require ${\varOmega }_{{{\rm{z}}}}\left({{\boldsymbol{k}}}\right)=0$. The chiral flux phase explicitly removes the time-reversal constraint and thereby permits a qualitatively different Berry-curvature distribution; rather than the mere presence of local finite Berry curvature alone, it is the loop-current-induced removal of the time-reversal symmetry constraint and the resulting reconstruction of ${\varOmega }_{{{\rm{z}}}}\left({{\boldsymbol{k}}}\right)$ that establish the connection between the chiral flux order and the enhanced Berry geometry of the CDW state.

Having identified the imaginary component of CDW order as a microscopic origin of the reconstructed Berry curvature in CsV_3_Sb_5_, we next evaluate the band-geometric quantities relevant to nonlinear Hall transport. Mathematically, the third-order current density ${j}^{3\omega }$ can be expressed as:${j}_{a}^{3\omega }={\chi }_{{abcd}}{E}_{b}{E}_{c}{E}_{d}$, where the susceptibility ${\chi }_{{abcd}}$ is proportional to the BCQ which is the integration of the second derivative of the Berry curvature over the occupied states^7^, ${\chi }_{{{\rm{BCQ}}}}^{(3)}\propto \frac{{e}^{4}{\tau }^{2}}{{\hslash }^{3}}Q$, where τ is the transport relaxation time. For the quasi-two-dimensional Kagome electronic structure, where the relevant Berry-curvature component is ${\varOmega }_{z}$, the BCQ can be expressed as ${Q}_{{ab}}={\sum}_{n}\int \left[d{{\boldsymbol{k}}}\right]{f}_{0n}\left({{\boldsymbol{k}}}\right)\frac{{\partial }^{2}{\Omega }_{n,z}\left({{\boldsymbol{k}}}\right)}{\partial {k}_{a}\partial {k}_{b}},a,b=x,y$. Accordingly, the relevant tensor components are ${Q}_{{xx}}$, ${Q}_{{yy}}$, and ${Q}_{{xy}}={Q}_{{yx}}$. The calculated Fermi-energy dependence of these components is shown in Fig. 4e, f. In the absence of loop-current order ($\lambda=0{\rm{meV}}$), the calculated BCQ components remain negligibly small throughout the investigated energy window. In contrast, introducing the chiral flux phase with $\lambda=7{\rm{meV}}$ produces pronounced BCQ peaks near the calculated Fermi level and enhances both the diagonal and off-diagonal components by several orders of magnitude. In particular, the inequivalence between ${Q}_{{xx}}$ and ${Q}_{{yy}}$ reflects the strong rotational anisotropy of the reconstructed electronic state, consistent with the emergence of nematicity. The mixed components satisfy ${Q}_{{xy}}={Q}_{{yx}}$, as expected from the equality of mixed momentum derivatives of the Berry curvature. These calculations identify the time-reversal-symmetry-breaking loop-current order as an efficient microscopic mechanism for generating a large BCQ in the reconstructed CDW state. Combined with the experimentally observed third-order nonlinear Hall response and its approximate ${\sigma }_{{xx}}^{2}$ scaling, the results establish a microscopic connection between the chiral CDW state, enhanced Berry-curvature multipoles, and the third-order nonlinear Hall effect.

We also calculated the Berry Connection Polarizability (BCP), an intrinsic band geometric quantity that measures the susceptibility of the Berry connection to an external electric field, which can contribute to the third-order nonlinear susceptibility^49^ (Supplementary Note 6). Unlike the BCQ, the BCP tensor is not restricted by time-reversal symmetry and remains finite both with and without loop-current order (Supplementary Fig. 14), although its magnitude is moderately enhanced in the chiral flux phase. Importantly, the third-order Hall conductivity arising from BCP scales linearly with the scattering time or longitudinal Drude conductivity, whereas the BCQ contribution scales quadratically. Based on the experimental scaling relations discussed earlier, this suggests that BCQ is the dominant intrinsic mechanism driving the third-order Hall response in the CDW phase.

The third-order nonlinear Hall effect is related to the BCQ because it arises from the second-order perturbation of the electron distribution function, which mathematically couples the electric field to the second-order gradient (the quadrupole) of the Berry curvature in momentum space. In contrast, the second-order effect only probes the first-order gradient (the dipole). However, neither the centrosymmetric SoD/Tri-hexagonal CDW structural distortions nor the loop-current order necessarily breaks inversion symmetry in bulk CsV_3_Sb_5_. Therefore, provided that inversion symmetry is preserved in the combined CDW and loop-current state, a bulk electric-dipole second-order nonlinear response is symmetry forbidden. Consequently, the observation of a finite second-order response would require an additional source of inversion-symmetry breaking, such as surface or interface symmetry breaking, strain, stacking reconstruction, or a lower-symmetry electronic state. Given the nanometer thickness of our devices ( ~ 10 nm), the substantially enhanced surface-to-volume ratio in this quasi-two-dimensional limit makes the surface contribution particularly significant. The resulting surface-driven nonlinear Hall signal is expected to be strongly enhanced near the CDW transition, where the surface electronic states undergo substantial reconstruction.

While the second order effect is found to be more symmetry restrictive in this system, the third-order effect, however, is less restricted by inversion symmetry and is found to thrive on the BCQ contributions as a consequence of the loop currents. Moreover, the relationship between the electric field E and current density J near the SC transition may follow a power law: $V\propto {I}^{\alpha }$, where α changes from α>3 below the transition to α=1 in the normal state. The value of α=3 at ${T}_{{BKT}}$ is a fundamental result of the universal jump in the superfluid density, which dictates how the system transitions from a state of bound vortex pairs to a state of free, dissipating vortices (See Supplementary Note 1). At the transition point where $\alpha \approx 3$, the voltage is proportional to the cube of the current ($V\propto {I}^{3}$), although this governs the longitudinal response, it leads to a massive enhancement of the third-order nonlinear Hall signal ${V}_{{xy}}^{3\omega }$ when the vortex motion has a transverse component (see Supplementary Note 1).

We fabricated disk-shaped devices to directly probe the intrinsic rotational symmetry of the nonlinear response (Fig. 3d). We observe a pronounced two-fold angular symmetry in the odd-order transverse signals, while the even-order transverse components exhibit a nearly one-fold response in both the superconducting and charge-density-wave states, as shown in Fig. 3e (Original data are shown in Supplementary Figs. 5-6). This distinct symmetry dichotomy reflects a spontaneous reduction of the underlying rotational symmetry and is understood in terms of electronic nematicity emerging from the Kagome lattice. In particular, the two-fold symmetry of the odd-order nonlinear Hall response indicates a nematic electronic state that breaks the native threefold (or sixfold) rotational symmetry, consistent with previous transport and spectroscopic studies reporting C_2_ symmetry in CsV_3_Sb_5_^26,33^. The unidirectional order has a preferred axis (a “nematic director”) and is invariant under a rotation of 180°. In contrast, the nearly one-fold angular dependence of the signed second-order response is compatible with a BCD contribution. In two dimensions, the BCD transforms as an in-plane polar vector and therefore selects a directed axis, such that the corresponding second-order response can reverse sign upon a 180° rotation and recover only after 360°. The coexistence of two-fold odd-order and one-fold even-order responses therefore suggests that the nonlinear transport probes distinct tensorial components of the symmetry-broken electronic state.

### Vortex dynamics effect on the nonlinear response in CsV_3_Sb_5_

**Fig. 5:**
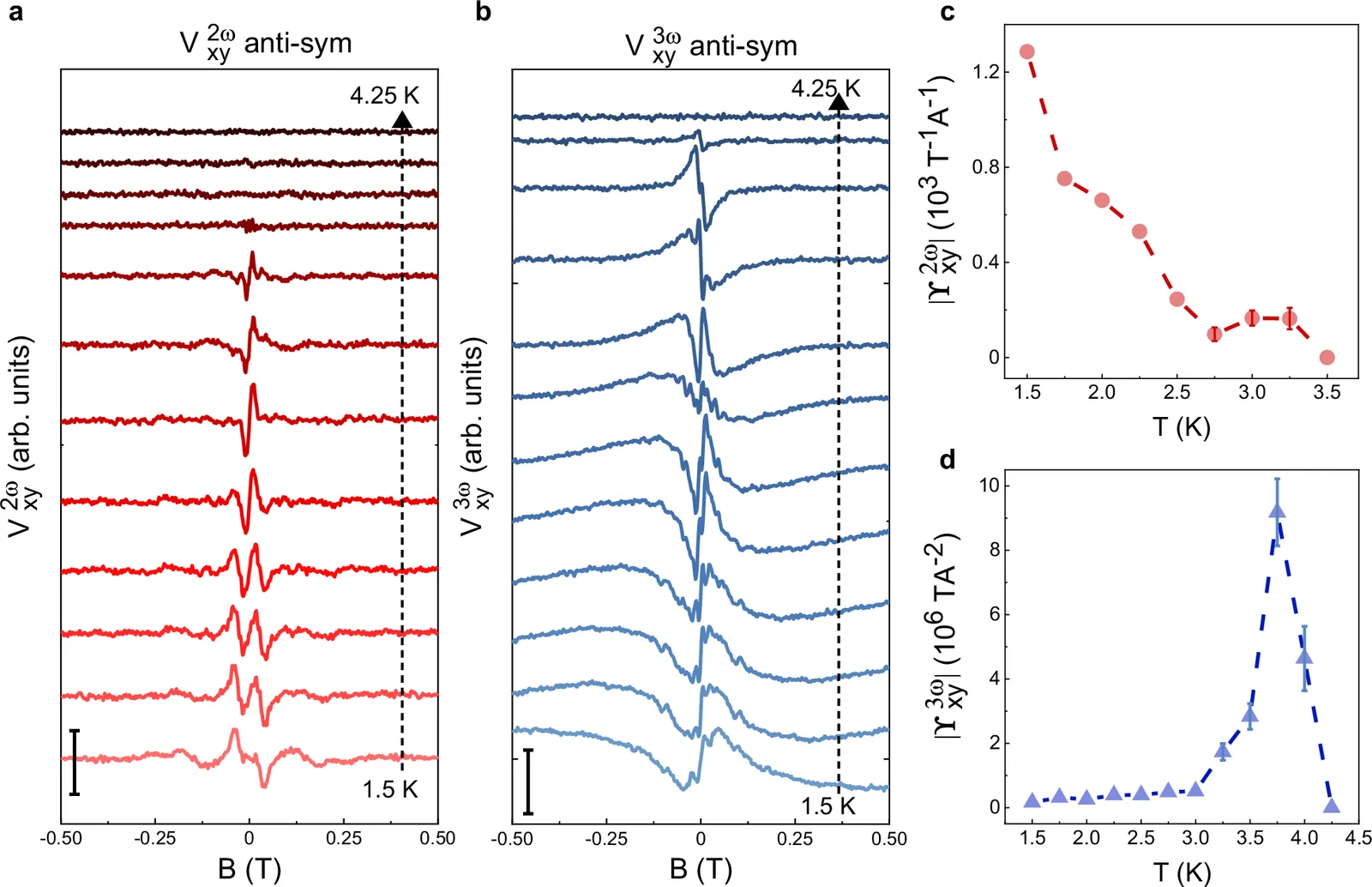
Nonreciprocal transverse transport in CsV_3_Sb_5_, driven by vortex motion. **a**, **b** Anti-symmetric part of ${V}_{{xy}}^{2\omega }$ and ${V}_{{xy}}^{3\omega }$ evolution with respect of out-of-plane magnetic field. Scale bar: 1 μV. **c**, **d** Extracted ${\gamma }_{{xy}}^{2\omega }$ and ${\gamma }_{{xy}}^{3\omega }$ as a function of temperature. The anti-symmetric peak feature of both ${V}_{{xy}}^{2\omega }$ and ${V}_{{xy}}^{3\omega }$ vanishes above the superconducting transition temperature of 4.2 K. Here, the ${\gamma }_{{xy}}^{2{{\rm{\omega }}}}$ and ${\gamma }_{{xy}}^{3{{\rm{\omega }}}}$ are defined as ${\gamma }_{{xy}}^{2{{\rm{\omega }}}}=\frac{2{V}_{{xy}}^{2\omega }}{B{I}^{2}{R}_{{xx}}}$ and ${\gamma }_{{xy}}^{3{{\rm{\omega }}}}=\frac{4{V}_{{xy}}^{3\omega }}{B{I}^{3}{R}_{{xx}}}$, respectively. The error bars represent the uncertainty in determining the field B and ${R}_{{xx}}$ from (**a**, **b**) due to the gradual transition.

To further elucidate the role of vortex dynamics in the nonlinear Hall response, we measured the magnetic-field dependence of the second- and third-harmonic transverse signals under a fixed AC current. Vortex dynamics near the superconducting transition can contribute to both the second- and third-order nonlinear transverse responses. As the temperature approaches ${T}_{c}$, the superconducting vortex state becomes increasingly dynamic, with enhanced vortex mobility, depinning, and vortex–antivortex fluctuations. The measured nonlinear transverse responses contain both a relatively stationary electronic contribution and an additional dynamical contribution associated with vortex motion. Previous studies of nonreciprocal charge transport in CsV_3_Sb_5_ have primarily focused on longitudinal configurations within the charge-ordered phase^22,50^. In particular, the observed second-harmonic longitudinal resistance has been interpreted in terms of electronic magnetochiral anisotropy (eMChA) and vortex-driven rectification^22,50^. In contrast to those longitudinal measurements, our configuration probes the transverse nonlinear response ${V}_{{xy}}^{2\omega }$ and ${V}_{{xy}}^{3\omega }$ under out-of-plane magnetic field. To clarify the underlying mechanism of the nonlinear transverse response, the raw second- and third-harmonic transverse signals were decomposed into symmetric and antisymmetric components with respect to magnetic field, defined as ${V}^{{\rm{odd}}}(B)=\frac{V(B)-V(-B)}{2}$, and ${V}^{{\rm{even}}}(B)=\frac{V(B)+V(-B)}{2}$. This separation allows us to distinguish between field-odd and field-even nonlinear contributions. The first-order magnetoresistance ${R}_{{xx}}$ (Supplementary Fig. 10a) is symmetric with respect to magnetic field and vanishes near 4 K, defining the superconducting transition temperature. Upon entering the superconducting state, a pronounced antisymmetric component emerges in both ${V}_{{xy}}^{2\omega }$ and ${V}_{{xy}}^{3\omega }$ (Fig. 5a, b, Supplementary Fig. 9). Importantly, the antisymmetric peaks vanish at the superconducting transition temperature, establishing a direct correlation with superconductivity. The odd dependence on magnetic field can be understood within the framework of vortex dynamics: in the mixed state, the transverse electric field generated by moving vortices follows the flux-flow relation^51–54^ $E=B\times {v}_{{{\rm{vortex}}}}$, so that reversing the magnetic field reverses the vortex polarity and consequently the sign of the transverse voltage. The magnetic field controls the vortex density and pinning landscape, while the AC current drives vortex motion via the Lorentz force. When the vortex dynamics becomes nonlinear due to asymmetric or velocity-dependent pinning, higher-harmonic transverse voltages can be generated. The disappearance of the antisymmetric component above ${T}_{c}$ therefore identifies nonlinear vortex motion as its dominant origin.

Within this framework, the transverse electric field can be expanded as

1 $${E}_{y}\propto B\left({\mu }_{1}J+{\mu }_{2}{J}^{2}+{\mu }_{3}{J}^{3}+\cdots \right)$$

where nonlinear vortex mobility coefficients ${\mu }_{2}$ and ${\mu }_{3}$ give rise to the second- and third-harmonic signals, respectively (see Supplementary Note 2). The simultaneous enhancement of both ${V}_{{xy}}^{2\omega }$ and ${V}_{{xy}}^{3\omega }$ near the superconducting transition indicates intrinsically nonlinear vortex dynamics in CsV_3_Sb_5_. In addition to the antisymmetric contribution, a finite symmetric component is also resolved in both harmonic channels (Supplementary Fig. 10b-c). Unlike the antisymmetric signal, the symmetric part does not directly reflect vortex polarity reversal and may originate from higher-order electronic nonlinearities or magnetoresistance mixing effects that are even in magnetic field. The coexistence of symmetric and antisymmetric components therefore indicates that the nonlinear transverse response contains both electronic and vortex-related contributions, with vortex motion dominating the antisymmetric channel in the superconducting state.

As shown in Fig. 5a, b, the antisymmetric components of ${V}_{{xy}}^{2\omega }$ and ${V}_{{xy}}^{3\omega }$ exhibit pronounced field-dependent fluctuations concentrated around B=0 in the superconducting regime, indicating that both harmonic channels are sensitive to vortex dynamics. Here, we defined ${\gamma }_{{xy}}^{2{{\rm{\omega }}}}$ and ${\gamma }_{{xy}}^{3{{\rm{\omega }}}}$ to normalize the measured second and third order non-linear voltages, in the equation of ${\gamma }_{{xy}}^{2{{\rm{\omega }}}}=\frac{2{V}_{{xy}}^{2\omega }}{B{I}^{2}{R}_{{xx}}}$ and ${\gamma }_{{xy}}^{3{{\rm{\omega }}}}=\frac{4{V}_{{xy}}^{3\omega }}{B{I}^{3}{R}_{{xx}}}$, respectively (see Supplementary Note 2). As displayed in Fig. 5c, the second-order coefficient ${\gamma }_{{xy}}^{2{{\rm{\omega }}}}$ decreases monotonically with increasing temperature, similar to the trend reported in the longitudinal second-order nonreciprocal transport of CsV_3_Sb_5_^50^. In contrast, the third-order coefficient ${\gamma }_{{xy}}^{3{{\rm{\omega }}}}$ (Fig. 5d) exhibits a pronounced enhancement near the superconducting transition before collapsing above ${T}_{c}$, which is similar to the longitudinal second-order nonreciprocal transport in superconductor NbSe_2_^55^. The contrasting temperature dependences suggest that the two harmonic responses probe different aspects of the vortex dynamics.

Near the superconducting transition, vortex dynamics can introduce spatially and temporally varying supercurrents, magnetic fields, strain, and electrochemical-potential gradients. These perturbations can modulate the local electronic structure and, consequently, the effective Berry-curvature multipoles. Phenomenologically, this effect may be represented as: ${Q}_{{ab}}^{\left(0\right)}+\delta {Q}_{{ab}}({{\rm{r}}},{{\rm{t}}})$, where ${Q}_{{ab}}^{\left(0\right)}$ denotes the quasi-static BCQ associated with the underlying symmetry-broken electronic structure, while $\delta {Q}_{{ab}}({{\rm{r}}},{{\rm{t}}})$ represents a possible vortex-induced modulation of the effective BCQ. Correspondingly, the third-order transverse response may be expressed phenomenologically as: ${V}_{{xy},0}^{3\omega }+\delta {V}_{{xy},{{\rm{vortex}}}}^{3\omega }$, where the first term represents the underlying electronic nonlinear Hall response, including the BCQ contribution, while the second captures the additional fluctuating nonlinear response associated with vortex dynamics. This decomposition does not require the vortices themselves to possess a BCQ. Instead, vortex-induced spatial and temporal perturbations can couple to and modulate the symmetry-allowed nonlinear electronic response. Close to the BKT regime, the vortex contribution exhibits a strong cubic dependence on the applied current, which can give rise to pronounced fluctuations in ${V}_{{xy}}^{3\omega }$ (see Supplementary Note 1).

By contrast, the second-order response probes a distinct symmetry channel. Dynamic vortex–antivortex motion can itself generate a rectified transverse response when vortex and antivortex trajectories, mobilities, or pinning potentials are asymmetric. However, increasingly stochastic vortex motion can reduce the coherent component of this rectification through cancellation between vortex configurations. Thus, the experimentally observed decrease of the second-order fluctuation amplitude toward the superconducting transition, accompanied by an enhancement of the third-order fluctuations, can be interpreted as a crossover from coherent second-order rectification toward strongly nonlinear vortex dynamics that couple preferentially to the third-harmonic channel.

Our study demonstrates the unique capability of third-order nonlinear Hall measurements, mediated by the BCQ, to probe electronic nematic phases in the Kagome superconductor CsV_3_Sb_5_. Unlike conventional second-order nonlinear Hall responses, which rely on specific polar symmetries often suppressed or absent in superconducting fluctuations, the third-order response emerges as the dominant contribution in both the charge density wave (CDW) and superconducting states. The third-order nonlinear response dominance arises from the synergy between BCQ-driven quantum geometric contributions and the intrinsic nonlinear dynamics of the superconducting order parameter, particularly the cubic current (*I*^3^) dependence inherent in vortex-unbinding processes. The BCQ exhibits heightened sensitivity to local symmetry-breaking fluctuations, such as transient time-reversal symmetry breaking and strong internal field gradients (*∇* E), making it exceptionally well-suited to detect and characterize nematic electronic environments. Our findings establish BCQ-mediated third-order nonlinear Hall response as a powerful and versatile tool for investigating correlated electronic phases, offering enhanced sensitivity to fluctuating and inhomogeneous nematic orders where conventional even-order responses may fall short. This approach opens new pathways for exploring symmetry-broken states in quantum materials with strong electronic correlations.

## Methods

### Device fabrication and transport measurement

Thin CsV_3_Sb_5_ flakes were mechanically exfoliated from commercially available CsV_3_Sb_5_ bulk crystals using scotch tape and transferred onto SiO_2_/Si substrates (280 nm SiO_2_). For nonlinear transport measurements, standard electron-beam lithography was employed to define multi-terminal Hall-bar or disk-shaped device geometries. After pattern development, Ti/Au (typically 5 nm/70 nm) electrodes were deposited by electron-beam evaporation, followed by lift-off. Devices were loaded into helium cryostat Oxford TeslatronPT and measured using lock-in amplifiers (Stanford Research Systems SR830, and Sine Scientific Instruments OE1022D). Consistent results were obtained at multiple excitation current frequencies generated from Keithley 6221. The phases of first- and second-, third-, fourth-, fifth-, and sixth-harmonic voltage signals were set to be 0°, 90°, 0°, 90°, 0°, 90°, respectively.

### Theoretical calculation

The electronic structure of CsV_3_Sb_5_ has been calculated using projector augmented-wave method^56,57^ implementation of density functional theory (DFT) within the Vienna Ab-initio Simulation Package (VASP)^58,59^. The initial structure is considered to be a commensurate 2×2×4 supercell with 3Q bond order CDW distortions^48^ as found in X-ray diffraction studies^37^. To negate the effect of the periodic images of the slab as implemented within VASP, a vacuum layer of 20 Å has been added in the c direction. Perdew-Burke-Ernzerhof potentials^60,61^ were used for the exchange correlation functional. For the self-consistent calculation, a k-space grid of 8×8×1 was used for the unit cell. The cut-off energy for the plane wave basis was taken to be 500 eV, and the atomic coordinates have been optimized until the forces on the atoms are less than 0.01 eV/${{{\AA }}}$. In order to add the 3Q chiral flux phase or loop current^62,63^, the ab initio results have been mapped onto a tight binding model using maximally localized Wannier functions as implemented in WANNIER90^64^ (see Supplementary Note 5). The atomic orbital basis for tight binding is considered to be the p orbitals of the Sb and d orbitals of the V atoms. The Berry curvature and its higher order moments have been calculated using Kubo formula as implemented in the WANNIERBERRI (see Supplementary Note 6)^65^.

## Supplementary information

Supplementary Information

Transparent Peer Review file

## Source data

Source Data

## Data Availability

The authors declare that the data supporting the findings of this study are available within the paper and its Supplementary Information files. Source data are provided with this paper.
