# Supplementary material for: Berry curvature multipoles in Kagome superconductor CsV3Sb5
Source: Nat Commun. 2026 Sep 10;17:10683. doi: 10.1038/s41467-026-77618-6 (PMC13654926; doi:10.1038/s41467-026-77618-6)
Supplement: Supplementary file 1 [file 41467_2026_77618_MOESM1_ESM.pdf]

Supplementary Information

Berry Curvature Multipoles in Kagome Superconductor CsV_3Sb_5

Content:

Supplementary Notes 1-6

Supplementary Figs. 1-14

Supplementary Note 1. The relationship between α(T) and superfluid density

In the Berezinskii–Kosterlitz–Thouless (BKT) transition framework for two-dimensional superconductors, the power-law exponent α(T) is not an arbitrary constant; it is directly proportional to the superfluid density $n_s(T)$. The theoretical relation is given by:

$$\alpha(T) = 1 + \frac{\pi\hbar^2 n_s(T)}{2m^* k_B T}, \qquad \text{(S1)}$$

and the BKT theory proves that a 2D system cannot support long-range order at finite temperatures due to vortex fluctuations. However, it can support "quasi-long-range order" if the superfluid density is high enough to keep vortex-antivortex pairs bound.

The theory predicts that the BKT transition occurs exactly when the ratio of the superfluid density to temperature reaches a universal value: $\frac{n_s(T_{BKT})}{T_{BKT}} = \frac{4m^* k_B}{\pi\hbar^2}$. When substituting this "universal jump" ratio back into the equation for the exponent $\alpha(T)$, the constants cancel out perfectly: $\alpha(T) = 1 + \frac{\pi\hbar^2}{2m^* k_B}\left(\frac{4m^* k_B}{\pi\hbar^2}\right) = 1 + \frac{4}{2} = 3$. This result is universal because it does not depend on the specific material properties, the effective mass of the carriers, or the chemical composition of the superconductor, and it depends only on the topological nature of 2D phase fluctuations.

The universal value $\alpha(T_{\mathrm{BKT}}) = 3$ further implies a cubic longitudinal response, $V_{xx} \propto I^3$, near the BKT transition. Although BKT physics itself governs the longitudinal nonlinear response rather than a transverse voltage, a finite transverse vortex motion, such as that associated with the vortex Hall effect, can transfer this cubic nonlinearity to the transverse channel, yielding $V_{xy} \approx \tan(\theta_v) V_{xx} \propto I^3$, where θ_v represents the effective vortex Hall angle. Under AC excitation, $I(t) = I_0 \sin(\omega t)$, the generated transverse voltage response, $V_{xy}(t) \propto I_0^3 \sin^3(\omega t)$, reflects this cubic current dependency through $\sin^3(\omega t) = \frac{3}{4}\sin(\omega t) - \frac{1}{4}\sin(3\omega t)$. Thus, BKT-related vortex dynamics may contribute to the enhancement of $V_{xy}^{3\omega}$ near the superconducting transition.

Supplementary Note 2. Vortex-induced nonlinear transverse response under finite magnetic field

In the vortex flow regime, the dynamics of a single vortex are governed by the force balance equation

$$\eta \mathbf{v} = \mathbf{F}_L + \mathbf{F}_{\mathrm{pin}}(\mathbf{v}), \qquad \text{(S2)}$$

where η is the viscous drag coefficient, $\mathbf{F}_L$ is the Lorentz force, and $\mathbf{F}_{\mathrm{pin}}$ represents the pinning force, which may depend nonlinearly on the vortex velocity. For a magnetic field applied along the out-of-plane direction, $\mathbf{B} = B\hat{z}$, and a transport current along the x-direction, $\mathbf{J} = J_x\hat{x}$, the Lorentz force acting on a vortex is

$$\mathbf{F}_L = \Phi_0 \mathbf{J} \times \hat{z} = \Phi_0 J_x \hat{y}, \qquad \text{(S3)}$$

where $\Phi_0 = h/2e$ is the superconducting flux quantum. When the pinning force contains nonlinear velocity-dependent terms, it can be phenomenologically expanded as

$$\mathbf{F}_{\mathrm{pin}} = -\alpha_1 \mathbf{v} - \alpha_2 \mid \mathbf{v} \mid \mathbf{v} - \alpha_3 \mid \mathbf{v} \mid^2 \mathbf{v} + \cdots . \qquad \text{(S4)}$$

Solving the force balance equation perturbatively in the small-current limit yields a nonlinear expansion of the vortex velocity in powers of current:

$$\mathbf{v} = \mu_1 J_x + \mu_2 J_x^2 + \mu_3 J_x^3 + \cdots , \qquad (S5)$$

where $\mu_1 = \Phi_0/(\eta + \alpha_1)$, and the higher-order coefficients μ_2 and μ_3 arise from nonlinear pinning and velocity-dependent dissipation.

The electric field generated by moving vortices follows the standard flux-flow relation $\mathbf{E} = \mathbf{B} \times \mathbf{v}$. For $\mathbf{B} = B\hat{z}$, the transverse electric field component becomes $E_y = Bv_x$. Substituting the nonlinear velocity expansion gives

$$E_y = B(\mu_1 J_x + \mu_2 J_x^2 + \mu_3 J_x^3 + \cdots). \qquad \text{(S6)}$$

Under an ac current excitation $J_x(t) = J_0 \sin(\omega t)$, we obtain $J_x^2(t) = \frac{J_0^2}{2}(1 - \cos(2\omega t))$, $J_x^3(t) = \frac{3J_0^3}{4}\sin(\omega t) - \frac{J_0^3}{4}\sin(3\omega t)$.

Substituting these into the expression for $E_y(t)$, we find that: 1) The quadratic term produces a second-harmonic transverse electric field $E_y^{2\omega} \propto B\mu_2 J_0^2$; 2) The cubic term produces a third-harmonic transverse electric field $E_y^{3\omega} \propto B\mu_3 J_0^3$; 3) Both the second- and third-harmonic transverse voltages are intrinsically antisymmetric under magnetic-field reversal. The symmetric response may arise from several mechanisms, including magnetoresistance mixing due to slight probe misalignment, Joule-heating-induced modulation of the longitudinal resistance, or intrinsic higher-order electronic nonlinearities that are even in magnetic field. Since the longitudinal magnetoresistance contains even-in-field contributions, nonlinear current mixing can naturally generate B-even harmonic transverse signals.

Based on the above harmonic decomposition of $J_x^2(t) = \frac{J_0^2}{2}(1 - \cos(2\omega t))$, $J_x^3(t) = \frac{3J_0^3}{4}\sin(\omega t) - \frac{J_0^3}{4}\sin(3\omega t)$, the normalized second- and third-order transverse nonlinear coefficients used in the main text are defined as $\gamma_{xy}^{2\omega} = \frac{2V_{xy}^{2\omega}}{BI^2R_{xx}}$ and $\gamma_{xy}^{3\omega} = \frac{4V_{xy}^{3\omega}}{BI^3R_{xx}}$. Here, the prefactors of 2 and 4 account for the 1/2 and 1/4 amplitudes of the 2ω and 3ω components arising from $J_x^2(t)$ and $J_x^3(t)$, respectively. This normalization removes the explicit current dependence and accounts for the temperature-dependent longitudinal resistance, enabling a direct comparison of the temperature evolution of the second- and third-order nonlinear transverse responses.

Supplementary Note 3. Derivation of high-order transverse nonlinear voltage equations

We consider an ac current applied along the x direction:

$$I_{xx} = I_0 sin(\omega t) \quad , \quad \text{(S7)}$$

then the transverse voltage can be expanded as a power series of the applied current:

$$V_{xy} = R_{yxx}^{(2)} {I_{xx}}^2 + R_{yxxx}^{(3)} {I_{xx}}^3 + R_{yxxxx}^{(4)} {I_{xx}}^4 + R_{yxxxxx}^{(5)} {I_{xx}}^5 + R_{yxxxxxx}^{(6)} {I_{xx}}^6 + \cdots . \qquad \text{(S8)}$$

The second-harmonic transverse signal can be obtained from the quadratic current term as follows:

$$V_{xy}^{2\omega} = R_{yxx}^{(2)} {I_0}^2 Sin^2(\omega t)$$
$$= R_{yxx}^{(2)} {I_0}^2 \left(\frac{1 - Cos(2\omega t)}{2}\right)$$
$$= \frac{1}{2} R_{yxx}^{(2)} {I_0}^2 (1 - Cos(2\omega t))$$
$$= \frac{1}{2} R_{yxx}^{(2)} {I_0}^2 + \frac{1}{2} R_{yxx}^{(2)} {I_0}^2 Sin\left(2\omega t - \frac{\pi}{2}\right). \quad \text{(S9)}$$

The third-harmonic transverse signal can be obtained as follows:

$$V_{xy}^{3\omega} = R_{yxxx}^{(3)} {I_0}^3 Sin^3(\omega t)$$
$$= R_{yxxx}^{(3)} {I_0}^3 \left(\frac{3}{4} Sin(\omega t) - \frac{1}{4} Sin(3\omega t)\right)$$
$$= \frac{3}{4} R_{yxxx}^{(3)} {I_0}^3 Sin(\omega t) + \frac{1}{4} R_{yxxx}^{(3)} {I_0}^3 Sin(3\omega t - \pi). \quad \text{(S10)}$$

The fourth-harmonic transverse signal can be obtained as follows:

$$V_{xy}^{4\omega} = R_{yxxxx}^{(4)} {I_0}^4 Sin^4(\omega t)$$
$$= R_{yxxxx}^{(4)} {I_0}^4 \left(\frac{1 + Sin\left(2\omega t - \frac{\pi}{2}\right)}{2}\right)^2$$
$$= \frac{1}{4} R_{yxxxx}^{(4)} {I_0}^4 \left(1 + 2Sin\left(2\omega t - \frac{\pi}{2}\right) + Sin^2\left(2\omega t - \frac{\pi}{2}\right)\right)$$
$$= \frac{1}{4} R_{yxxxx}^{(4)} {I_0}^4 + \frac{1}{2} R_{yxxxx}^{(4)} {I_0}^4 Sin\left(2\omega t - \frac{\pi}{2}\right) + \frac{1}{4} R_{yxxxx}^{(4)} {I_0}^4 Sin^2\left(2\omega t - \frac{\pi}{2}\right)$$
$$= \frac{1}{4} R_{yxxxx}^{(4)} {I_0}^4 + \frac{1}{2} R_{yxxxx}^{(4)} {I_0}^4 Sin\left(2\omega t - \frac{\pi}{2}\right) + \frac{1}{8} R_{yxxxx}^{(4)} {I_0}^4 \left(1 + Sin\left(4\omega t - \frac{3\pi}{2}\right)\right)$$
$$= \frac{3}{8} R_{yxxxx}^{(4)} {I_0}^4 + \frac{1}{2} R_{yxxxx}^{(4)} {I_0}^4 Sin\left(2\omega t - \frac{\pi}{2}\right)$$
$$+ \frac{1}{8} R_{yxxxx}^{(4)} {I_0}^4 Sin\left(4\omega t - \frac{3\pi}{2}\right). \quad \text{(S11)}$$

The fifth-harmonic transverse signal can be obtained as follows:

$$V_{xy}^{5\omega} = R_{yxxxxx}^{(5)} {I_0}^5 Sin^5(\omega t)$$
$$= R_{yxxxxx}^{(5)} {I_0}^5 Sin^2(\omega t) Sin^3(\omega t)$$

$$= R^{(5)}_{yxxxxxx} {I_0}^5 \left(\frac{1+Sin\left(2\omega t-\frac{\pi}{2}\right)}{2}\right)\left(\frac{3Sin\omega t-Sin3\omega t}{4}\right)$$

$$= \frac{1}{8} R^{(5)}_{yxxxxxx} {I_0}^5 \left(3Sin(\omega t) - Sin(3\omega t) + 3Sin\left(2\omega t-\frac{\pi}{2}\right)Sin(\omega t) - Sin\left(2\omega t-\frac{\pi}{2}\right)Sin3\omega t\right)$$

$$= \frac{1}{8} R^{(5)}_{yxxxxxx} {I_0}^5 \left(3Sin(\omega t) - Sin(3\omega t) - \frac{3}{2}[Sin(3\omega t) - Sin(\omega t)] + \frac{1}{2}[Sin(5\omega t) + Sin(\omega t)]\right)$$

$$= R^{(5)}_{yxxxxxx} {I_0}^5 \left(\frac{5}{8}Sin(\omega t) - \frac{5}{16}Sin(3\omega t) + \frac{1}{16}Sin(5\omega t)\right) \qquad \text{(S12)}$$

The sixth-harmonic transverse signal can be obtained as follows:

$$V^{6\omega}_{xy} = R^{(6)}_{yxxxxxxx} {I_0}^6 Sin^6(\omega t)$$

$$= R^{(6)}_{yxxxxxxx} {I_0}^6 \left(\frac{1-Cos(2\omega t)}{2}\right)^3$$

$$= \frac{1}{8} R^{(6)}_{yxxxxxxx} {I_0}^6 \left(1 - 3Cos(2\omega t) + \frac{3}{2} + \frac{3}{2}Cos(4\omega t) - \frac{3}{4}Cos(2\omega t) - \frac{1}{4}Cos(6\omega t)\right)$$

$$= \frac{1}{32} R^{(6)}_{yxxxxxxx} {I_0}^6 \left(15Sin\left(2\omega t-\frac{\pi}{2}\right) - 6Sin\left(4\omega t-\frac{\pi}{2}\right) + Sin\left(6\omega t-\frac{\pi}{2}\right) + 10\right) \qquad \text{(S13)}$$

Supplementary Note 4. Excluding other extrinsic origins for the observed peak-behavior nonlinear response

Experimentally, the excitation current was supplied by a Keithley 6221 current source. The 6221 possesses a very high output impedance (MΩ-GΩ), which is orders of magnitude larger than the resistance of the superconducting device (Ω-kΩ). Consequently, the actual current flowing through the sample is determined by the current source and remains essentially unchanged by variations in the sample resistance or moderate changes in external series resistance. Therefore, the observed peak cannot be fully attributed to changes in the excitation current caused by variations in circuit resistance. Second, during the measurements, the lock-in sensitivity range was fixed at 50 mV throughout the entire current sweep. Therefore, the peak feature does not originate from sensitivity-range switching.

To further confirm the intact peak behavior regarding lock-in amplifier dynamics, we performed a series of control measurements by systematically varying the lock-in settings. Specifically, we changed:

(i) the input dynamic mode (High Reserve, Normal, and Low Noise);

(ii) the sensitivity range (10 mV, 20 mV, 50 mV, 100 mV, 200 mV, and 500 mV);

(iii) the time constant (30 ms, 100 ms, 300 ms, 1 s, 3 s, and 10 s);

(iv) the filter slope (12, 18, and 24 dB/oct).

As shown in Supplementary Figs. 7-8, No systematic changes in the peak position or overall signal evolution are observed upon varying the lock-in settings. Only the shortest time constant (30 ms) produces larger fluctuations due to reduced signal averaging, while the peak structure itself remains unchanged. Furthermore, the same peak behavior was independently observed using two different lock-in amplifiers (SR830 and OE1022D), demonstrating that the effect is not specific to a particular instrument.

Most importantly, the peak appears only in the superconducting state and disappears completely above T_C (Fig.1e and Fig. 2 in the main text). In addition, the peak evolves systematically with the superconducting transition and is reproducibly observed in multiple devices and measurement configurations. Such behavior is difficult to reconcile with instrumental artifacts arising from lock-in sensitivity settings, reserve modes, input dynamics, filtering parameters, or circuit resistance.

Therefore, these control experiments strongly support that the observed peak is an intrinsic superconductivity-related nonlinear response rather than a measurement artifact.

To further address potential thermal contributions—such as Joule heating or thermoelectric voltages—we carefully analyzed the possible effects and their characteristics.

While we cannot entirely rule out a small symmetric background contribution potentially arising from residual heating or signal mixing, such effects are unlikely to account for the dominant features we observe—namely, the peak structure intimately linked with the superconducting state and the field-antisymmetric nonlinear response dictated by Onsager reciprocity.

First, the nonlinear signals we observe are remarkably stable over a broad excitation-frequency range (Supplementary Fig. 4). Thermal effects, such as Joule heating, typically depend on thermal relaxation processes with characteristic timescales. As a result, signals arising from thermal effects are expected to diminish at higher excitation frequencies due to the finite thermal response time. The weak frequency dependence of our nonlinear response suggests it is unlikely to be driven by thermal relaxation.

Second, the observed nonlinear transverse voltages exhibit sign reversal upon simultaneous reversal of the excitation current direction and the transverse voltage probes. This behavior aligns with the expected symmetry of intrinsic nonlinear Hall effects, which are non-reciprocal and depend on the direction of current flow relative to the crystal axes. Moreover, by systematically changing the input current direction and switching the measurement contacts, we mapped the angular dependence of the nonlinear Hall response. The resulting anisotropic behavior further supports a genuine nonlinear transport origin, as thermal effects, being isotropic, would generally produce a symmetric, non-direction-dependent response.

Third, the temperature dependence of the signal provides additional evidence. The peak in the nonlinear response disappears above the critical temperature T_c. If the signal were primarily due to heating, one would expect it to persist or even intensify in the normal state due to increased resistance and Joule dissipation. The fact that the peak correlates strongly with the superconducting state indicates an intrinsic origin rather than a thermal artifact.

Furthermore, we utilize the symmetry properties of the measurement setup, specifically the transverse Hall geometry and magnetic field reversal, to distinguish between different contributions. Onsager reciprocal relations provide a theoretical foundation here: they imply that, under time-reversal symmetry, certain transport coefficients are symmetric or antisymmetric with respect to magnetic field reversal. In our measurements, the nonlinear Hall response that is antisymmetric with respect to magnetic field reversal is consistent with intrinsic effects related to vortex dynamics and nonlinear Hall effects, rather than thermal biases, which would generally produce symmetric responses independent of field direction.

Supplementary Note 5. Tight binding Hamiltonian using Wannier functions and chiral loop currents

To investigate the Berry multipole contributions to the nonlinear Hall currents, we have modelled the *ab initio* results for the 2×2×4 CsV_3Sb_5 onto a tight binding model using maximally localization Wannier functions[1]. Disentangling the *d* bands of V and *p* bands of Sb atoms from the low energy band manifold, the Wannierized tight binding model have been used to extrapolate the band structure as shown Supplementary Fig. 12. The extrapolated tight binding bands (red dashed lines in Supplementary Fig. 12) are in excellent agreement with the *ab initio* bands (black solid lines in Supplementary Fig. 12), implying that the Wannierized model fully captures the low-energy topology and electronic structure of the CDW state.

The CDW phase in the Kagome lattice of CsV_3Sb_5 have been shown to be a 3Q state[2,3], defined by a complex order parameter. The real part of the CDW order parameter dictates the lattice distortions while the imaginary part manifests as chiral loop current within Kagome network of V atoms that breaks time-reversal symmetry. To determine the Berry curvature related properties, which would otherwise be absent in a centrosymmetrically distorted and time reversal symmetry preserving state, we inject the chiral loop currents[4,5] within the tight binding model and break time reversal symmetry. The loop currents was incorporated into the Wannier Hamiltonian by modifying the nearest neighbor hopping strength between V atoms, t_{ij} as

$$t_{ij} \rightarrow t_{ij} + \lambda e^{i\phi_{ij}}, \qquad \text{(S14)}$$

where λ represents the strength of the loop currents and ϕ_{ij} is the phase factor of the complex CDW order. For simplification, ϕ_{ij} has been kept equal to $\pm\pi/2$ to model only imaginary CDW order parameter on top of the tight binding model of the CDW distorted lattice. Also, to maintain the Hermiticity of the Hamiltonian the condition, $\phi_{ji} = -\phi_{ij}$ have been imposed. Experimentally, the CDW gap observed by STM

and ARPES is typically in the range of 20 - 70 meV[6,7]. The imaginary component, λ, which is responsible for the subtler time-reversal symmetry breaking, is expected to be a fraction of that overall gap scale and therefore a moderate value of $\lambda = 7$ meV has been adopted throughout the calculations. This minimal model enables us to isolate the consequences of loop-current-induced time-reversal-symmetry breaking and to evaluate the associated Berry-curvature multipoles relevant to nonlinear Hall transport.

Supplementary Note 6. The BCQ and Berry Connection Polarizability contributions to the third order hall response

To verify that the introduced loop-current order indeed generates finite Berry curvature, we calculate the momentum-space Berry curvature using the Kubo-formalism implementation in WannierBerri code. Within the tight-binding basis constructed via maximally localized Wannier functions, the momentum-space Berry curvature for the nth band is evaluated using the velocity operators,

$$\Omega_{n,z} = -2\hbar^2 \mathrm{Im} \sum_{m\neq n} \frac{\langle u_n|\widehat{v_x}|u_m\rangle\langle u_m|\widehat{v_y}|u_n\rangle}{(\epsilon_n-\epsilon_m)^2}. \qquad \text{(S15)}$$

The resulting Berry curvature distribution along the high symmetry directions $\Gamma - M - K - \Gamma$ was shown in Supplementary Fig. 13a. In the absence of loop-current order, the Berry curvature is forbidden by time-reversal symmetry. Upon introducing loop currents, pronounced Berry-curvature hotspots emerge near avoided crossings, indicating substantial time-reversal-symmetry breaking and band-structure reconstruction.

The total intrinsic AHC, σ_{xy}, is subsequently obtained by integrating the Berry curvature over the Brillouin zone, weighted by the Fermi-Dirac distribution function,

$$\sigma_{\mathrm{xy}} = -\frac{e^2}{\hbar}\int \frac{d^3k}{(2\pi)^3} \sum_n f(\varepsilon_{nk} - E_F)\Omega_{n,\mathrm{z}}(k). \qquad \text{(S16)}$$

To accurately capture the highly localized Berry-curvature peaks in the 3Q state, the integration is performed using adaptive recursive mesh refinement. The calculated anomalous Hall conductivity as function of Fermi energy is presented in Supplementary Fig. 13b. The magnitude and energy dependence are consistent with experimentally reported low-temperature anomalous Hall responses[8], and thus supporting the validity of the loop-current model adopted here.

Collectively, the emergence of finite Berry curvature and anomalous Hall conductivity provides direct evidence that the loop-current order generates a time-reversal-symmetry-broken electronic state capable of supporting higher-order Berry-curvature multipoles.

Having established the emergence of finite Berry curvature from loop-current order, we next evaluate the higher-order geometric quantities responsible for the third-order nonlinear Hall response. The third-order nonlinear Hall response (3ω) can be intrinsically driven by two distinct geometric mechanisms: the Berry curvature quadrupole (BCQ) and the Berry connection polarizability (BCP). The BCQ describes the second momentum-derivative of the Berry curvature over the equilibrium Fermi distribution and is defined by the rank 3 tensor,

$$Q_{\alpha\beta\gamma} = \int_{\boldsymbol{k}} f_0 \partial_\alpha \partial_\beta \Omega_\gamma. \qquad \text{(S17)}$$

The third order conductivity tensor arising from BCQ scales as,

$$\sigma_{\mu\alpha\beta\gamma}(3\omega) \propto \frac{e^4\tau^2}{4\hbar^3} \epsilon_{\mu\alpha\delta} Q_{\beta\gamma\delta}. \qquad \text{(S18)}$$

Conversely, the BCP contribution arises from the interband coherence and the quantum metric of the Bloch states, captured by the tensor,

$$G_{ab} = 2\mathrm{Re} \sum_{m\neq n} \frac{(A_a)_{nm}(A_b)_{mn}}{\epsilon_n - \epsilon_m}, \qquad \text{(S19)}$$

where m, n are band indices and a, b are cartesian indices. $A_{nn} = \langle u_n|\nabla|u_n\rangle$ is the berry connection and u_n is the eigen state corresponding to energy eigenstate ϵ_n. The resulting third-order susceptibility χ^I_{abcd} involves both Fermi Sea terms (derivatives of G_{ab}) and Fermi surface terms (involving the derivative of the distribution function), as given by[9],

$$\chi^I_{abcd} = \tau \int d\boldsymbol{k}(-\partial_a\partial_b G_{cd} + \partial_a\partial_d G_{bc} - \partial_b\partial_d G_{ac}) f_0 + \frac{\tau}{2}\int d\boldsymbol{k}\, v_a v_b G_{cd} f_0''. \qquad \text{(S20)}$$

Unlike BCQ which scales quadratically with τ^2, the governing equation for the BCP clearly shows an overall linear pre-factor of relaxation time, dictating a τ^1 scaling. This difference in scattering time dependence provides the primary theoretical and experimental basis for distinguishing between the field-induced modifications of the Berry connection (BCP) and the intrinsic higher-order multipoles of the Berry curvature (BCQ) in our experiments. Furthermore, the BCP unlike BCQ is not restricted to time reversal symmetry breaking, and therefore remains finite even in the absence of loop-current order. Supplementary Figure 14 presents the calculated BCP tensor components with and without loop-current order. While the loop currents enhance the overall magnitude of the BCP response, finite BCP contributions already exist in the time-reversal-symmetric state.

Taken together, the calculations reveal a clear distinction between BCQ and BCP. While both quantities contribute to third-order nonlinear transport, only BCQ exhibits a strong sensitivity to loop-current-induced time-reversal-symmetry breaking and follows the experimentally observed quadratic conductivity scaling. These observations identify BCQ as the dominant intrinsic mechanism underlying the third-order nonlinear Hall response in the CDW phase of CsV_3Sb_5.

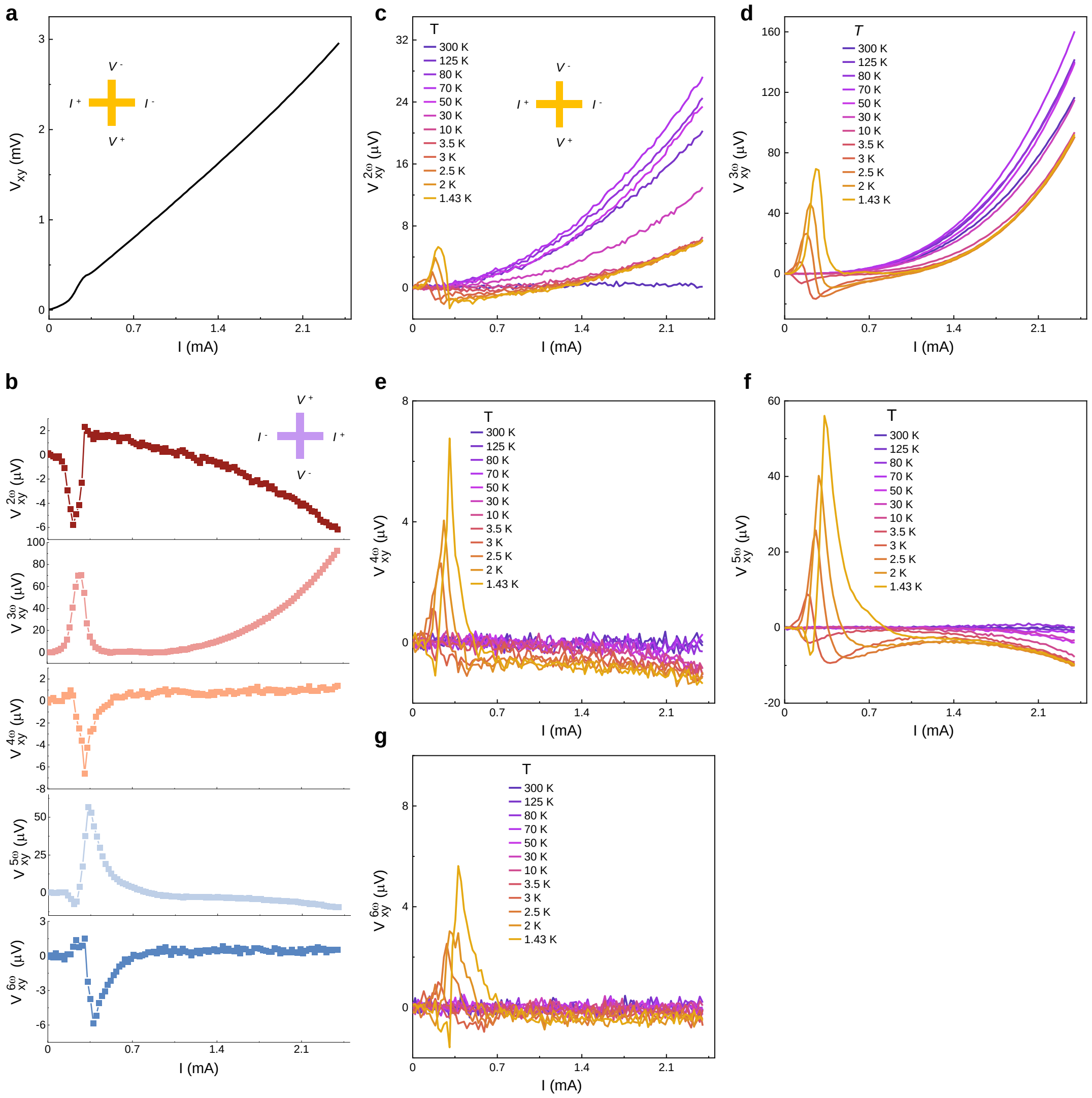

Supplementary Figure 1. Higher-harmonics nonlinear Hall response in the superconducting CsV_3Sb_5. (a) Multiple-order harmonic transverse voltage as a function of alternating current measured at 1.43 K. (b, c, d, e, f) Representative second-order, third-order, forth-order, fifth-order, sixth-order harmonic transverse voltage as a function of alternating current at varying temperatures. In the superconducting state (at 1.43 K), pronounced peaks emerged in the second- to sixth-harmonic transverse voltages ($V_{xy}^{2\omega}$, $V_{xy}^{3\omega}$, $V_{xy}^{4\omega}$, $V_{xy}^{5\omega}$, and $V_{xy}^{6\omega}$) near the superconducting transition. Well above T_c, the peak features vanish. Insets, measurement setups.

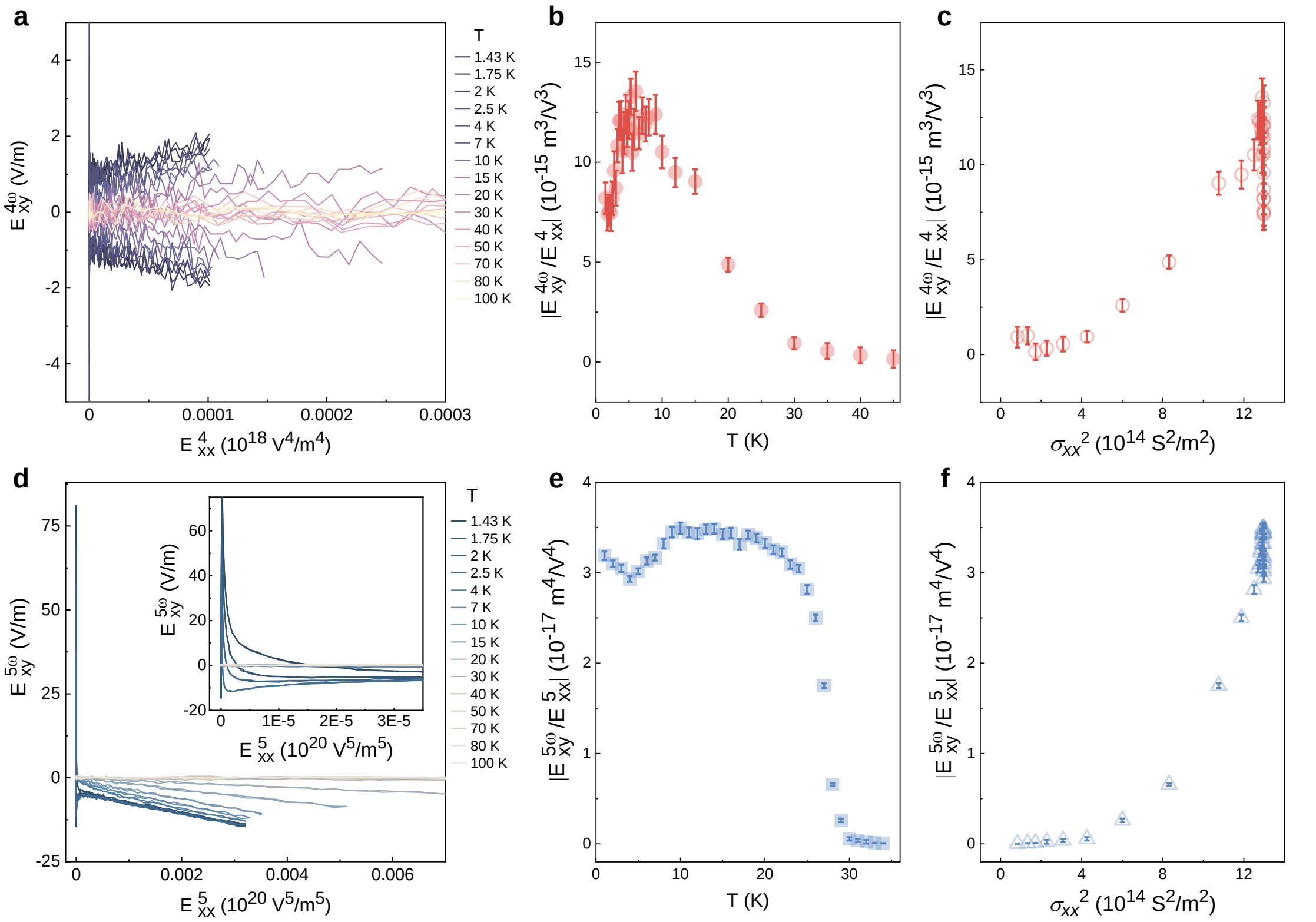

Supplementary Figure 2. Temperature dependent fourth- and fifth-harmonic nonlinear susceptibility in CsV_3Sb_5. (a, d) Linear dependence of $E_{xy}^{4\omega}$ ($E_{xy}^{5\omega}$) on ${E_{xx}}^4$ (${E_{xx}}^5$) ranging from 1.43 K to 100 K for the two opposite measurement configurations with current direction and Hall probes reversed simultaneously. (b, e) Temperature dependence of the corresponding nonlinear susceptibilities $E_{xy}^{4\omega}/{E_{xx}}^4$ and $E_{xy}^{5\omega}/{E_{xx}}^5$, which decrease monotonically with increasing temperature and drop sharply above the CDW transition. (c, f) Nonlinear susceptibility $E_{xy}^{4\omega}/{E_{xx}}^4$ and $E_{xy}^{5\omega}/{E_{xx}}^5$ as a function of the square of conductivity (${\sigma_{xx}}^2$). The error bars in Supplementary Fig. 2b-c and Fig. 2e-f reflect the standard fitting error of $E_{xy}^{4\omega}$ versus ${E_{xx}}^4$ ($E_{xy}^{5\omega}$ versus ${E_{xx}}^5$).

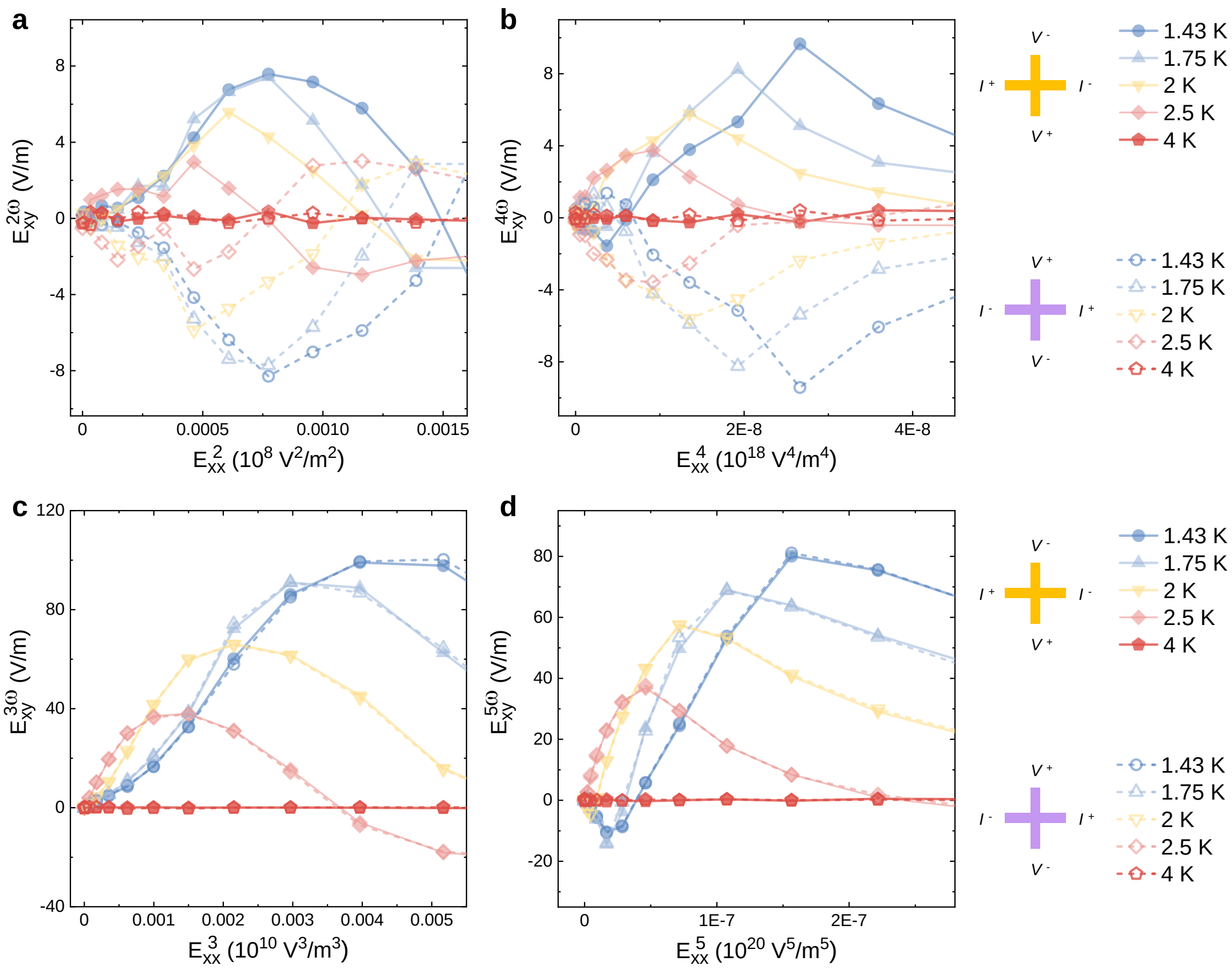

Supplementary Figure 3. (a-d) $E_{xy}^{2\omega}$, $E_{xy}^{3\omega}$, $E_{xy}^{4\omega}$, and $E_{xy}^{5\omega}$ as functions of $E_{xx}{}^2$, $E_{xx}{}^3$, $E_{xx}{}^4$, and $E_{xx}{}^5$ measured in the superconducting state (T<T_c) and below critical current. Solid and dashed lines correspond to the two opposite measurement configurations illustrated in the right panels.

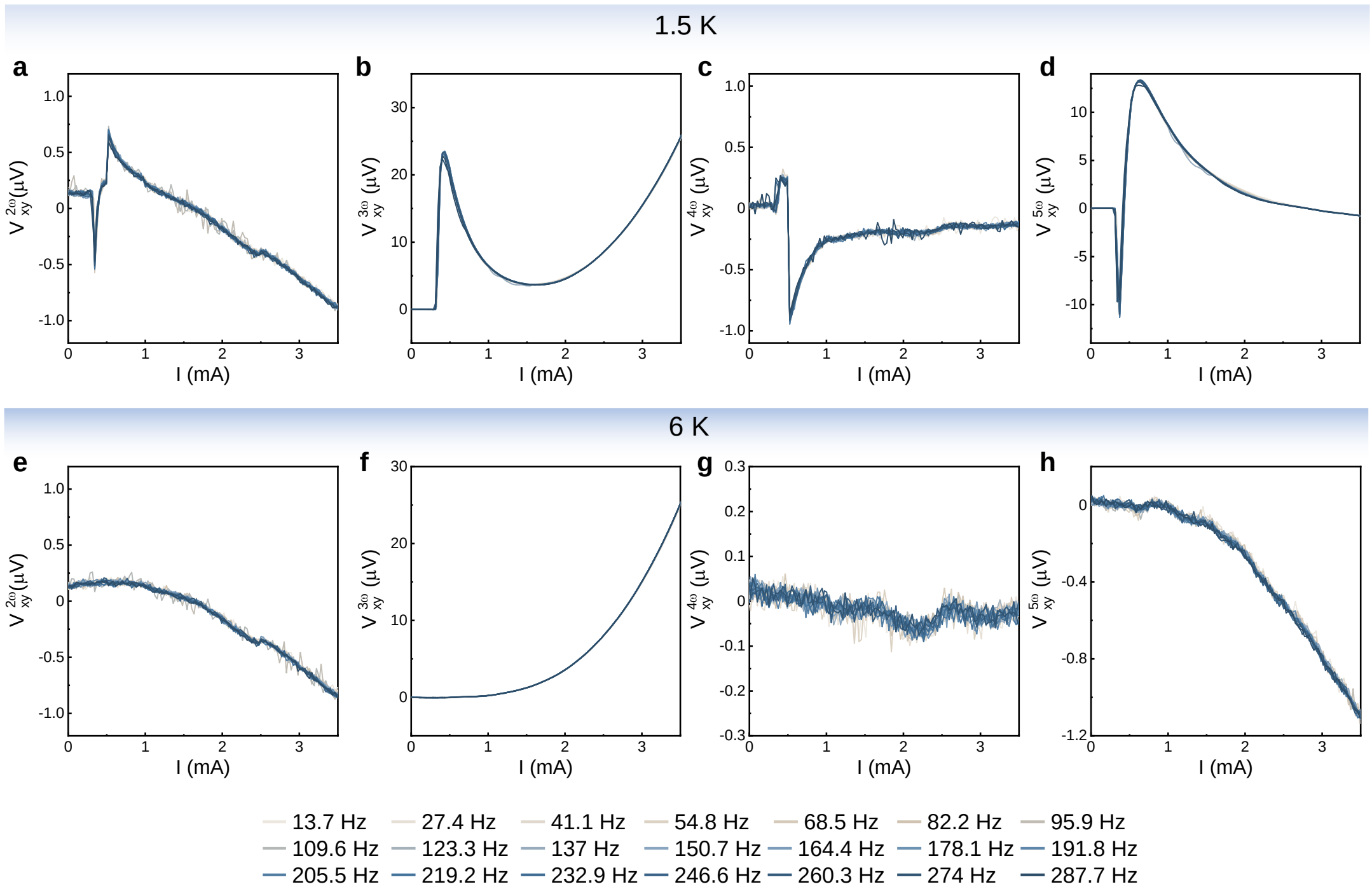

Supplementary Figure 4. Frequency-dependent multiple-harmonic transverse response as a function of current at 1.5 K (a-d) and 6 K (e-h). The overlapped nonlinear responses also help to exclude the thermal and capacitance coupling effects.

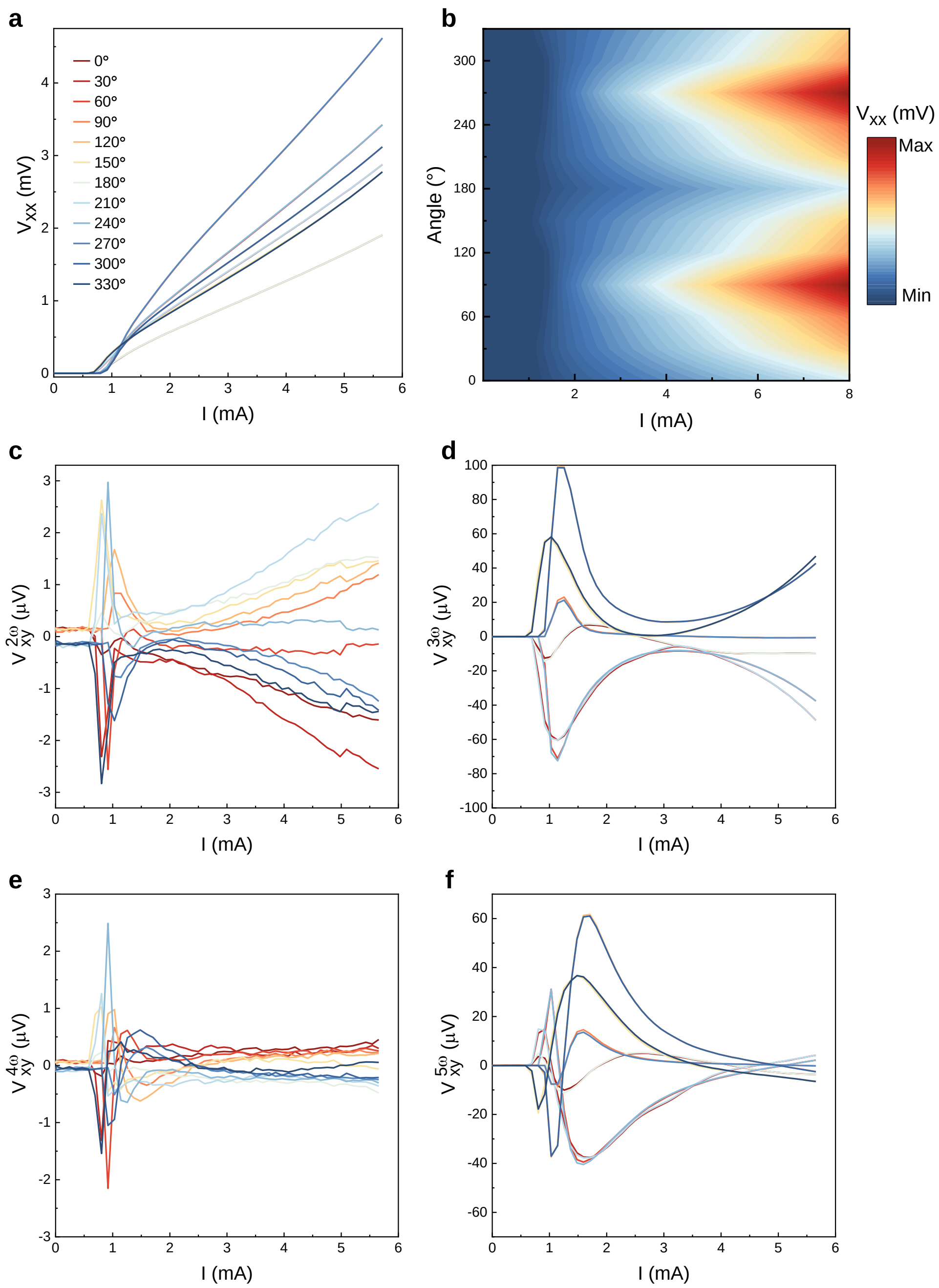

Supplementary Figure 5. Original data of the angle-dependent (a) longitudinal voltage and (b-e) multi-harmonic transverse responses as functions of current for the device shown in Figure 3 at 1.5 K.

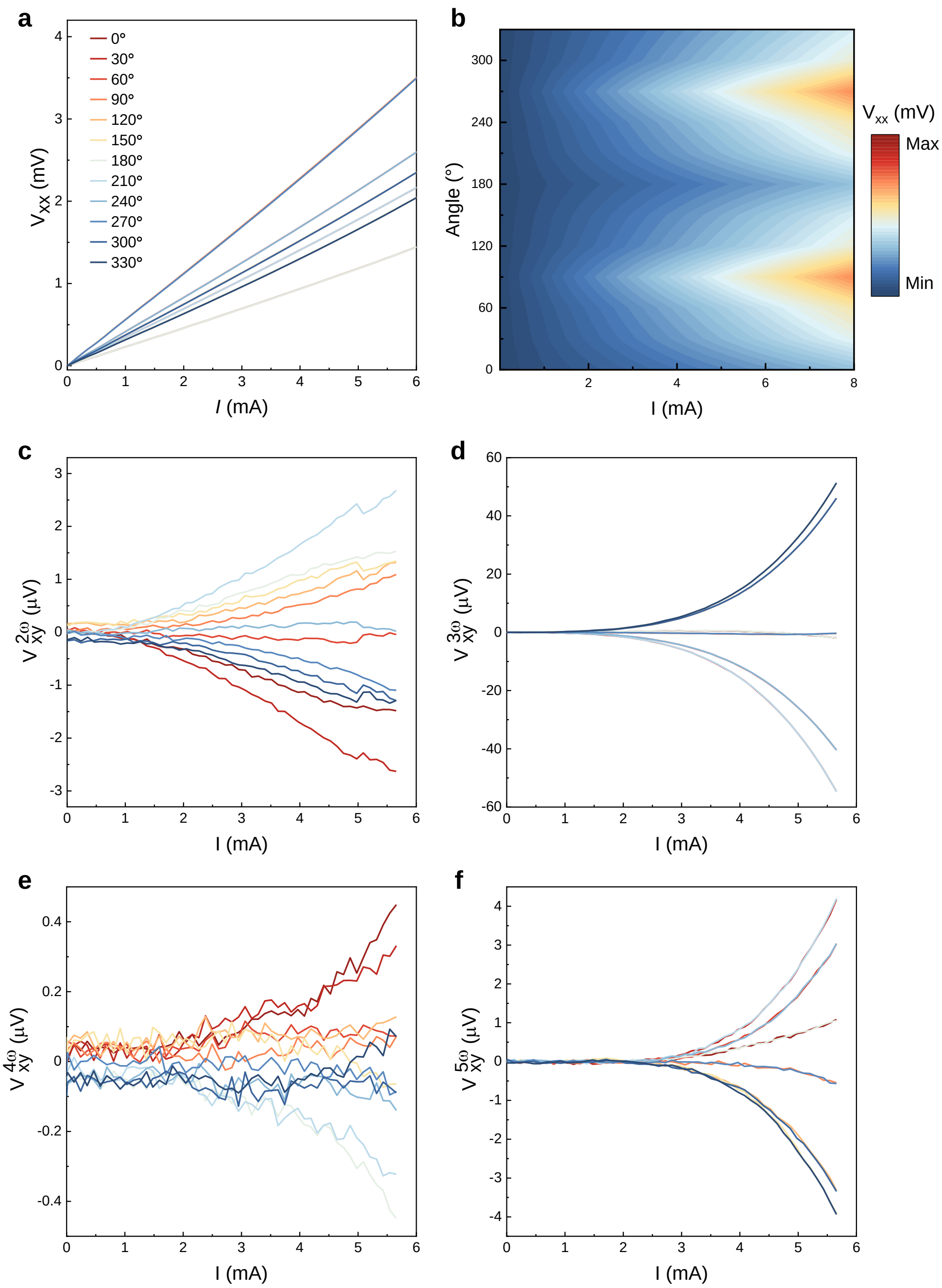

Supplementary Figure 6. Original data of the angle-dependent (a) longitudinal voltage and (b-e) multi-harmonic transverse responses as functions of current for the device shown in Figure 3 at 10 K.

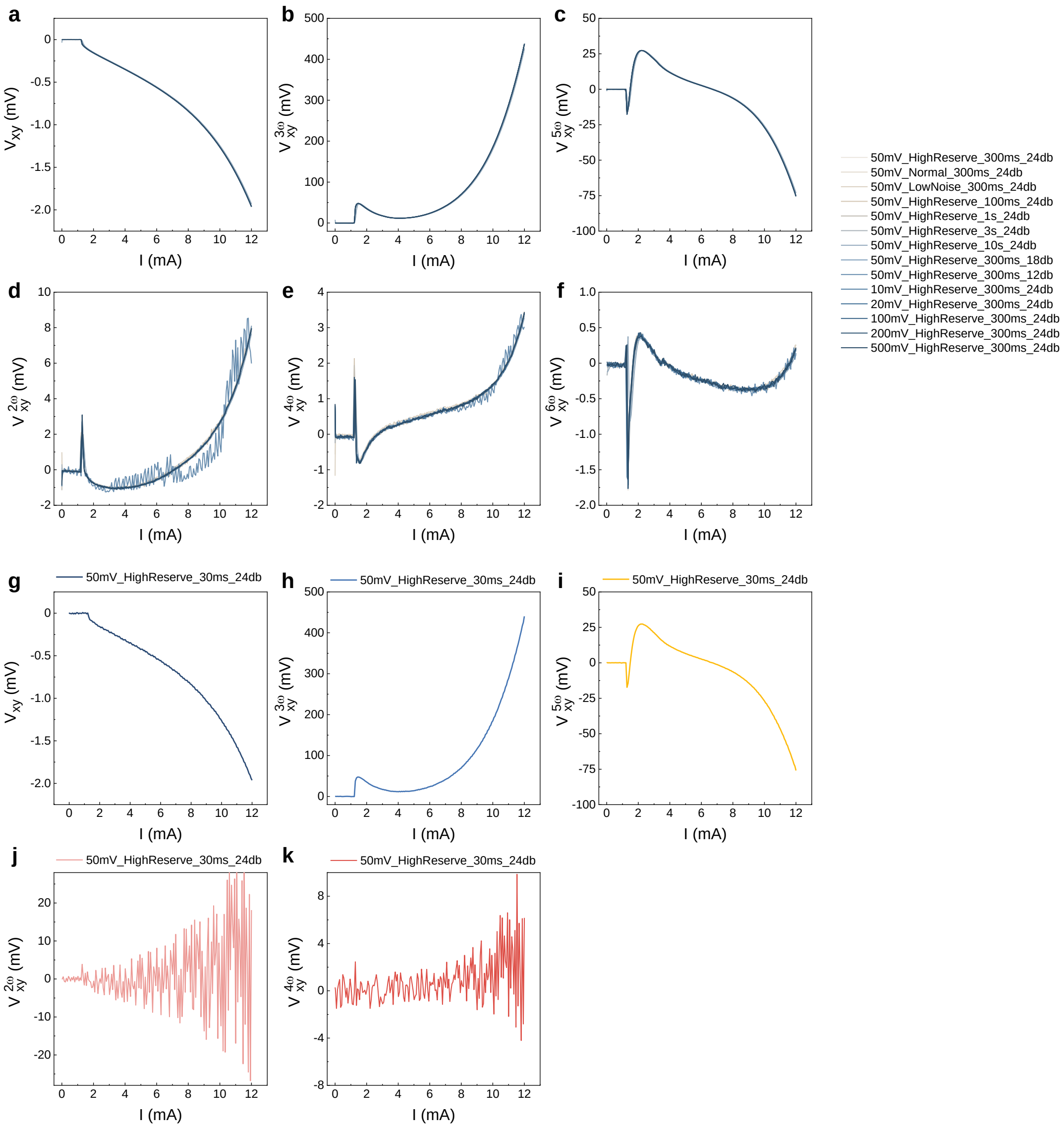

Supplementary Figure 7. Dependence of the nonlinear Hall responses of channel 1-2 on lock-in amplifier settings. (a–f) V_{xy}, $V_{xy}^{2\omega}$, $V_{xy}^{3\omega}$, $V_{xy}^{4\omega}$, $V_{xy}^{5\omega}$ and $V_{xy}^{6\omega}$ measured under different reserve modes (High Reserve, Normal, and Low Noise), sensitivity ranges (10–500 mV), time constants (30 ms–10 s), and filter slopes (12–24 dB/oct). (g–k) V_{xy}, $V_{xy}^{2\omega}$, $V_{xy}^{3\omega}$, $V_{xy}^{4\omega}$ and $V_{xy}^{5\omega}$ measured at time constants of 30 ms. No systematic changes in the peak position or overall signal evolution are observed upon varying the lock-in settings.

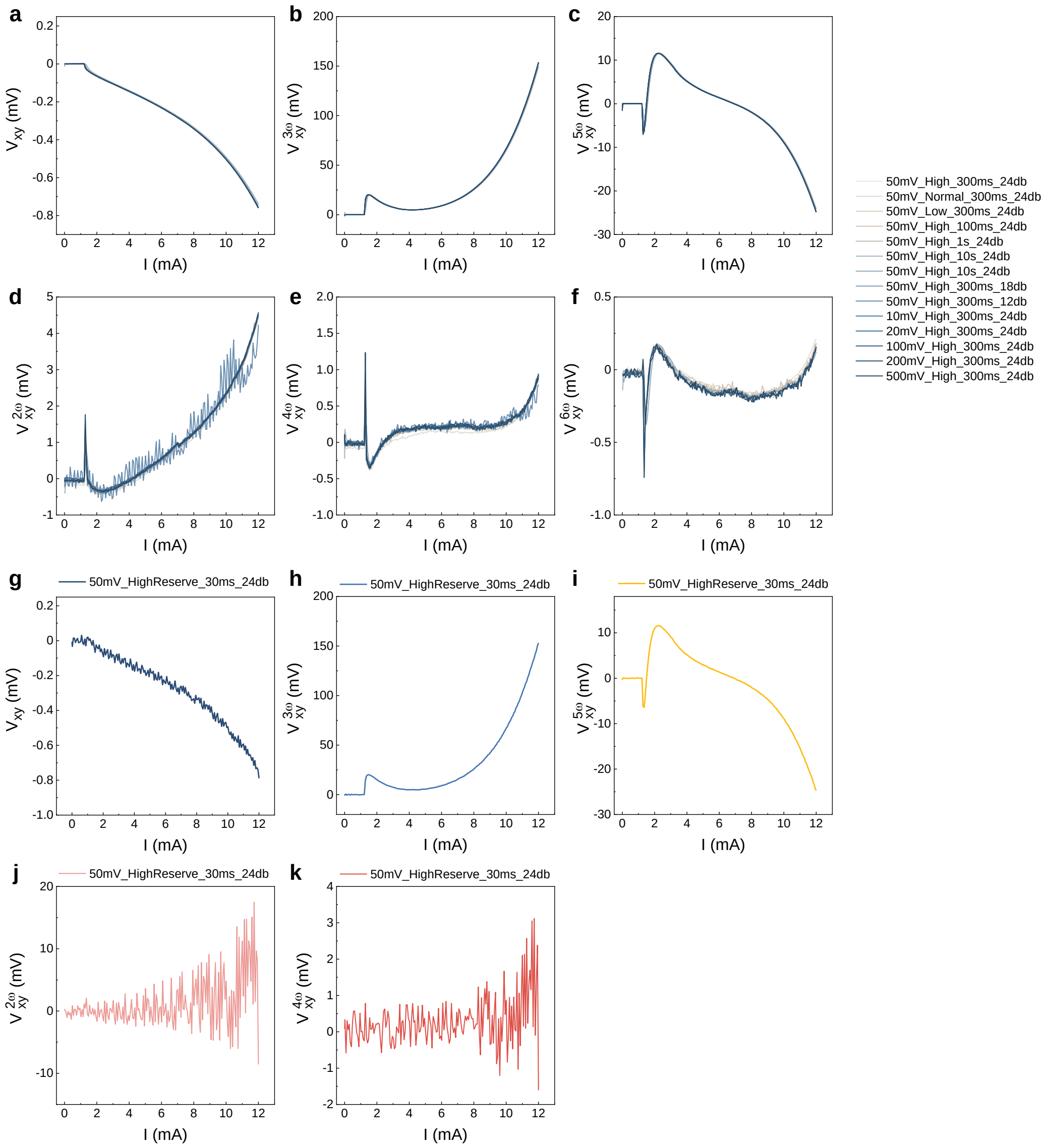

Supplementary Figure 8. Dependence of the nonlinear Hall responses of channel 3-4 on lock-in amplifier settings. (a–f) V_{xy}, $V_{xy}^{2\omega}$, $V_{xy}^{3\omega}$, $V_{xy}^{4\omega}$, $V_{xy}^{5\omega}$ and $V_{xy}^{6\omega}$ measured under different reserve modes (High Reserve, Normal, and Low Noise), sensitivity ranges (10–500 mV), time constants (30 ms–10 s), and filter slopes (12–24 dB/oct). (g–k) V_{xy}, $V_{xy}^{2\omega}$, $V_{xy}^{3\omega}$, $V_{xy}^{4\omega}$ and $V_{xy}^{5\omega}$ measured at time constants of 30 ms. No systematic changes in the peak position or overall signal evolution are observed upon varying the lock-in settings.

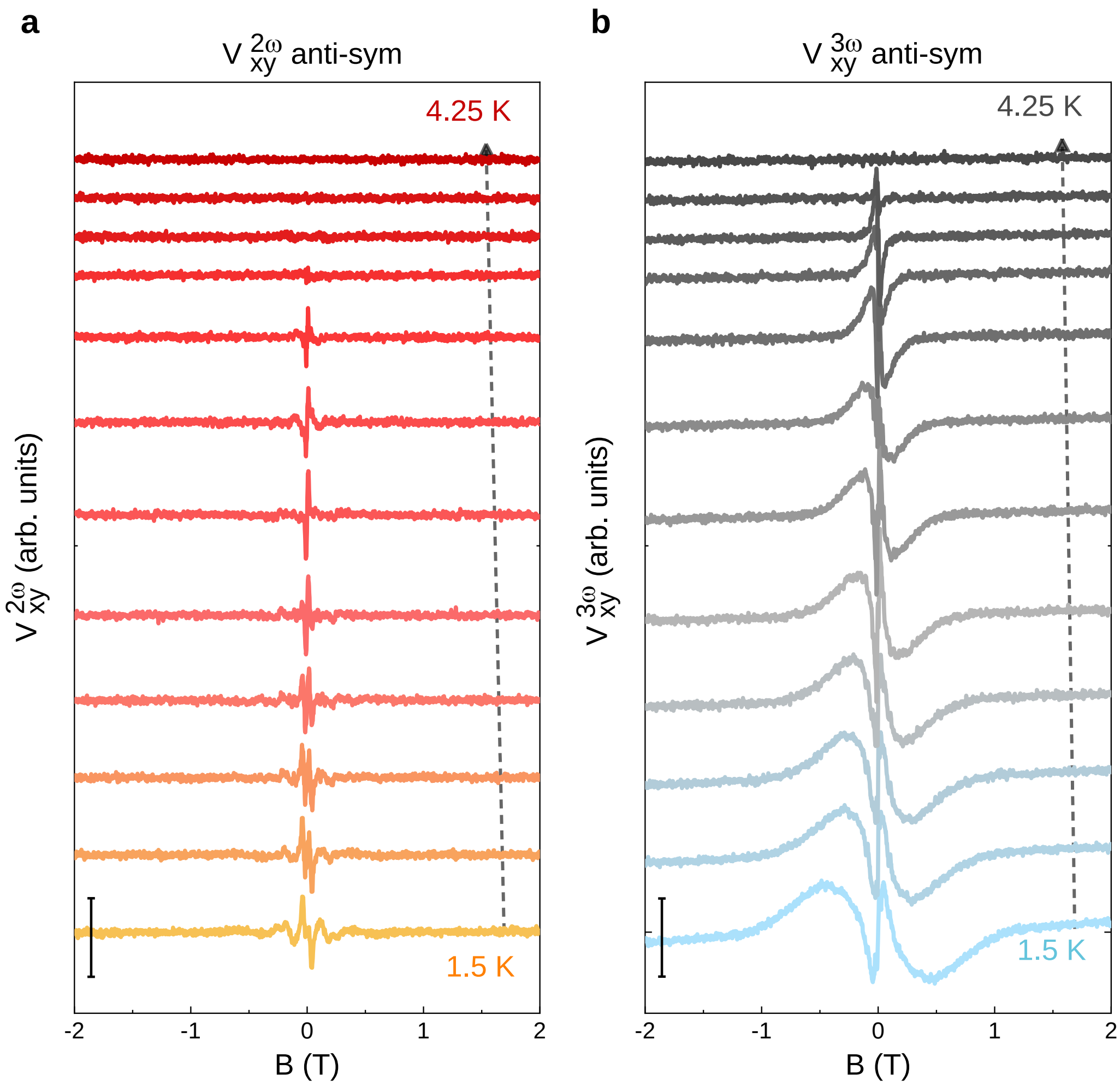

Supplementary Figure 9. Full magnetic-field range data of the anti-symmetric $V_{xy}^{2\omega}$ and $V_{xy}^{3\omega}$ for the device shown in Figure 5 in the main text. Scale bar: 1 μV.

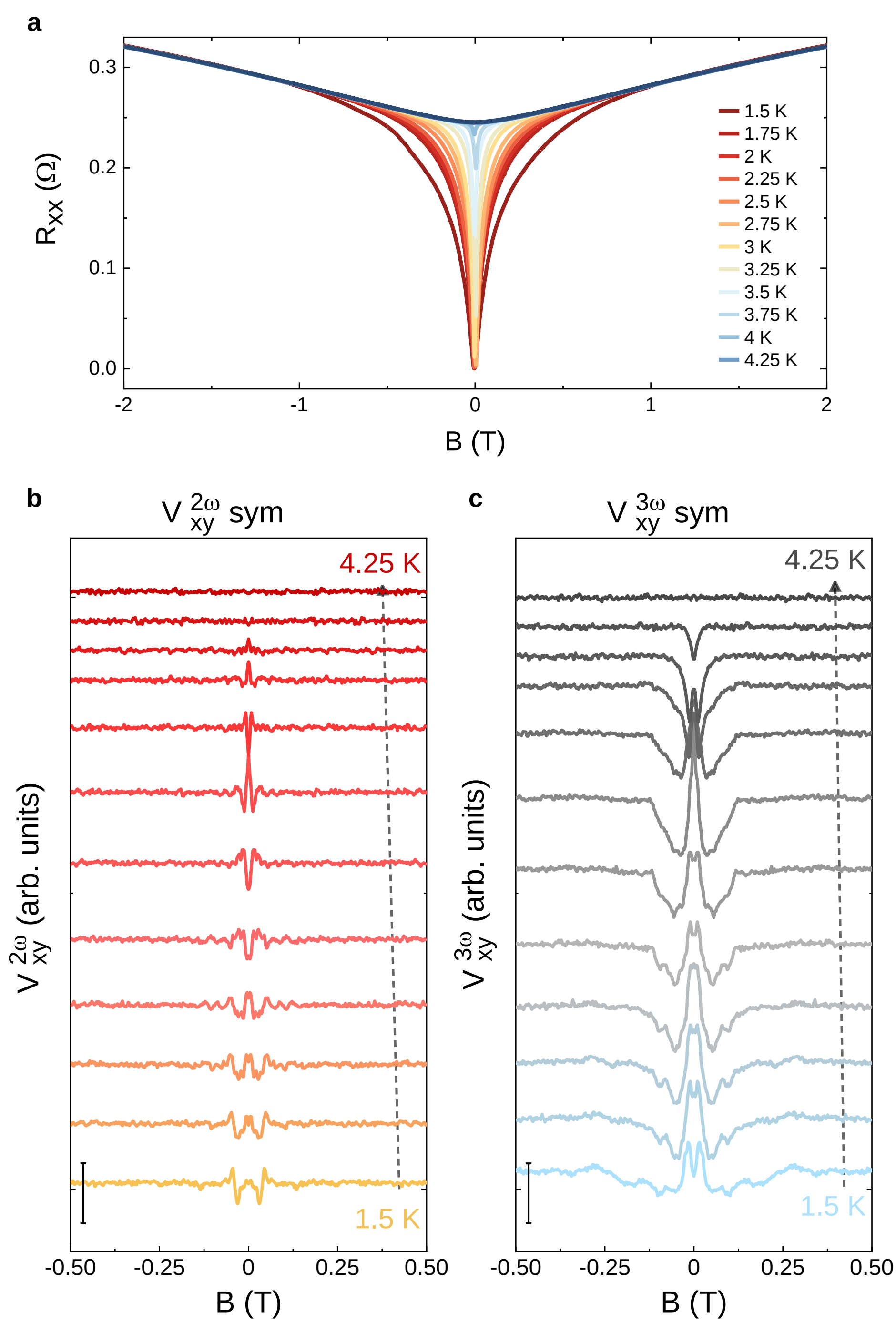

Supplementary Figure 10. Additional transport data of device in Figure 5 in the main text. (a) Longitudinal magnetoresistance R_{xx} at varying temperatures. The applied ac current is 0.5 mA. (b, c) Temperature-dependent symmetric part of $V_{xy}^{2\omega}$ and $V_{xy}^{3\omega}$ as a function of out-of-plane field. Scale bar: 1 μV.

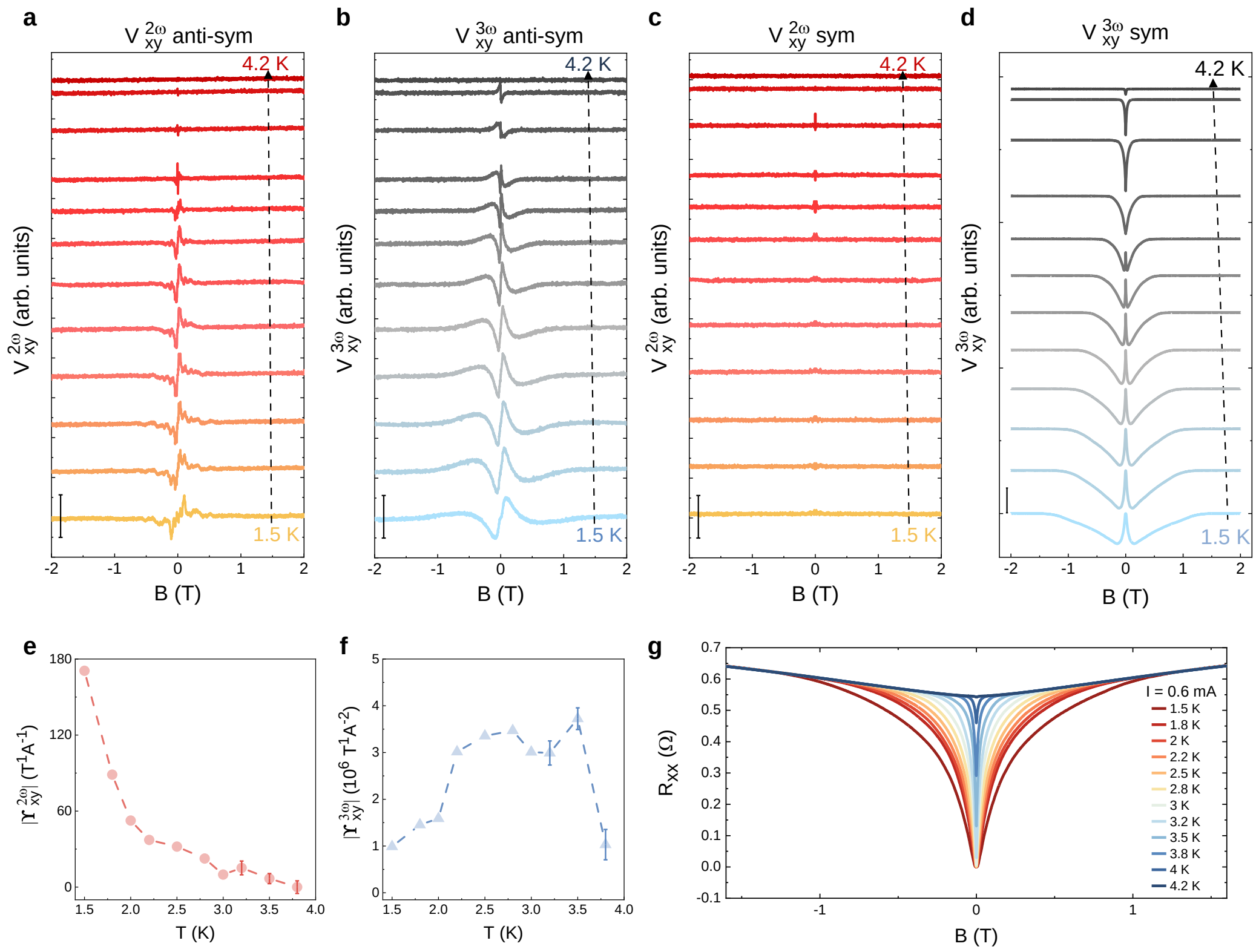

Supplementary Figure 11. Nonreciprocal transverse transport in additional CsV_3Sb_5 device. (a, c) Anti-symmetric and symmetric part of $V_{xy}^{2\omega}$ as a function of out-of-plane magnetic field. Scale bar: 1 μV. (b, d) Anti-symmetric and symmetric part of $V_{xy}^{3\omega}$ as a function of out-of-plane magnetic field. Scale bar in c: 1 μV. Scale bar in d: 20 μV. (e, f) Extracted $\gamma_{xy}^{2\omega}$ and $\gamma_{xy}^{3\omega}$ as a function of temperature. The error bars represent the uncertainty in determining the field B and R_{xx} from (a, b) due to the gradual transition. (g) Longitudinal magnetoresistance R_{xx} at varying temperatures.

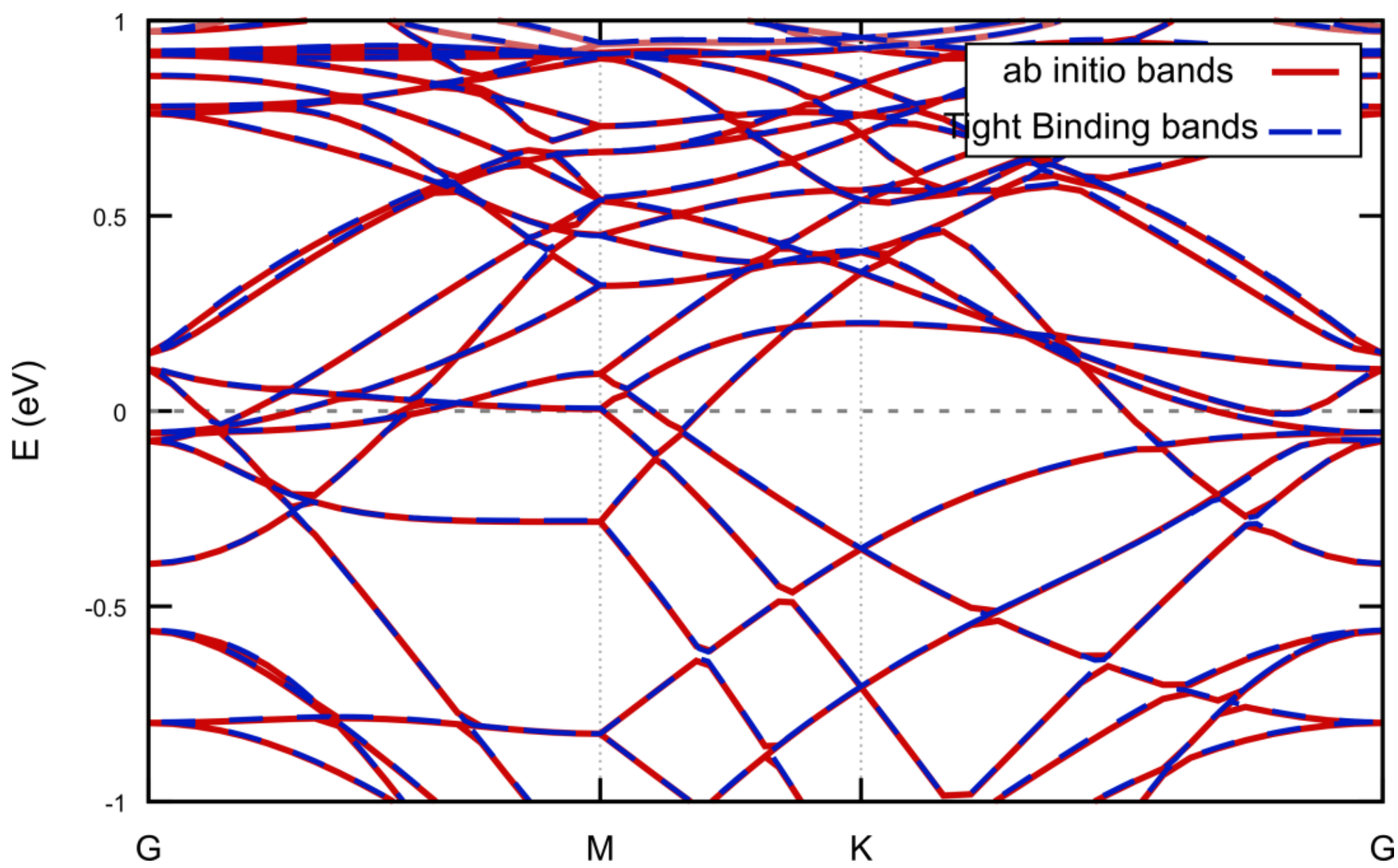

Supplementary Figure 12. Wannier extrapolated band structure. The red solid lines are ab initio electronic band structure for 2x2x4 supercell with CDW modulations, and the blue dashed lines represent the Wannier extrapolated band structure.

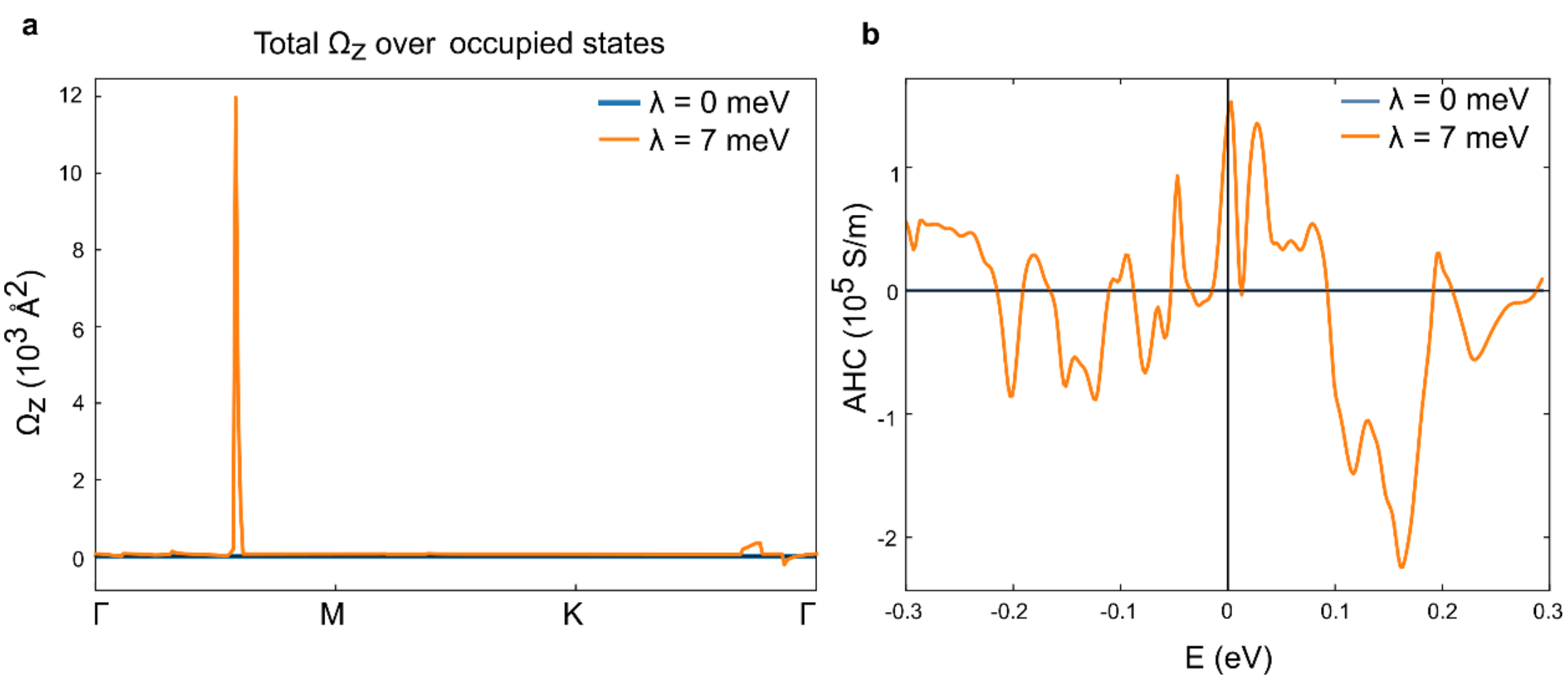

Supplementary Figure 13. Loop-current-induced Berry curvature and Anomalous Hall conductivity in the CDW phase of CsV_3Sb_5. (a) Out-of-plane berry curvature Ω_z along the high-symmetry directions of the Brillouin zone calculated with ($\lambda = 7$ meV) and without ($\lambda = 0$ meV) loop-current order. (b) Intrinsic anomalous Hall conductivity as a function of the Fermi energy.

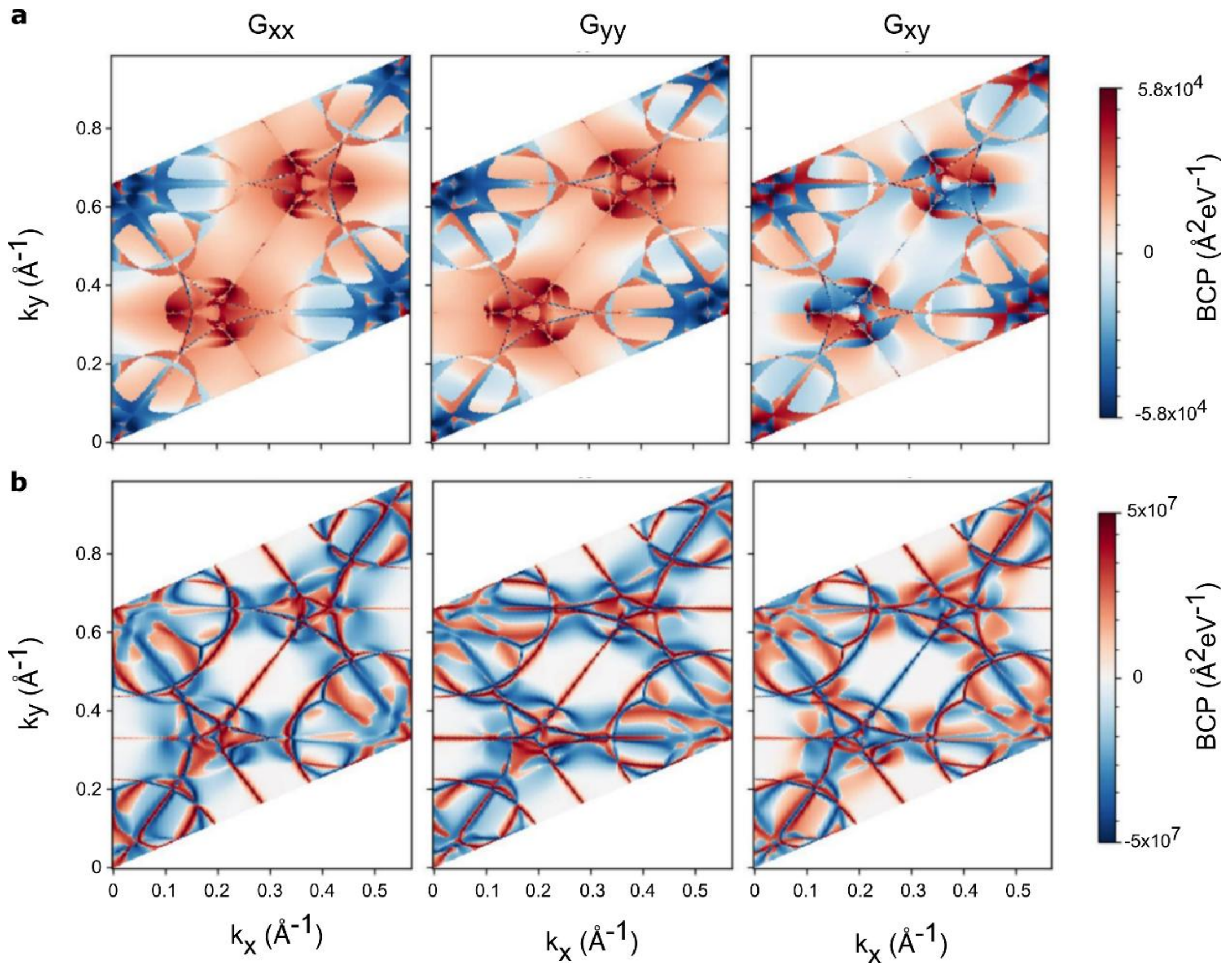

Supplementary Figure 14. Berry connection polarizability (BCP) in CsV_3Sb_5. (a) BCP tensor components ($\mathbb{G}_{ij}$) in the absence of loop currents ($\lambda = 0$ meV). (b) BCP tensor components in the presence of the loop-current order ($\lambda = 7$ meV). Unlike the BCQ, the BCP remains finite even without time-reversal-symmetry breaking, although its magnitude is enhanced by the loop-current order.

References

1. Mostofi, A. A. *et al.* wannier90: A tool for obtaining maximally-localised Wannier functions. *Comput. Phys. Commun.* **178**, 685–699 (2008).
2. Kautzsch, L. *et al.* Structural evolution of the kagome superconductors AV3Sb5 (A = K, Rb, and Cs) through charge density wave order. *Phys. Rev. Mater.* **7**, 024806 (2023).
3. Guo, C. *et al.* Correlated order at the tipping point in the kagome metal CsV3Sb5. *Nat. Phys.* **20**, 579–584 (2024).
4. Tazai, R., Yamakawa, Y. & Kontani, H. Charge-loop current order and Z3 nematicity mediated by bond order fluctuations in kagome metals. *Nat. Commun.* **14**, (2023).
5. Feng, X., Jiang, K., Wang, Z. & Hu, J. Chiral flux phase in the Kagome superconductor AV3Sb5. *Sci. Bull.* **66**, 1384–1388 (2021).
6. Li, H. *et al.* Small Fermi Pockets Intertwined with Charge Stripes and Pair Density Wave Order in a Kagome Superconductor. *Phys. Rev. X* **13**, 031030 (2023).
7. Wang, Z. *et al.* Distinctive momentum dependent charge-density-wave gap observed in CsV3Sb5 superconductor with topological Kagome lattice. Preprint at https://doi.org/10.48550/arXiv.2104.05556 (2021).

8. Zheng, G. *et al.* Electrically controlled superconductor-to-failed insulator transition and giant anomalous Hall effect in kagome metal CsV3Sb5 nanoflakes. *Nat. Commun.* **14**, (2023).
9. Liu, H. *et al.* Berry connection polarizability tensor and third-order Hall effect. *Phys. Rev. B* **105**, 045118 (2022).
