# Supplementary material for: Berry curvature multipoles in Kagome superconductor CsV3Sb5
Source: Nat Commun. 2026 Sep 10;17:10683. doi: 10.1038/s41467-026-77618-6 (PMC13654926; doi:10.1038/s41467-026-77618-6)
Supplement: Supplementary file 2 [file 41467_2026_77618_MOESM2_ESM.pdf]

nature portfolio

Peer Review File

Berry Curvature Multipoles in Kagome Superconductor CsV3Sb5

Corresponding Author: Professor Kian Ping Loh

This file contains all reviewer reports in order by version, followed by all author rebuttals in order by version.

Version 0:

Reviewer comments:

Reviewer #1

(Remarks to the Author)
In this manuscript, the authors report high-order nonlinear Hall measurements (up to the sixth harmonic) in the Kagome superconductor CsV3Sb5. They perform systematically measurement and argue that the third-order nonlinear Hall response, which is associated with the Berry curvature quadrupole (BCQ), exhibits a pronounced enhancement near the superconducting transition due to vortex dynamics. Consequently, the authors propose that the BCQ serves as a sensitive probe for electronic nematicity in Kagome superconductors. While this work is experimentally rich and timely, several conceptual and interpretational issues must be addressed first.

1.The authors state that the current I was applied 'along the crystal axis'. However, they do not specify which axis was used. It would also be valuable to know if the signal would vary depending on the different axes.

2.When the applied current is larger than the critical current, does the device transit to CDW state or some other state?

3.Regarding Fig 2 f, g, the authors claim that all transverse voltages exhibit clear quadratic, cubic and higher-order dependence on the applied current. However, this is not clear to me. Could the authors provide the corresponding fitting curves? Furthermore, please clarify whether this specific nonlinear current dependence holds consistently across both the superconducting (SC) state and the state above the critical current.

4.When applying the out-of-plane magnetic field, is it possible the anti-symmetric part is caused by Berry connection polarizability dipole or called quantum metric dipole?

5.In Fig S1a, why does Vxy not show superconducting responses at low current? For comparison, in Fig S4a, Vxx shows superconducting responses when the current is below around 0.6 mA.

Minor point: In Fig S8, (d, e) Anti-symmetric and symmetric part of …. This appears to be a typo and should be corrected to (b, d).

Reviewer #2

(Remarks to the Author)
The manuscript reports the experimental measurements of high-order nonlinear Hall effect up to the sixth harmonic in Kagome superconductor CsV3Sb5. The vortex dynamics near the superconducting transition of CsV3Sb5 are probed through third-order nonlinear Hall effect with assistance of Berry curvature quadrupoles effect. This work paves a way to explore subtle quantum phase transition using the nonlinear transport techniques, which could be greatly advance the relevant research community. These observations are noteworthy, but the analyses are lacking rigorous. I will recommend its publication only after the following issues have been well addressed.

1.In Figure 2, the authors attribute the enhancement of nonlinear transverse voltages near 70 K to the formation of charge

density wave (CDW) order. While the second-harmonic response exhibits a significant enhancement at temperature closes to the CDW transition, the third-harmonic response shows a pronounced enhancement at temperatures substantially below the CDW transition. I recommend the authors explain the effect of CDW order on the nonlinear response in different harmonics and provide more experimental evidence or theoretical analysis to substantiate this interpretation.
2.Figure 2b appears to present two sets of second-harmonic Hall responses with opposite sign reversals. Could the author clarify the origin of these results and identify the mechanism responsible for the sign reversal?
3.The emergence of the peak in the I-V measurements at relatively small a.c. current may be influenced by the lock-in amplifier, for instance, changes in sensitivity range or input dynamics. In addition, the peak position can also be varied as the series resistance in the measurement circuit changes. The correlation between the peak evolution with the superconducting transition lacks robust experimental and theoretical support. The authors should provide control experiments or analyses to rule out measurement artifacts and to firmly establish the relationship with superconductivity.
4.As shown in Figure S1a, the superconducting state is suppressed as excitation current exceeds ~0.5 mA. However, the nonlinear responses at different harmonics are obtained in both superconducting and normal states. Could the author identify the distinct physical origins of the nonlinear responses in these two phases?
5.How the authors exclude the thermal effects from the measured nonlinear responses as the excitation current is as large as 3 mA, such as such as Joule heating or thermoelectric voltages?

Reviewer #3

(Remarks to the Author)
I co-reviewed this manuscript with one of the reviewers who provided the listed reports. This is part of the Nature Communications initiative to facilitate training in peer review and to provide appropriate recognition for Early Career Researchers who co-review manuscripts.

Version 1:

Reviewer comments:

Reviewer #1

(Remarks to the Author)
I have reviewed the revised manuscript and the authors' responses to my previous comments. I am satisfied with the revisions and believe that the authors have addressed the concerns in a reasonable and well-supported manner. The manuscript has been improved substantially, and I support its publication in Nature Communications.

Reviewer #2

(Remarks to the Author)
The authors have made substantial revisions to the manuscript and addressed all my concerns. I therefore support the publication of the revised manuscript in Nature Communications. The only minor issue is a typo on page 4 of the supplementary information, where "The third-harmonic transverse signal" should be changed to "The fourth-harmonic transverse signal".

Reviewer #3

(Remarks to the Author)
I co-reviewed this manuscript with one of the reviewers who provided the listed reports. This is part of the Nature Communications initiative to facilitate training in peer review and to provide appropriate recognition for Early Career Researchers who co-review manuscripts.

Open Access This Peer Review File is licensed under a Creative Commons Attribution 4.0 International License, which permits use, sharing, adaptation, distribution and reproduction in any medium or format, as long as you give appropriate credit to the original author(s) and the source, provide a link to the Creative Commons license, and indicate if changes were made.
In cases where reviewers are anonymous, credit should be given to 'Anonymous Referee' and the source.
The images or other third party material in this Peer Review File are included in the article’s Creative Commons license, unless indicated otherwise in a credit line to the material. If material is not included in the article’s Creative Commons license and your intended use is not permitted by statutory regulation or exceeds the permitted use, you will need to obtain permission directly from the copyright holder.
To view a copy of this license, visit https://creativecommons.org/licenses/by/4.0/

Response to Reviewers' Comments

We thank the Reviewers for their careful reading of our manuscript entitled *"Berry Curvature Multipoles in Kagome Superconductor CsV_3Sb_5"* and for their constructive and insightful comments, which have helped to improve the quality of this work. In response to their suggestions, we have carefully revised the manuscript and improved its presentation. In addition, we have performed new experiments to address all of the reviewers' concerns. All revisions have been made accordingly, and detailed responses to each comment are provided below.

Response to Reviewer #1

General comment:

In this manuscript, the authors report high-order nonlinear Hall measurements (up to the sixth harmonic) in the Kagome superconductor CsV_3Sb_5. They perform systematically measurement and argue that the third-order nonlinear Hall response, which is associated with the Berry curvature quadrupole (BCQ), exhibits a pronounced enhancement near the superconducting transition due to vortex dynamics. Consequently, the authors propose that the BCQ serves as a sensitive probe for electronic nematicity in Kagome superconductors. While this work is experimentally rich and timely, several conceptual and interpretational issues must be addressed first.

Response:

We sincerely thank the Reviewer for the thorough and highly positive evaluation of our manuscript. We greatly appreciate the Reviewer's recognition of the timeliness of this work and the comprehensive nature of our experimental investigations. Please see below for the detailed, point-by-point responses to each of the technical comments.

Comment 1:

The authors state that the current I was applied 'along the crystal axis'. However, they do not specify which axis was used. It would also be valuable to know if the signal would vary depending on the different axes.

Response:

We thank the reviewer for this insightful point, and we have added the x-axis notification in the inset of Fig. 1b and Fig. 3b in the main text. The x-axis aligns with the crystallographic a-axis, which is directed along one of the primary vanadium-vanadium bond directions within the Kagome plane. Indeed, the nonlinear response is axes-dependent, and as shown in Figs. 3b and 3e in the main text (redrawn in Fig. R1), we fabricated disk-shaped device to perform the angular dependent nonlinear measurement. We observed a pronounced two-fold angular symmetry in the odd-order transverse signals, while the even-order transverse components exhibit a nearly one-fold response in both the superconducting and charge-density-wave states. This distinct symmetry dichotomy reflects a spontaneous reduction of the underlying rotational

symmetry and is understood in terms of electronic nematicity emerging from the Kagome lattice. In particular, the two-fold symmetry of the odd-order nonlinear Hall response indicates a nematic electronic state that breaks the native threefold (or sixfold) rotational symmetry, consistent with previous transport and spectroscopic studies reporting C_2 symmetry in CsV_3Sb_5.[1,2]

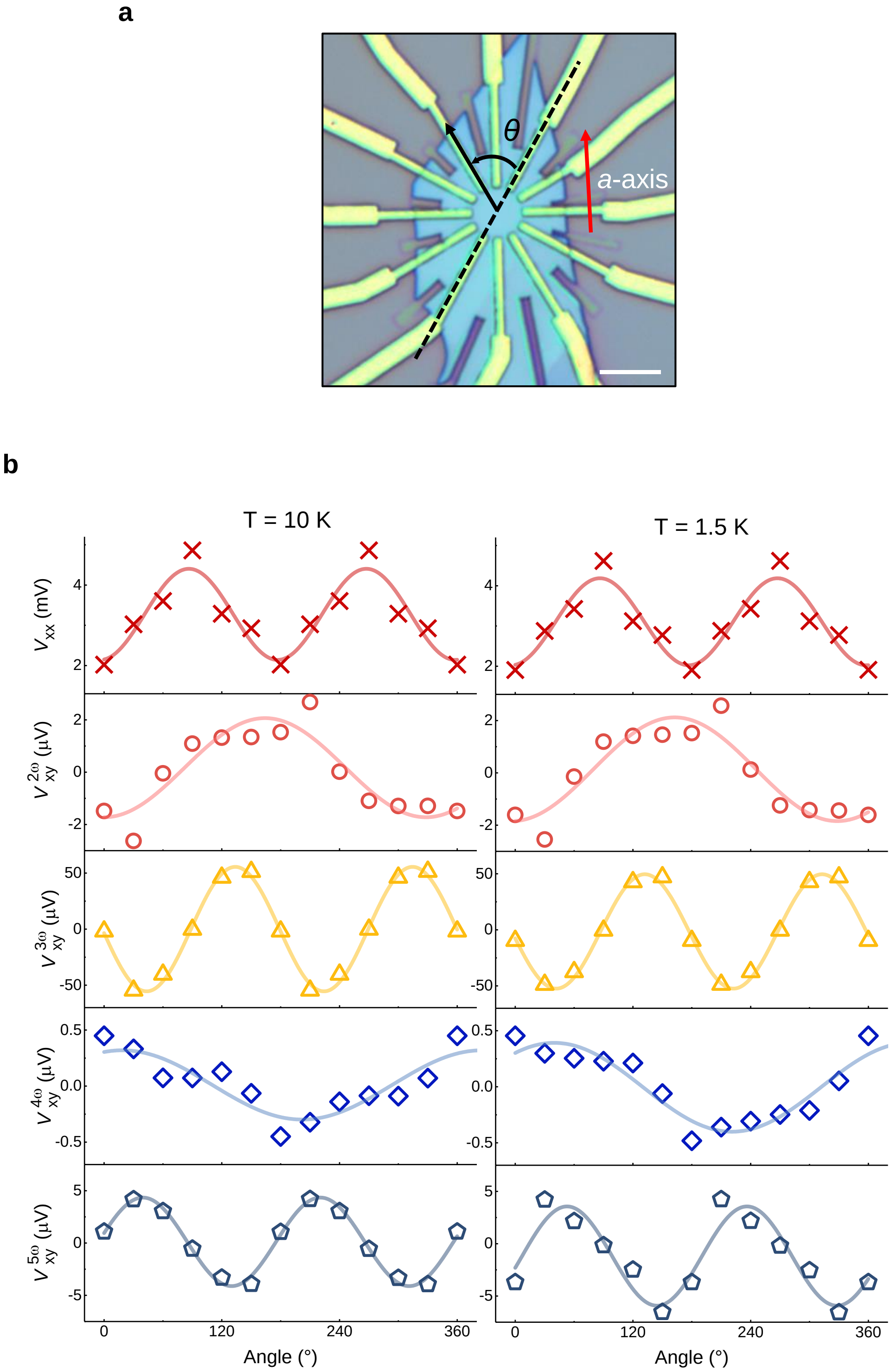

Figure R1. Angular dependence of the nonlinear transport in CsV_3Sb_5 device. (a) Optical image of disk-like device. Scale bar: 5 μm. (b) Angular dependence of the first-

order longitudinal resistance (R_{xx}) and second-, third-, fourth-, and fifth-harmonic transverse voltages measured at 1.5 K and 10 K, respectively. The solid lines are fits to sinusoidal functions with one-fold (360° period) and two-fold (180° period) angular symmetries. Odd-order transverse components exhibit a pronounced two-fold angular symmetry, while even-order transverse components show a nearly one-fold response in both the superconducting and CDW states.

Comment 2:

When the applied current is larger than the critical current, does the device transit to CDW state or some other state?

Response:

We thank the reviewer for this insightful question. When the excitation current surpasses the critical current, the device does not transition into a new phase. The reasons are explained below:

1. **Coexistence of Phases:** The CDW order in this system is established at a much higher temperature ($T \approx$ 75 K) than the superconducting transition temperature ($T_C \approx$ 4.2 K). Experimental evidence, such as x-ray diffraction and spectroscopic imaging scanning tunneling microscopy[3,4], confirms that the CDW phase coexists with the superconducting phase below T_C.
2. **Effect of Increasing Current:** As the current increases beyond the critical threshold, the primary effect is the suppression of the superconducting zero-resistance state. This suppression leads to a finite resistance, driven by phenomena such as vortex depinning, flux flow, and pair breaking[5–7]. Importantly, this does not mean the system transforms into a different phase; rather, the superconductivity is being suppressed within the existing phase.
3. **Persistence of CDW Order:** Even at high currents, the underlying CDW order remains intact. This is evidenced by the persistence of nonlinear Hall responses, which are sensitive to the chiral flux phases associated with the CDW. If the high current were to destroy the CDW order, these nonlinear signals would diminish or vanish. Instead, they persist, indicating the system remains within the CDW phase.
4. **Role of Magnetic Fields and Vortex Motion:** Vortex motion, which can be driven by the magnetic field or high current, contributes to the finite resistance but does not induce a phase transition to another state. The system remains in the CDW phase, just with suppressed superconductivity.

Microscopic Insight into the CDW State:

To better understand the microscopic origin, we have performed first-principles calculations on the complex CDW phase of CsV_3Sb_5. The CDW is characterized by a 3Q order, with real (rCDW) and imaginary (iCDW) components[8,9]. The imaginary part is associated with chiral flux generated by loop currents that break time-reversal symmetry.

Starting from the experimentally determined CDW supercell structure reported by X-ray diffraction studies[8], we modelled these loop currents within a Wannier-based tight-binding framework, incorporating phase factors to emulate maximum time-reversal symmetry breaking. Our calculations show that the presence of loop currents

(λ ≈ 7 meV) reconstructs the electronic band structure, lifts degeneracies near the Fermi level, and generates a finite out-of-plane Berry curvature—a key factor in the observed nonlinear Hall response.

A schematic illustration of the 3Q charge density wave (CDW) ordered state with a chiral flux phase is shown in Fig. R2a. The calculated electronic band structures along the high-symmetry Γ–M direction are presented in Fig. R2b. When the loop-current order parameter λ is set to zero (λ = 0 meV, without loop-current order), the band structure exhibits degeneracies near the Fermi level. However, upon introducing a finite loop-current order (λ = 7 meV), the low-energy electronic structure undergoes significant reconstruction; this breaks time-reversal symmetry and lifts these degeneracies. In Fig. R2b, the band structure for λ = 0 meV is shown as black solid lines, while the λ = 7 meV case is represented by red dashed lines, clearly illustrating the degeneracy breaking caused by the loop currents. More importantly, a finite out-of-plane Berry curvature component (Ω_z) appears only when loop-current order is present, as shown in Figs. R2c-d. For λ = 0 meV, the Berry curvature remains symmetry-forbidden, but with λ = 7 meV, it emerges prominently, indicating that the imaginary part of the complex CDW order—linked to the chiral flux—directly contributes to the Berry curvature.

Building on this microscopic understanding, we evaluate the band-geometric quantities relevant to nonlinear Hall transport. The Berry curvature quadrupole (BCQ), a rank-3 tensor within the 2D Kagome plane, has four non-zero components: Q_{xxz}, Q_{yyz}, Q_{xyz} and Q_{yxz}. The Fermi-energy dependence of these components is shown in Figs. R2e-f. When there is no loop-current order (λ = 0 meV), all BCQ components remain nearly zero across the examined energy range. In stark contrast, introducing loop currents (λ = 7 meV) results in pronounced peaks near the Fermi energy and enhances both the diagonal components (Q_{xxz} and Q_{yyz}) and the off-diagonal components (Q_{xyz} and Q_{yxz}) by several orders of magnitude. The differing signs and magnitudes of the diagonal components reflect the broken nematicity phase, while the off-diagonal components, which are equal due to the symmetry of partial derivatives, further confirm the chiral nature of the order. These findings demonstrate that the large BCQ directly arises from the chiral loop-current order, establishing a microscopic link between the breaking of time-reversal symmetry and the observed third-order nonlinear Hall response.

Conclusion:

In summary, when the applied current exceeds the critical current, the device transitions from a superconducting state to a finite-resistance state due to vortex dynamics, but **the underlying CDW order remains intact**. The nonlinear Hall signals, which depend on the chiral flux phase, continue to be observed, confirming that the high-current regime is still within the CDW phase, not a trivial or normal metallic state.

The Fig. R2 have been added to the main text as Fig. 4.

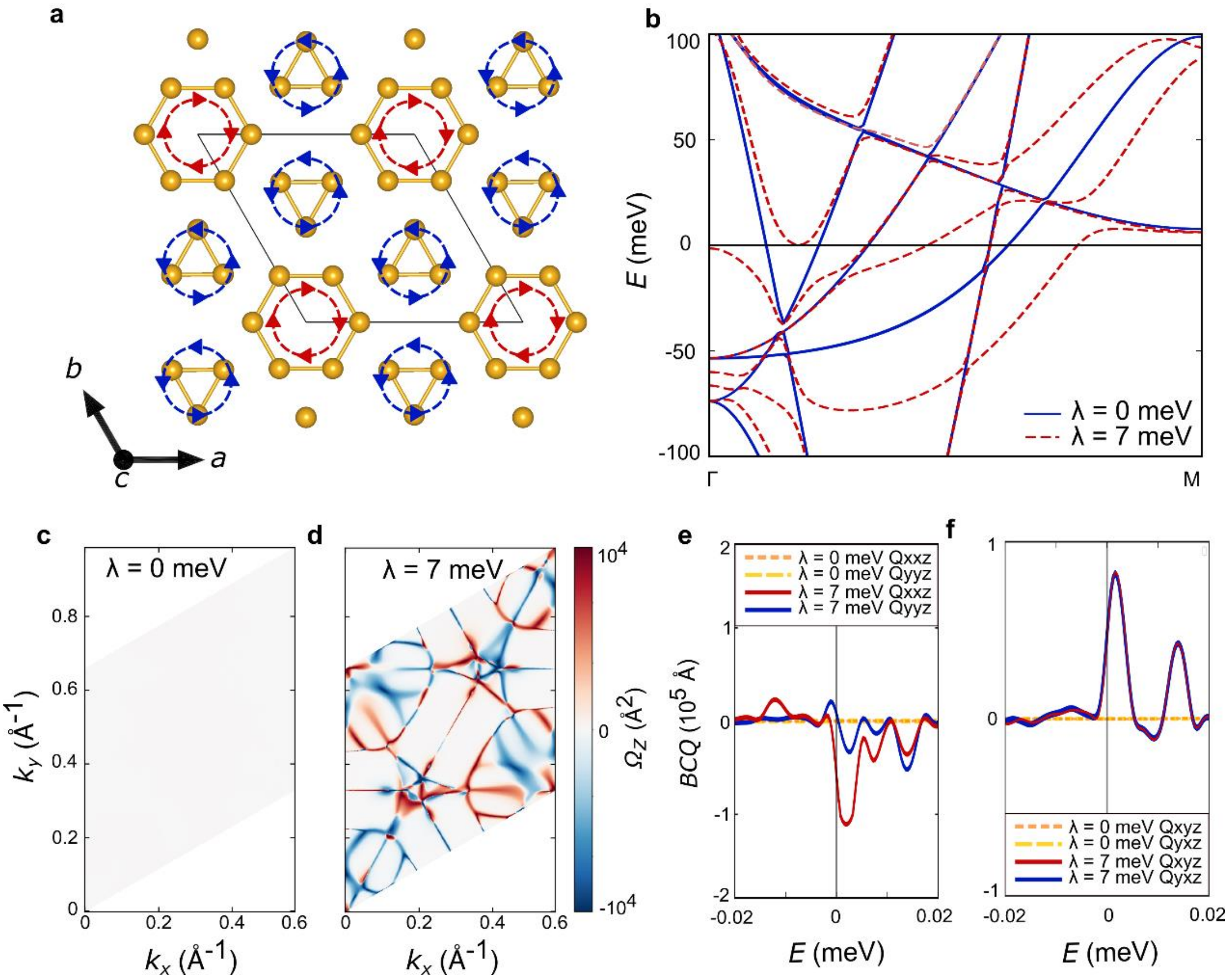

Figure R2. BCQ induced by chiral loop-current order. (a) Schematic illustration of the complex CDW phase in CsV_3Sb_5. Three V sublattices of Kagome network are shown in blue, red and green with the 3Q lattice distortions and the associated chiral loop currents. (b) Electronic band structure along the Γ-M direction calculated without (λ= 0 meV, black solid lines) and with (λ= 7 meV, red dashed lines) loop-current order. The loop-current order reconstructs the low-energy electronic structure and lifts the time reversal band degeneracies near Fermi energy. Here, the λ denotes the loop current strength. (c, d) Berry curvature distribution of CsV_3Sb_5 in the CDW phase without (λ= 0 meV) and with loop currents (λ= 7 meV). Finite Berry curvature emerges only in the presence of loop-current order, indicating time-reversal-symmetry breaking. (e, f) Fermi energy dependence of diagonal and off-diagonal components of Berry curvature quadrupole (BCQ) in the absence (λ= 0 meV, dashed line) and presence of loop currents (λ= 7 meV, solid lines) loop-current order. The introduction of loop currents dramatically enhances both diagonal and off-diagonal BCQ components, generating pronounced peaks in the vicinity of the calculated Fermi energy, whereas the BCQ remains nearly vanished in the absence of loop-current order.

Comment 3:

Regarding Fig 2 f, g, the authors claim that all transverse voltages exhibit clear quadratic, cubic and higher-order dependence on the applied current. However, this is not clear to me. Could the authors provide the corresponding fitting curves?

Furthermore, please clarify whether this specific nonlinear current dependence holds consistently across both the superconducting (SC) state and the state above the critical current.

Response:

We thank the Reviewer for this helpful suggestion and plots showing quadratic, cubic and high-order dependent relationships are shown below. For the n-th harmonic response, the measured nonlinear transverse voltage is expected to satisfy $V_{xy}^{n\omega} \propto I^n$, where I is the applied excitation current, and $V_{xy}^{n\omega}$ is the n-th harmonic voltage. To verify this relation experimentally, we convert the measured voltages into electric fields using $E_{xy}^{n\omega} = V_{xy}^{n\omega}/w$, and $E_{xx} = V_{xx}/l$, where w and l are the transverse and longitudinal distances, respectively. E_{xx}^{n} denotes the n-th power of E_{xx}. Since the longitudinal electric field scales linearly with current, the expected current dependence can be equivalently expressed as $E_{xy}^{n\omega} \propto E_{xx}^{n}$.

Therefore, we plot the $E_{xy}^{2\omega}$, $E_{xy}^{3\omega}$, $E_{xy}^{4\omega}$, and $E_{xy}^{5\omega}$ as functions of ${E_{xx}}^2$, ${E_{xx}}^3$, ${E_{xx}}^4$, and ${E_{xx}}^5$, respectively. As shown in the Fig. R3, all harmonic signals exhibit approximately linear dependences over a broad temperature range, confirming the expected quadratic, cubic, and higher-order current scaling behaviors.

Importantly, this power-law scaling is observed not only above T_c (>4.2 K), but also in the superconducting state (<4.2 K). For temperatures below T_c, the linear relationship remains valid both in the low-current superconducting regime (shown in Fig. R4) and in the higher-current regime beyond the characteristic current associated with the nonlinear peak structure (Fig. R3). Therefore, the emergence of the peak near the characteristic current does not alter the fundamental current-order dependence of the harmonic responses. Although the sixth-harmonic response is clearly observable, its relatively small amplitude results in larger fitting uncertainty, therefore, we limit the quantitative power-law analysis to the second- through fifth-harmonic signals.

In addition, we performed the measurements using two opposite measurement configurations (reversing the current and voltage terminal orientations simultaneously). The odd-order nonlinear responses retain the same sign and magnitude, whereas the even-order responses reverse sign while preserving nearly the same magnitude (Figs. R3-4), fully consistent with the expected parity characteristics of nonlinear transverse transport. These results further confirm the intrinsic origin and excellent reproducibility of the observed scaling behavior.

The Fig. R3 is shown in Fig. 2a-d in the main text and Supplementary Fig. 2 in the supplementary information. Fig. R4 have been added to Supplementary information as Supplementary Fig. 3.

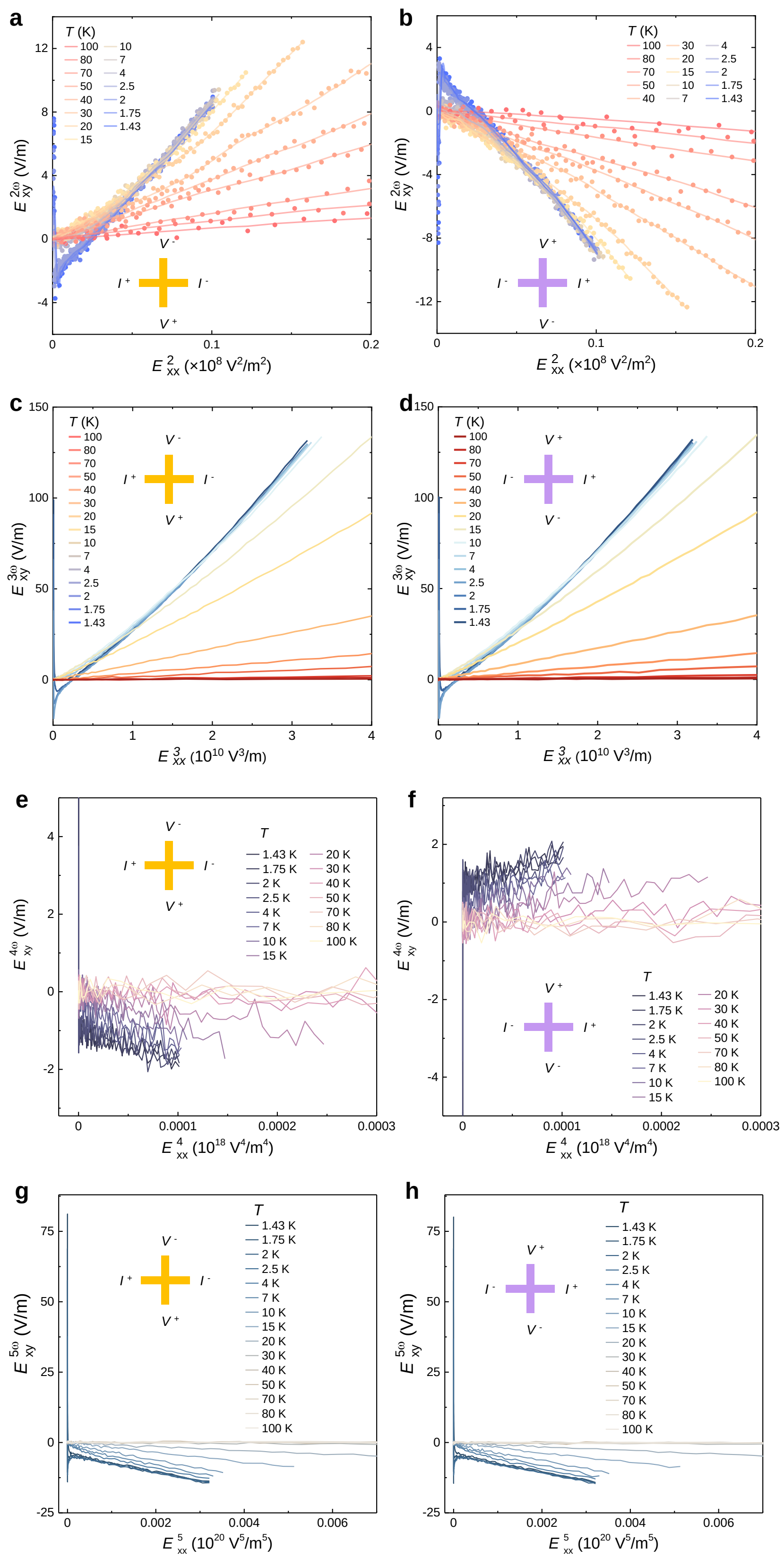

Figure R3. (a-h) $E_{xy}^{2\omega}$, $E_{xy}^{3\omega}$, $E_{xy}^{4\omega}$, and $E_{xy}^{5\omega}$ as functions of E_{xx}^{2}, E_{xx}^{3}, E_{xx}^{4}, and E_{xx}^{5}, respectively, for two opposite measurement configurations at different temperatures. The orange and purple cross structures indicate the multiple-harmonic

measurement setup for the two opposite measurement configurations that reversing the current direction and voltage probe simultaneously.

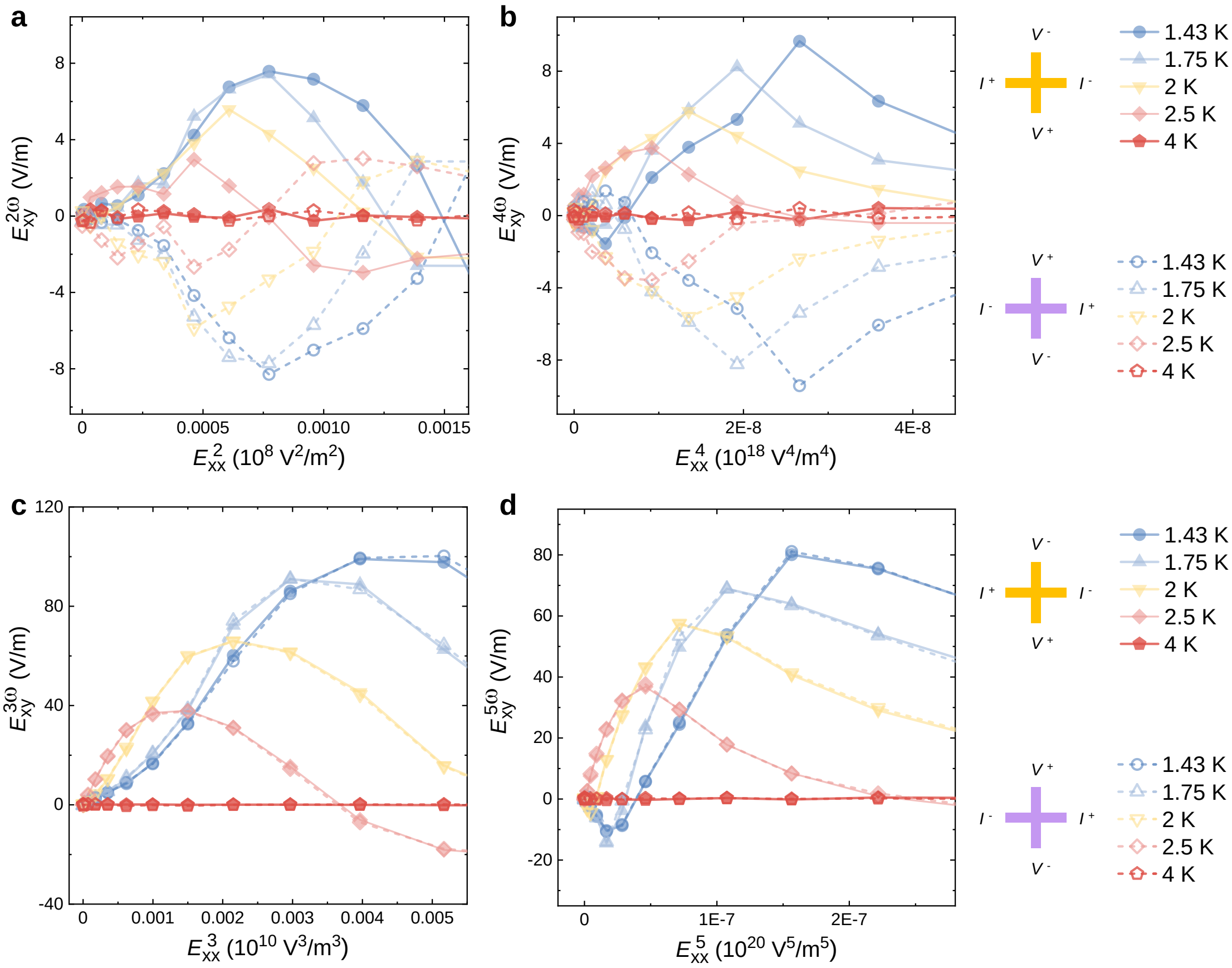

Figure R4. (a-d) $E_{xy}^{2\omega}$, $E_{xy}^{3\omega}$, $E_{xy}^{4\omega}$, and $E_{xy}^{5\omega}$ as functions of E_{xx}^2, E_{xx}^3, E_{xx}^4, and E_{xx}^5 measured in the superconducting state (T<T_c) and below critical current. Solid and dashed lines correspond to the two opposite measurement configurations illustrated in the right panels.

Comment 4:

When applying the out-of-plane magnetic field, is it possible the anti-symmetric part is caused by Berry connection polarizability dipole or called quantum metric dipole?

Response:

We thank the reviewer for raising this important question regarding the nonlinear transport mechanisms. Having established the emergence of finite Berry curvature and Berry curvature quadrupole (BCQ) from loop-current order, we further calculated the and Berry connection polarizability (BCP), an intrinsic band geometric quantity that represents the susceptibility of the Berry connection to an external electric field, also contributes to the third-order non-linear susceptibility[10]. The BCQ describes the second momentum-derivative of the Berry curvature over the equilibrium Fermi distribution and is defined by the rank 3 tensor,

$$Q_{\alpha\beta\gamma} = \int_{\boldsymbol{k}} f_0 \partial_\alpha \partial_\beta \Omega_\gamma.$$

The third order conductivity tensor arising from BCQ scales as,

$$\sigma_{\mu\alpha\beta\gamma}(3\omega) \propto \frac{e^4\tau^2}{4\hbar^3} \epsilon_{\mu\alpha\delta} Q_{\beta\gamma\delta}.$$

Conversely, the BCP contribution arises from the interband coherence and the quantum metric of the Bloch states, captured by the tensor,

$$G_{ab} = 2\mathrm{Re} \sum_{m \neq n} \frac{(A_a)_{nm}(A_b)_{mn}}{\epsilon_n - \epsilon_m},$$

where m, n are band indices and a, b are cartesian indices. $A_{nn} = \langle u_n | \nabla | u_n \rangle$ is the berry connection and u_n is the eigen state corresponding to energy eigenstate ϵ_n. The resulting third-order susceptibility χ^I_{abcd} involves both Fermi Sea terms (derivatives of G_{ab}) and Fermi surface terms (involving the derivative of the distribution function), as given by[11],

$$\chi^I_{abcd} = \tau \int d\boldsymbol{k} (-\partial_a \partial_b G_{cd} + \partial_a \partial_d G_{bc} - \partial_b \partial_d G_{ac}) f_0 + \frac{\tau}{2} \int d\boldsymbol{k}\, v_a v_b G_{cd} f_0''.$$

Unlike BCQ which scales quadratically with τ^2, the governing equation for the BCP clearly shows an overall linear pre-factor of relaxation time, dictating a τ^1 scaling. This difference in scattering time dependence provides the primary theoretical and experimental basis for distinguishing between the field-induced modifications of the Berry connection (BCP) and the intrinsic higher-order multipoles of the Berry curvature (BCQ) in our experiments. Furthermore, the BCP unlike BCQ is not restricted to time reversal symmetry breaking, and therefore remains finite even in the absence of loop-current order. Figure R5a-b presents the calculated BCP tensor components without and with loop-current order, respectively. While the loop currents enhance the overall magnitude of the BCP response, finite BCP contributions already exist in the time-reversal-symmetric state.

Taken together, the calculations reveal a clear distinction between BCQ and BCP. While both quantities contribute to third-order nonlinear transport, only BCQ exhibits a strong sensitivity to loop-current-induced time-reversal-symmetry breaking and follows a quadratic conductivity scaling, while BCP follows a linear scaling.

Combing the theoretical analysis with our experiment results, we can rule the BCP out as the primary cause of the anti-symmetric peak for two reasons:

1. Temperature dependence**:** The BCP dipole is an intrinsic property of the CDW electronic structure. Since the CDW phase and its associated loop currents persist up to ~94 K, a BCP-driven signal would be expected to survive well into the normal state. However, our experimental data in Fig. 5 in the main text (redrawn in Fig. R6) shows that the anti-symmetric component vanishes abruptly above the superconducting transition temperature (~4.2 K). This strict correlation with the superconducting phase boundary identifies vortex dynamics as the driving mechanism.
2. Scaling Behavior: Displayed in Fig. R7, our transport analysis shows that the third-order nonlinear Hall response scales with the square of the conductivity ($\sigma_{xx}{}^2$). This scaling is a hallmark of the Berry curvature quadrupole (BCQ)

contribution[12]. In contrast, the BCP contribution typically scales linearly[11]. The observed scaling further supports the BCQ mechanism amplified by vortex flow over a pure BCP dipole effect.

These observations identify BCQ as the dominant intrinsic mechanism underlying the third-order nonlinear Hall response in the CDW phase of CsV_3Sb_5.

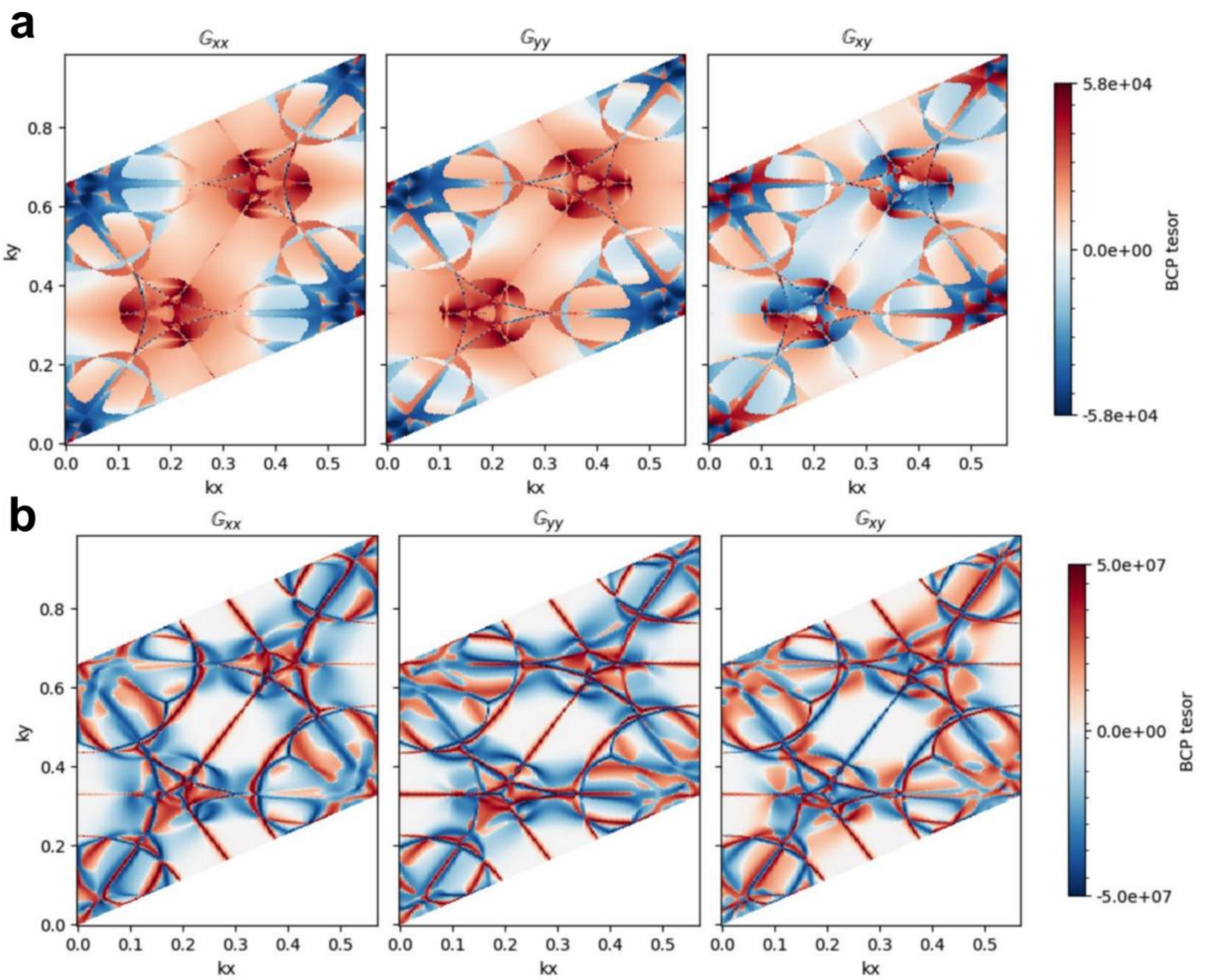

Figure R5. Berry connection polarizability (BCP) in CsV_3Sb_5. (a) BCP tensor components ($\mathbb{G}_{ij}$) in the absence of loop currents ($\lambda = 0$ meV). (b) BCP tensor components in the presence of the loop-current order ($\lambda = 7$ meV). Unlike the BCQ, the BCP remains finite even without time-reversal-symmetry breaking, although its magnitude is enhanced by the loop-current order.

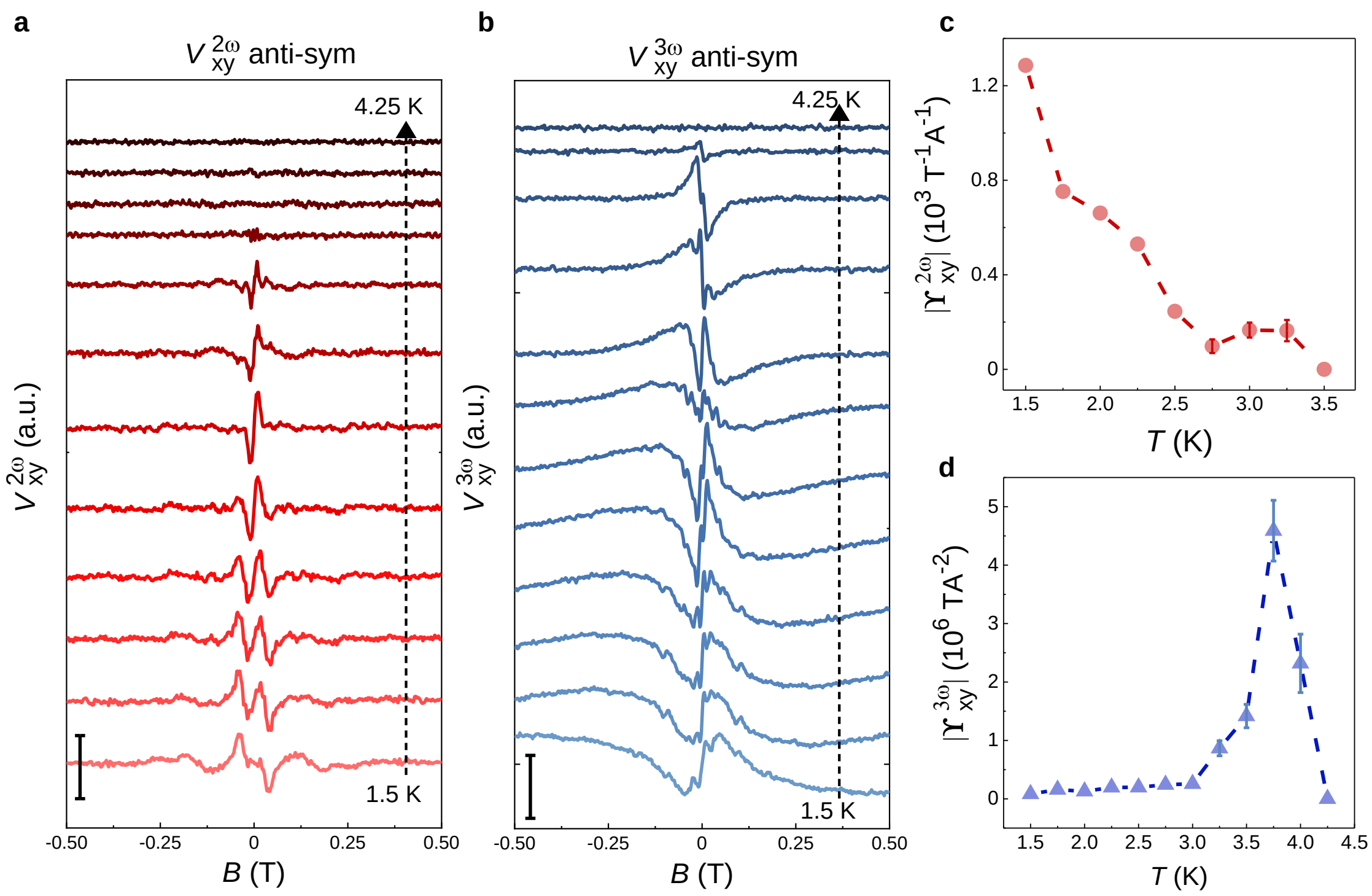

Figure R6. Nonreciprocal transverse transport in CsV_3Sb_5, driven by vortex motion. (a, b) Anti-symmetric symmetric part of $V_{xy}^{2\omega}$ and $V_{xy}^{3\omega}$ evolution with respect of out-of-plane magnetic field. Scale bar: 1 μV. (c, d) Extracted $\gamma_{xy}^{2\omega}$ and $\gamma_{xy}^{3\omega}$ as a function of temperature. The anti-symmetric peak feature of both $V_{xy}^{2\omega}$ and $V_{xy}^{3\omega}$ vanishes above the superconducting transition temperature of 4 K.

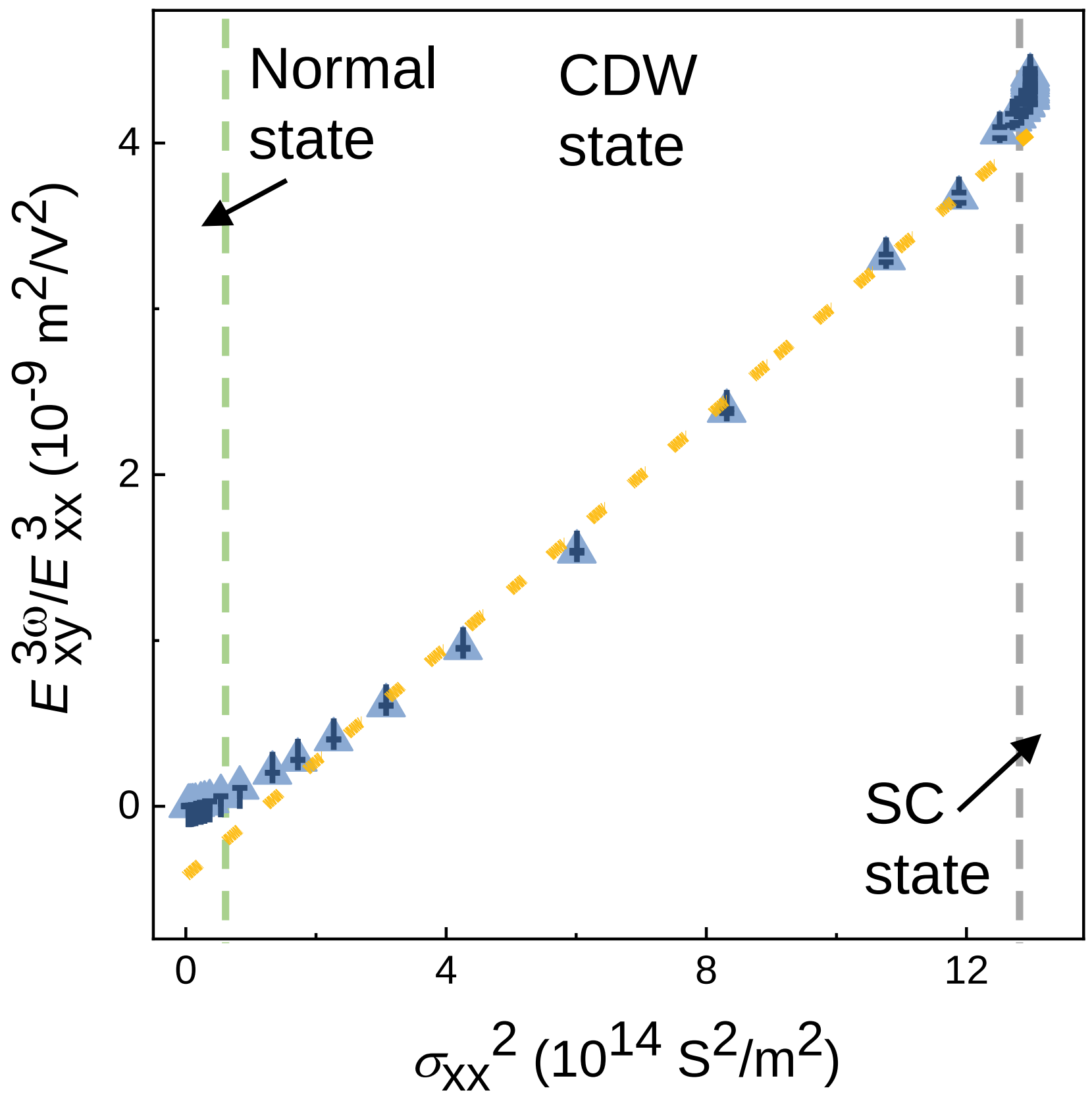

Figure R7. Nonlinear susceptibility $\frac{E_{xy}^{3\omega}}{E_{xx}{}^{3}}$ as a function of the square of conductivity ($\sigma_{xx}{}^{2}$).

Comment 5:

In Fig S1a, why does Vxy not show superconducting responses at low current? For comparison, in Fig S4a, Vxx shows superconducting responses when the current is below around 0.6 mA.

Response:

We thank the Reviewer for this careful comment. The difference between Fig. S1a and Fig. S4a may arises from device-to-device variation and the different device geometries. V_{xy} in Fig. S1a was measured on a Hall-bar device, whereas V_{xx} in Fig. S4a was obtained from a circular-disk device. In this Hall-bar device, a finite residual transverse voltage is not necessarily unexpected, since vortex dynamics can generate a transverse response through the vortex Hall effect. In addition, the device's local inhomogeneity, edge disorder, nonuniform current distribution may further contribute to the residual background signal Therefore, the raw V_{xy} in Fig. S1a does not show a fully zero-voltage response at low current.

In contrast, the circular-disk device exhibits a more ideal superconducting response, with the longitudinal voltage becoming nearly zero below the characteristic current. Importantly, the nonlinear Hall peak is observed in both devices, including the circular-disk device where the low-current voltage is essentially zero. This

demonstrates that the nonlinear Hall peak is not caused by the residual transverse voltage in the Hall-bar device, but is a robust superconductivity-related nonlinear response.

Comment 6:

Minor point: In Fig S8, (d, e) Anti-symmetric and symmetric part of …. This appears to be a typo and should be corrected to (b, d).

Response:

We thank the Reviewer for the careful reading of our manuscript, and we have revised (d, e) to (b, d).

Response to Reviewer #2

General comment:

The manuscript reports the experimental measurements of high-order nonlinear Hall effect up to the sixth harmonic in Kagome superconductor CsV3Sb5. The vortex dynamics near the superconducting transition of CsV3Sb5 are probed through third-order nonlinear Hall effect with assistance of Berry curvature quadrupoles effect. This work paves a way to explore subtle quantum phase transition using the nonlinear transport techniques, which could be greatly advance the relevant research community. These observations are noteworthy, but the analyses are lacking rigorous. I will recommend its publication only after the following issues have been well addressed.

Response:

We sincerely thank the Reviewer for the careful reading of our manuscript and for the positive and insightful comments on our work. We are particularly grateful for the Reviewer's insightful comments regarding the exploration of subtle quantum phase transition by the nonlinear transport techniques. To improve the analyses, we have performed Please see below for the detailed, point-by-point responses to each of the technical comments.

Comment 1:

In Figure 2, the authors attribute the enhancement of nonlinear transverse voltages near 70 K to the formation of charge density wave (CDW) order. While the second-harmonic response exhibits a significant enhancement at temperature closes to the CDW transition, the third-harmonic response shows a pronounced enhancement at temperatures substantially below the CDW transition. I recommend the authors explain the effect of CDW order on the nonlinear response in different harmonics and provide more experimental evidence or theoretical analysis to substantiate this interpretation.

Response:

We thank the reviewer for pointing out this distinct temperature evolution, which highlights the different symmetry-breaking origins of the even and odd harmonics in our exfoliated Kagome lattice device. In the pristine state, CsV_3Sb_5 preserves both

inversion symmetry (IS) and time-reversal symmetry (TRS). It has been shown that the system in its CDW phase, forms a 2×2×2 or 2×2×4 sized unit cell that preserves the bulk IS[13–15], but the emergence of chiral loop currents breaks TRS[16,17].

Because the bulk CDW structure remains centrosymmetric, an intrinsic bulk second-harmonic response is symmetry-forbidden[18,19]. The observed second-harmonic response may originate from the electronic surface states with broken inversion-symmetry[20]. This mechanism becomes particularly relevant in our ~10 nm thick devices, where the enhanced surface-to-volume ratio amplifies the nonlinear signals. The pronounced enhancement of the second-harmonic signal below the CDW transition suggests that the CDW-induced reconstruction of the surface structure further strengthens the nonlinear response. In particular, the emergence of rotational-symmetry breaking (nematicity), chiral charge order, and enhanced electronic anisotropy in the CDW phase may substantially amplify the inversion-breaking nonlinear transport response.

In contrast, the third-harmonic response is driven by the bulk Berry curvature quadrupole (BCQ), which requires broken TRS[12]. To gain microscopic insight into this, we performed first-principles calculations for the complex CDW phase of CsV_3Sb_5. It has been shown to be a 3Q ordered phase[8,9] with the real part of the order parameters (rCDW) leading to either trihexagonal or star of David type atomic distortions within the V Kagome lattice and the imaginary part of the order parameter (iCDW) is associated with the chiral flux generated by time reversal symmetry breaking loop currents. Starting from the experimentally determined CDW supercell structure reported by X-ray diffraction studies[8], we constructed a Wannier-based tight-binding model incorporated loop-current order through the nearest neighbour hopping interactions between V atoms as $t_{ij} \rightarrow t_{ij} + \lambda e^{i\phi_{ij}}$ where λ denotes the loop-current strength and ϕ is the directional phase assigned to each specific bond to dictate the flow of the loop currents. Having incorporated the static structural modulations via first-principles calculations, and to isolate the effect of the purely imaginary hopping channel associated with the chiral flux phase, we set $\phi_{ij} = \pm\pi/2$, corresponding to a state with maximum time-reversal symmetry breaking while maintaining a minimal parameter space. Furthermore, in accordance with CDW gap opening found in previous experiments, the parameter λ has been chosen to be 7 meV for calculations of chiral flux phase. A schematic illustration of the 3Q CDW ordered state with chiral flux phase is shown in Fig. R8a. The calculated electronic band structures along the high symmetric $\Gamma - M$ direction are displayed in Fig. R8b. Compared with $\lambda = 0$ meV case (without loop-current), the introduction of loop-current order ($\lambda = 7$ meV) reconstructs the low-energy electronic structure, breaks the time reversal symmetry and lifts the band degeneracies near the Fermi level. In Fig. R8b the band structure is shown in black solid lines for $\lambda = 0$ meV and red dashed line for $\lambda = 7$ meV, showing the breaking of degeneracy in the presence of the loop currents. More importantly, finite out-of-plane Berry curvature component (Ω_z) emerges only in the presence of loop-current order, whereas it remains symmetry forbidden for $\lambda = 0$ meV, as shown in Fig. R8c-d.

Having identified the microscopic origin of the Berry curvature in the CDW phase of CsV_3Sb_5 to the imaginary part of the complex CDW order, we next evaluate the band-geometric quantities relevant to nonlinear Hall transport. The BCQ, a rank 3 tensor for the 2D Kagome plane, has four finite components, namely Q_{xxz}, Q_{yyz}, Q_{xyz} and Q_{yxz}. The calculated Fermi-energy dependence of these tensor elements is shown in Fig. R8e-f. In the absence of loop-current order ($\lambda = 0$ meV), all BCQ components remain nearly vanished throughout the investigated energy window. In sharp contrast, introducing loop currents ($\lambda = 7$ meV) generates pronounced BCQ peaks in the vicinity of the calculated Fermi energy and enhances both diagonal (Q_{xxz} and Q_{yyz}) and off-diagonal (Q_{xyz} and Q_{yxz}) components by several orders of magnitude. The different sign and magnitude of the diagonal components indicate the broken nematicity phase whereas the off-diagonal components (Q_{xyz} and Q_{yxz}) are indistinguishable as the commutability of partial derivatives dictates. These results demonstrate that the giant BCQ emerges directly from the chiral loop-current order and establish a microscopic connection between time-reversal-symmetry breaking and the observed third-order nonlinear Hall response.

Berry connection polarizability (BCP), an intrinsic band geometric quantity that. The BCQ describes the second momentum-derivative of the Berry curvature over the equilibrium Fermi distribution and is defined by the rank 3 tensor,

$$Q_{\alpha\beta\gamma} = \int_{\boldsymbol{k}} f_0 \partial_\alpha \partial_\beta \Omega_\gamma .$$

The third order conductivity tensor arising from BCQ scales as,

$$\sigma_{\mu\alpha\beta\gamma}(3\omega) \propto \frac{e^4\tau^2}{4\hbar^3} \epsilon_{\mu\alpha\delta} Q_{\beta\gamma\delta} .$$

Conversely, the BCP represents the susceptibility of the Berry connection to an external electric field, also contributes to the third-order non-linear susceptibility[10] contributed from the interband coherence and the quantum metric of the Bloch states, captured by the tensor,

$$G_{ab} = 2\mathrm{Re} \sum_{m\neq n} \frac{(A_a)_{nm}(A_b)_{mn}}{\epsilon_n - \epsilon_m},$$

where m, n are band indices and a, b are cartesian indices. $A_{nn} = \langle u_n|\nabla|u_n\rangle$ is the berry connection and u_n is the eigen state corresponding to energy eigenstate ϵ_n. The resulting third-order susceptibility χ^I_{abcd} involves both Fermi Sea terms (derivatives of G_{ab}) and Fermi surface terms (involving the derivative of the distribution function), as given by[11],

$$\chi^I_{abcd} = \tau \int d\boldsymbol{k}(-\partial_a\partial_b G_{cd} + \partial_a\partial_d G_{bc} - \partial_b\partial_d G_{ac}) f_0 + \frac{\tau}{2}\int d\boldsymbol{k}\, v_a v_b G_{cd} f_0'' .$$

Unlike BCQ which scales quadratically with τ^2, the governing equation for the BCP clearly shows an overall linear pre-factor of relaxation time, dictating a τ^1 scaling. This difference in scattering time dependence provides the primary theoretical and experimental basis for distinguishing between the field-induced modifications of the Berry connection (BCP) and the intrinsic higher-order multipoles of the Berry curvature (BCQ) in our experiments. Furthermore, the BCP unlike BCQ is not restricted to time reversal symmetry breaking, and therefore remains finite even in the absence of

loop-current order. Figure R9a and 9b presents the calculated BCP tensor components without and with loop-current order, respectively. While the loop currents enhance the overall magnitude of the BCP response, finite BCP contributions already exist in the time-reversal-symmetric state.

The chiral loop currents in the CDW state provide this exact symmetry breaking This intrinsic BCQ mechanism is strictly confirmed by our observation that the third-order Hall response scales with the square of the conductivity (σ_{xx} ^{2})[12], rather than the linear scaling with σ_{xx} expected from Berry connection polarizability (BCP)[11]. The pronounced enhancement of the third-harmonic response at temperatures substantially below 50 K reflects the temperature evolution of the loop currents. As suggested by muon spin relaxation (μSR) studies, these time-reversal symmetry-breaking loop currents become fully coherent at lower temperatures[21,22], leading to the massive enhancement of the BCQ-driven third-harmonic signal well below the initial structural CDW onset.

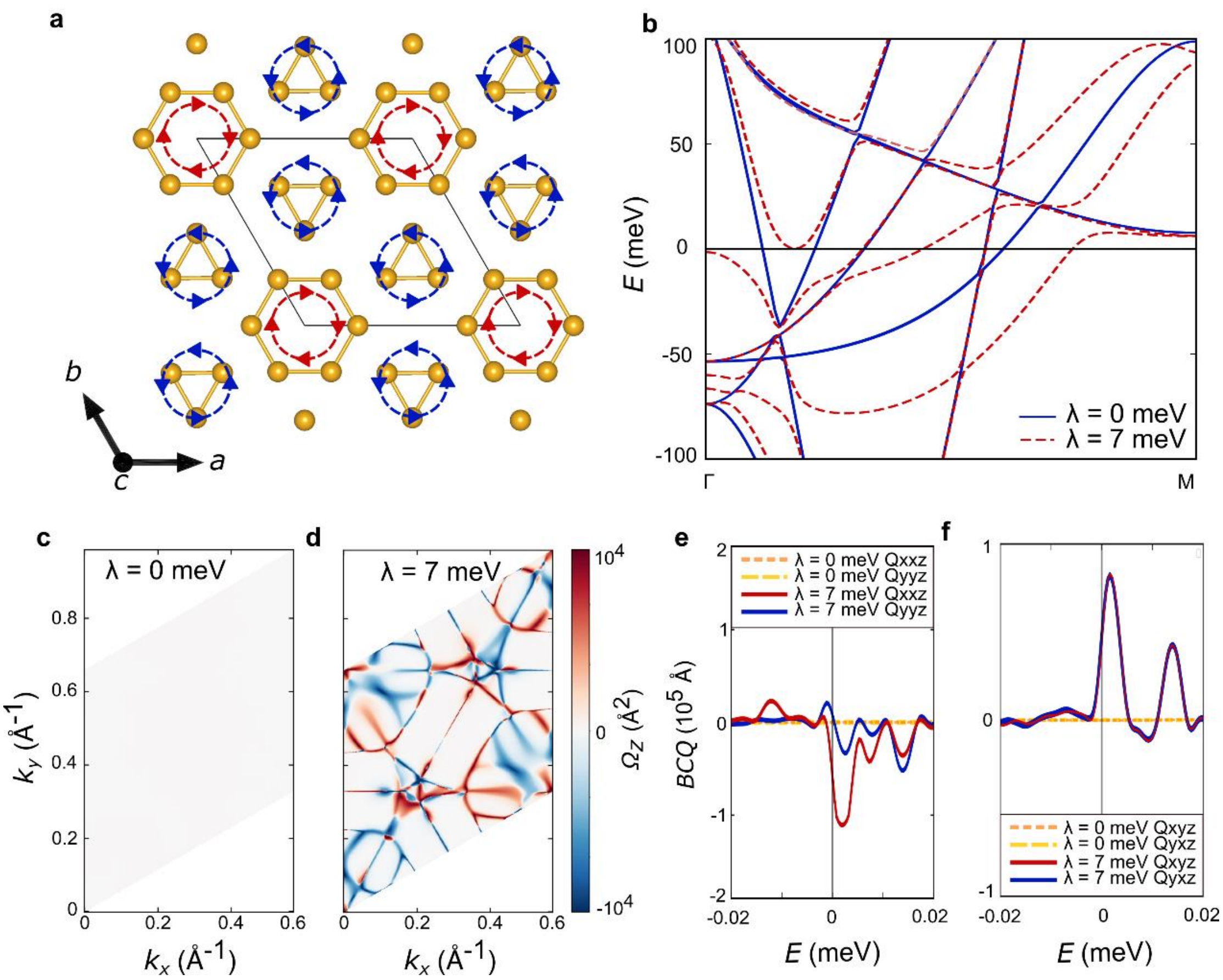

Figure R8. BCQ induced by chiral loop-current order. (a) Schematic illustration of the complex CDW phase in CsV_3Sb_5. Three V sublattices of Kagome network are shown in blue, red and green with the 3Q lattice distortions and the associated chiral loop currents. (b) Electronic band structure along the Γ-M direction calculated without (λ= 0 meV, black solid lines) and with (λ= 7 meV, red dashed lines) loop-current order. The loop-current order reconstructs the low-energy electronic structure and lifts the time reversal band degeneracies near Fermi energy. Here, the λ denotes the loop

current strength. (c, d) Berry curvature distribution of CsV_3Sb_5 in the CDW phase without (λ= 0 meV) and with loop currents (λ= 7 meV). Finite Berry curvature emerges only in the presence of loop-current order, indicating time-reversal-symmetry breaking. (e, f) Fermi energy dependence of diagonal and off-diagonal components of Berry curvature quadrupole (BCQ) in the absence (λ= 0 meV, dashed line) and presence of loop currents (λ= 7 meV, solid lines) loop-current order. The introduction of loop currents dramatically enhances both diagonal and off-diagonal BCQ components, generating pronounced peaks in the vicinity of the calculated Fermi energy, whereas the BCQ remains nearly vanished in the absence of loop-current order.

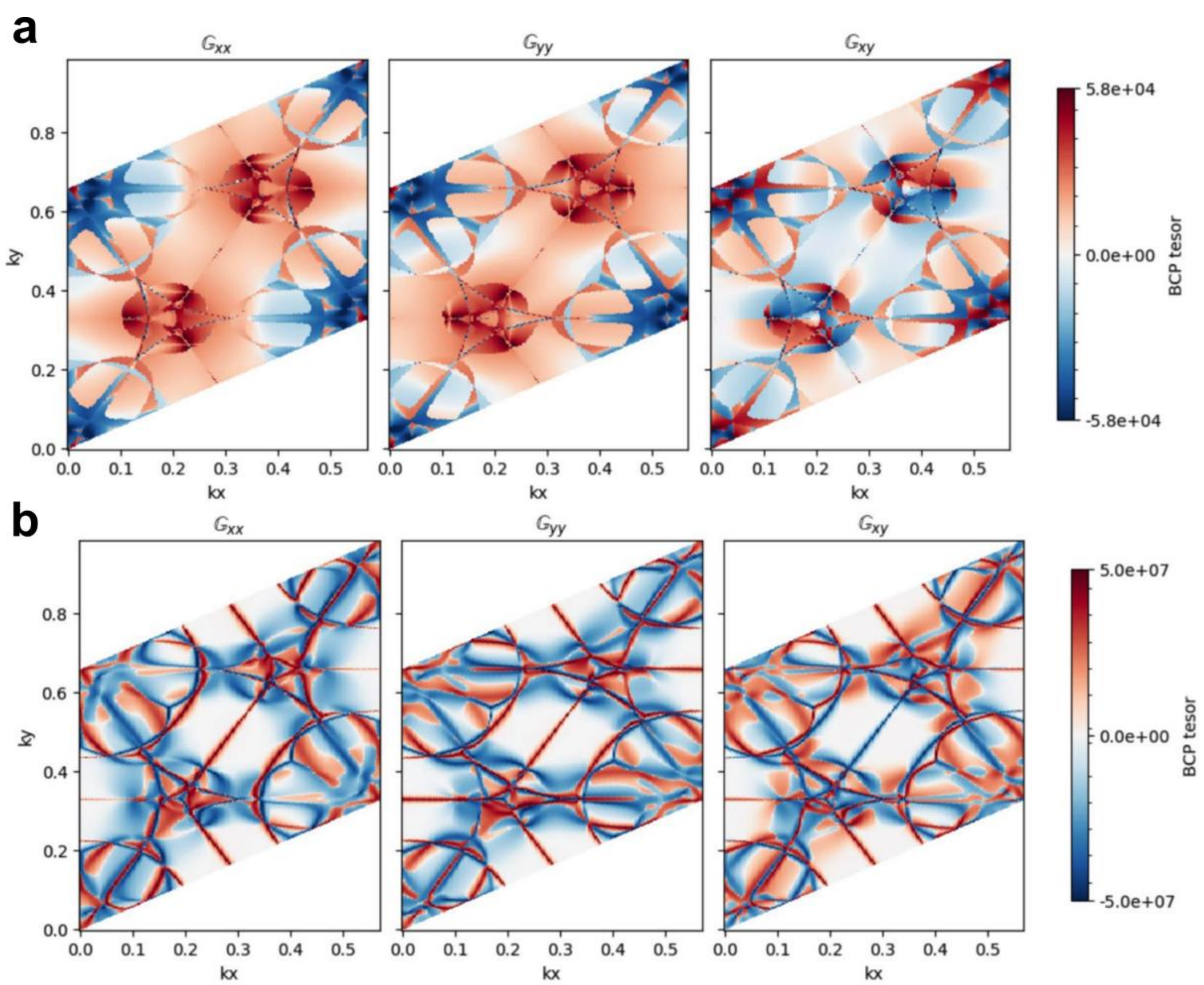

Figure R9. Berry connection polarizability (BCP) in CsV_3Sb_5. (a) BCP tensor components ($\mathbb{G}_{ij}$) in the absence of loop currents ($\lambda = 0$ meV). (b) BCP tensor components in the presence of the loop-current order ($\lambda = 7$ meV). Unlike the BCQ, the BCP remains finite even without time-reversal-symmetry breaking, although its magnitude is enhanced by the loop-current order.

Comment 2:

Figure 2b appears to present two sets of second-harmonic Hall responses with opposite sign reversals. Could the author clarify the origin of these results and identify the mechanism responsible for the sign reversal?

Response:

We thank the reviewer for this important question. The two second-harmonic Hall traces with opposite signs in Figure 2b originate from reversing the current direction

together with the transverse voltage definition. In our measurement configuration, the transverse voltage is always defined relative to the current-flow direction, i.e., the voltage probe on the "right-hand side" of the current direction is consistently taken as the positive terminal. Therefore, when the current direction is reversed, the transverse voltage-detection axis is simultaneously reversed in the laboratory frame. As a result, although the second-order nonlinear Hall response scales quadratically with current ($V_{xy}^{2\omega} \propto I^2$), the measured transverse voltage changes sign because the transverse projection direction is also reversed (Fig. R10). The sign reversal therefore does not originate from the quadratic current dependence itself, but from the reversal of the transverse voltage definition associated with the current direction.

Importantly, the two traces in Fig. 2b (redrawn in Fig. R10) do not correspond to two distinct nonlinear Hall states or mechanisms, but rather represent the same transverse nonlinear response measured under opposite current/probe configurations. The observed sign reversal therefore originates from the reversal of the transverse voltage definition associated with the current direction, instead of a reversal of the intrinsic second-order response itself.

This convention follows the standard protocol widely used in second-order nonlinear Hall measurements. For example, in few-layer WTe_2, Kang *et al.* reported that the second-harmonic Hall voltage reverses sign under simultaneous reversal of current direction and transverse voltage definition, which is regarded as a hallmark of a genuine transverse nonlinear Hall response rather than longitudinal-voltage mixing[23]. Similar current-reversal tests have also been broadly adopted in nonlinear Hall measurements to distinguish intrinsic transverse nonlinear signals from trivial contact asymmetry, Joule-heating-induced voltages, or thermoelectric artifacts[24–26].

Physically, the second-order nonlinear Hall response can be written as[27,28]:

$$j_i^{(2)} = \chi_{ijk} E_j E_k,$$

where χ_{ijk} is the second-order nonlinear conductivity tensor allowed by crystal symmetry. Unlike the linear Hall conductivity constrained by Onsager reciprocity, the second-order nonlinear Hall response arises from a higher-order nonequilibrium transport tensor and therefore can remain finite under zero magnetic field. In this case, the generated transverse electric field is even under algebraic current reversal due to the quadratic dependence on E. However, the experimentally detected voltage corresponds to the projection of the transverse field onto the chosen probe direction. Consequently, exchanging the transverse voltage leads introduces an additional minus sign in the measured voltage.

We have clarified this point in the revised manuscript. The observed sign reversal under simultaneous current/probe reversal, together with the quadratic current scaling and transverse measurement geometry, further supports the nonlinear Hall origin of the signal and helps exclude trivial longitudinal-voltage pickup.

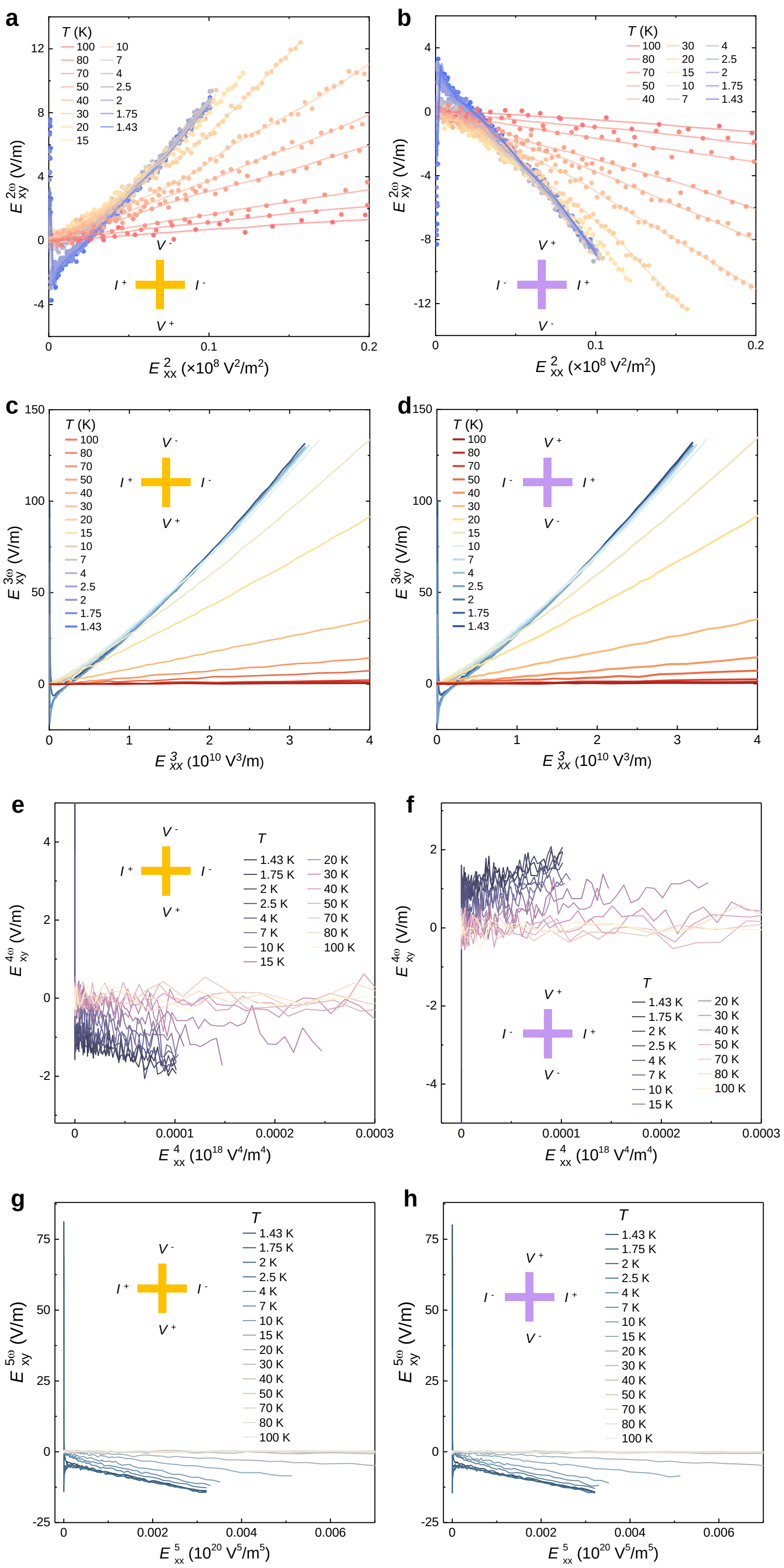

Figure R10. (a-h) $E_{xy}^{2\omega}$, $E_{xy}^{3\omega}$, $E_{xy}^{4\omega}$, and $E_{xy}^{5\omega}$ as functions of ${E_{xx}}^2$, ${E_{xx}}^3$, ${E_{xx}}^4$, and ${E_{xx}}^5$, respectively, for two opposite measurement configurations at different temperatures. The orange and purple cross structures indicate the multiple-harmonic measurement setup for the two

opposite measurement configurations that reversing the current direction and voltage probe simultaneously.

Comment 3:

The emergence of the peak in the I-V measurements at relatively small a.c. current may be influenced by the lock-in amplifier, for instance, changes in sensitivity range or input dynamics. In addition, the peak position can also be varied as the series resistance in the measurement circuit changes. The correlation between the peak evolution with the superconducting transition lacks robust experimental and theoretical support. The authors should provide control experiments or analyses to rule out measurement artifacts and to firmly establish the relationship with superconductivity.

Response:

We thank the Reviewer for this important comment. We agree that instrumental artifacts must be carefully excluded before attributing the observed peak structure to superconductivity.

First, the excitation current was supplied by a Keithley 6221 current source. The 6221 possesses a very high output impedance (MΩ-GΩ), which is orders of magnitude larger than the resistance of the superconducting device (Ω-kΩ). Consequently, the actual current flowing through the sample is determined by the current source and remains essentially unchanged by variations in the sample resistance or moderate changes in external series resistance. Therefore, the observed peak cannot be fully attributed to changes in the excitation current caused by variations in circuit resistance.

Second, during the measurements, the lock-in sensitivity range was fixed at 50 mV throughout the entire current sweep. Therefore, the peak feature does not originate from sensitivity-range switching.

To further address the Reviewer's concern regarding lock-in amplifier dynamics, we performed a series of control measurements by systematically varying the lock-in settings. Specifically, we changed:

(i) the input dynamic mode (High Reserve, Normal, and Low Noise);

(ii) the sensitivity range (10 mV, 20 mV, 50 mV, 100 mV, 200 mV, and 500 mV);

(iii) the time constant (30 ms, 100 ms, 300 ms, 1 s, 3 s, and 10 s);

(iv) the filter slope (12, 18, and 24 dB/oct).

As shown in Figs. R11-12, No systematic changes in the peak position or overall signal evolution are observed upon varying the lock-in settings. Only the shortest time constant (30 ms) produces larger fluctuations due to reduced signal averaging, while the peak structure itself remains unchanged.

Furthermore, the same peak behavior was independently observed using two different lock-in amplifiers (SR830 and OE1022D), demonstrating that the effect is not specific to a particular instrument.

Most importantly, the peak appears only in the superconducting state and disappears completely above T_C ~4. 2 K (Fig. 2a, 2f and 2g, and redrawn in Fig. R13). In addition, the peak evolves systematically with the superconducting transition and is reproducibly observed in multiple devices and measurement

configurations. Such behavior is difficult to reconcile with instrumental artifacts arising from lock-in sensitivity settings, reserve modes, input dynamics, filtering parameters, or circuit resistance.

Therefore, these control experiments strongly support that the observed peak is an intrinsic superconductivity-related nonlinear response rather than a measurement artifact.

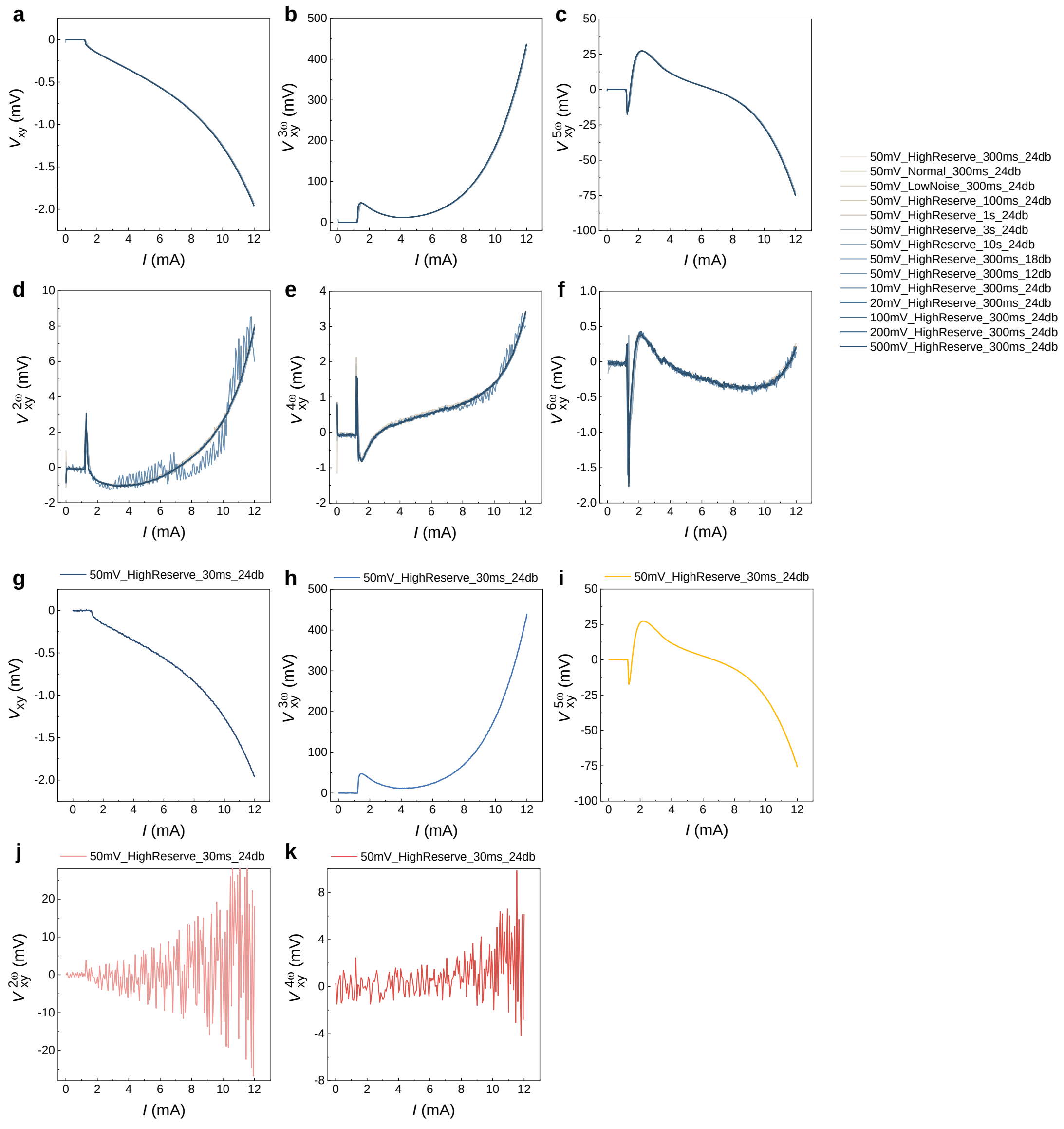

Figure R11. Dependence of the nonlinear Hall responses of channel 1-2 on lock-in amplifier settings. (a–f) V_{xy}, $V_{xy}^{2\omega}$, $V_{xy}^{3\omega}$, $V_{xy}^{4\omega}$, $V_{xy}^{5\omega}$ and $V_{xy}^{6\omega}$ measured under different reserve modes (High Reserve, Normal, and Low Noise), sensitivity ranges (10–500 mV), time constants (30 ms–10 s), and filter slopes (12–24 dB/oct). (g–k) V_{xy}, $V_{xy}^{2\omega}$, $V_{xy}^{3\omega}$, $V_{xy}^{4\omega}$ and $V_{xy}^{5\omega}$ measured at time constants of 30 ms. No systematic changes in the peak position or overall signal evolution are observed upon varying the lock-in settings.

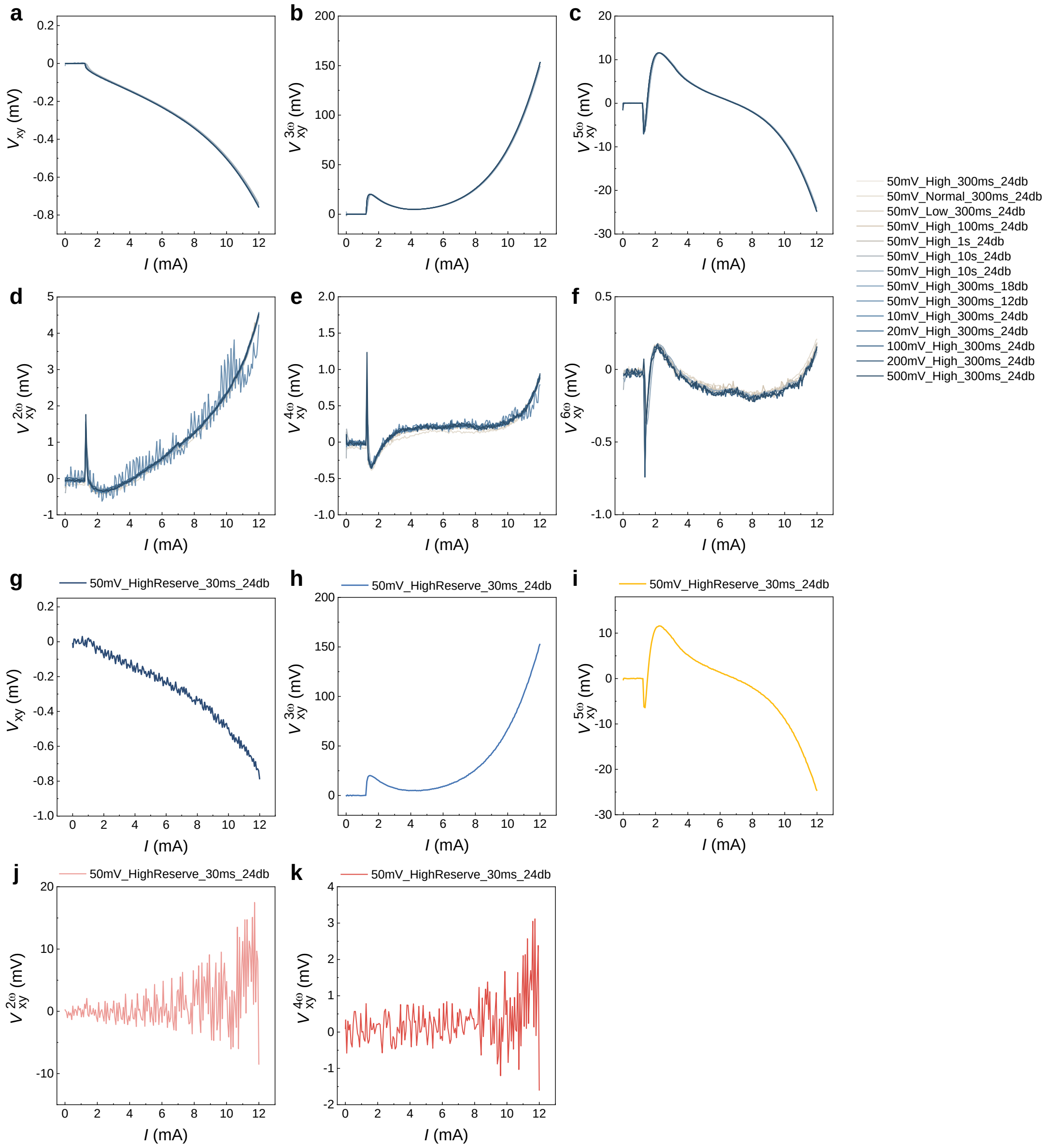

Figure R12. Dependence of the nonlinear Hall responses of channel 3-4 on lock-in amplifier settings. (a–f) V_{xy}, $V_{xy}^{2\omega}$, $V_{xy}^{3\omega}$, $V_{xy}^{4\omega}$, $V_{xy}^{5\omega}$ and $V_{xy}^{6\omega}$ measured under different reserve modes (High Reserve, Normal, and Low Noise), sensitivity ranges (10–500 mV), time constants (30 ms–10 s), and filter slopes (12–24 dB/oct). (g–k) V_{xy}, $V_{xy}^{2\omega}$, $V_{xy}^{3\omega}$, $V_{xy}^{4\omega}$ and $V_{xy}^{5\omega}$ measured at time constants of 30 ms. No systematic changes in the peak position or overall signal evolution are observed upon varying the lock-in settings.

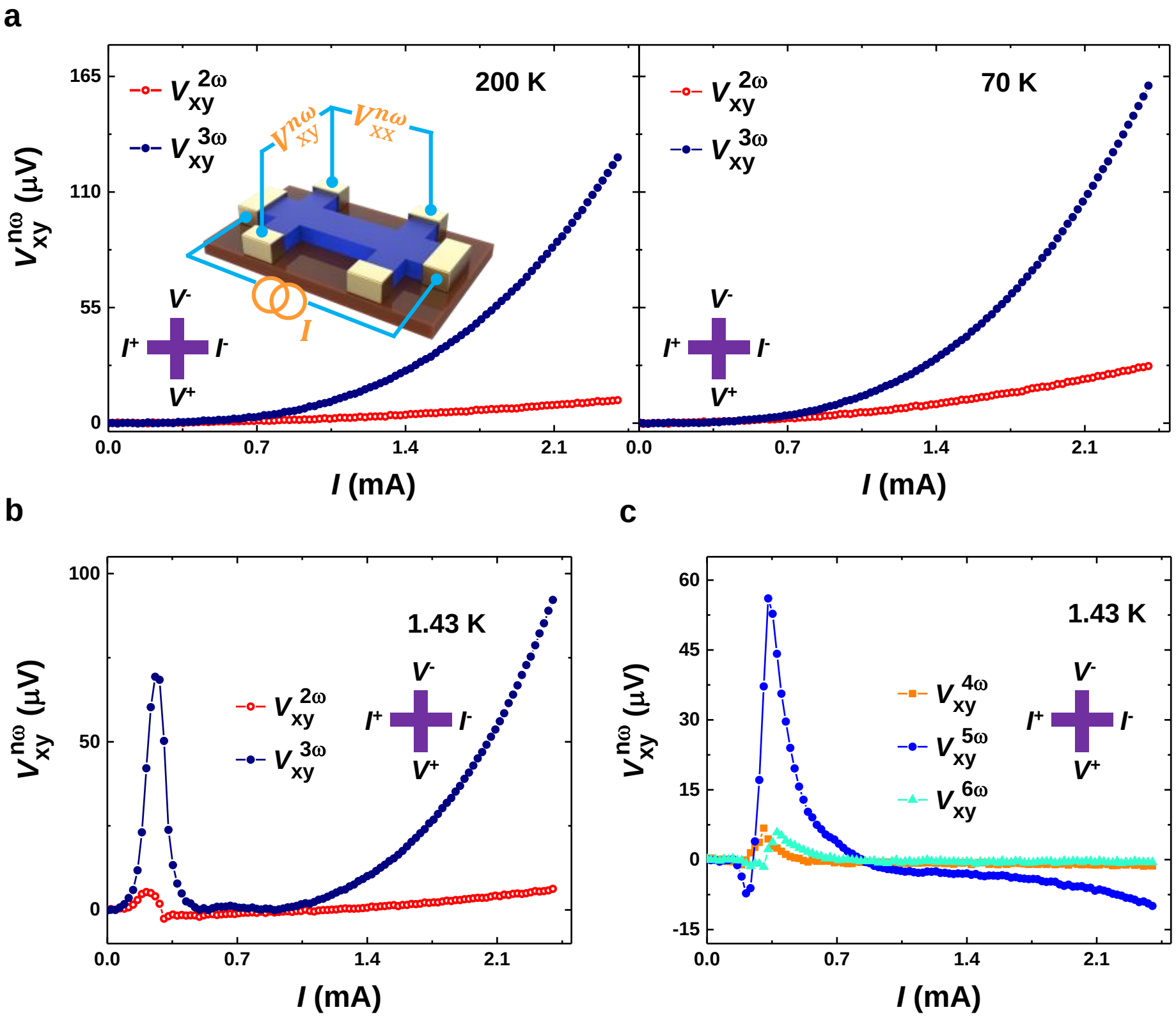

Figure 13. Comparison of nonlinear Hall response in CsV_3Sb_5 above and below T_c. (a) The second- and third-harmonic transverse voltages measured above and below the CDW transition, at 200 K and 70 K, respectively. Left inset, schematic structure of the multiple-harmonic measurement setup. (b, c) Multiple-order harmonic transverse voltage as a function of alternating current measured at 1.43 K. In the superconducting state, pronounced peaks emerged in the second- to sixth-harmonic transverse voltages ($V_{xy}^{2\omega}$, $V_{xy}^{3\omega}$, $V_{xy}^{4\omega}$, $V_{xy}^{5\omega}$, and $V_{xy}^{6\omega}$) near the superconducting transition.

Comment 4:

As shown in Figure S1a, the superconducting state is suppressed as excitation current exceeds ~0.5 mA. However, the nonlinear responses at different harmonics are obtained in both superconducting and normal states. Could the author identify the distinct physical origins of the nonlinear responses in these two phases?

Response:

We thank the reviewer for this insightful question, which touches on the core message of our manuscript. We can indeed distinctly separate the physical origins of the nonlinear responses in these two regimes:

1. The Normal State (> ~0.5 mA): When the excitation current suppresses superconductivity, the device is driven into the coexisting normal state, which is the charge density wave (CDW) state. In this regime, the nonlinear responses are intrinsic and originate strictly from the symmetry-breaking geometry of the lattice and its band structure. The second-harmonic response originates from local inversion-symmetry breaking at the surfaces of our ~10 nm thin films. Concurrently, the baseline third-

harmonic response is driven by the intrinsic bulk Berry curvature quadrupole (BCQ), which is enabled by the time-reversal symmetry-breaking chiral loop currents characteristic of the CDW phase.

2. The Superconducting State (< ~0.5 mA): In contrast, the massive enhancement of the nonlinear responses observed below the critical current has a dynamical origin. As the system enters the vortex-flow regime, the motion of vortices induces rapid spatial variations in the superconducting phase gradient. These dynamical phase gradients massively amplify the underlying Berry curvature multipoles (specifically dominating the odd-order BCQ responses), leading to the giant nonlinear signals that peak near the transition before dropping to zero at the true zero-resistance superconducting ground state.

Comment 5:

How the authors exclude the thermal effects from the measured nonlinear responses as the excitation current is as large as 3 mA, such as such as Joule heating or thermoelectric voltages?

Response:

We thank the Reviewer for raising this important point. We apologize for an error in the original manuscript. The current values shown in Fig. 2 were inadvertently reported as peak values, whereas the corresponding RMS values should have been used. We therefore corrected all current values by dividing them by 1.4142 ($\sqrt{2}$) in the revised manuscript. Accordingly, the maximum current in Fig. 2 is approximately 2.4 mA in RMS units. This correction does not affect the experimental results, data analysis, or the conclusions of the manuscript.

To address potential thermal contributions—such as Joule heating or thermoelectric voltages—we carefully analyzed the possible effects and their characteristics.

While we cannot entirely rule out a small symmetric background contribution potentially arising from residual heating or signal mixing, such effects are unlikely to account for the dominant features we observe—namely, the peak structure intimately linked with the superconducting state and the field-antisymmetric nonlinear response.

First, the nonlinear signals we observe are remarkably stable over a broad excitation-frequency range (see Supplementary Fig. 4, and redrawn in Fig. R14). Thermal effects, such as Joule heating, typically depend on thermal relaxation processes with characteristic timescales. As a result, signals arising from thermal effects are expected to diminish at higher excitation frequencies due to the finite thermal response time. The weak frequency dependence of our nonlinear response suggests it is unlikely to be driven by thermal relaxation.

Second, the observed nonlinear transverse voltages exhibit sign reversal upon simultaneous reversal of the excitation current direction and the transverse voltage probes (Fig. R10). This behavior aligns with the expected symmetry of intrinsic nonlinear Hall effects, which are non-reciprocal and depend on the direction of current flow relative to the crystal axes. Moreover, by systematically changing the input current

direction and switching the measurement contacts, we mapped the angular dependence of the nonlinear Hall response (Fig. R15). The resulting anisotropic behavior further supports a genuine nonlinear transport origin, as thermal effects, being isotropic, would generally produce a symmetric, non-direction-dependent response.

Third, the temperature dependence of the signal provides additional evidence (Fig. R10). The peak in the nonlinear response disappears above the critical temperature T_c. If the signal were primarily due to heating, one would expect it to persist or even intensify in the normal state due to increased resistance and Joule dissipation. The fact that the peak correlates strongly with the superconducting state indicates an intrinsic origin rather than a thermal artifact.

Furthermore, we utilize the symmetry properties of the measurement setup, specifically the transverse Hall geometry and magnetic field reversal, to distinguish between different contributions. Onsager reciprocal relations provide a theoretical foundation here: they imply that, under time-reversal symmetry, certain transport coefficients are symmetric or antisymmetric with respect to magnetic field reversal. In our measurements, the nonlinear Hall response that is antisymmetric with respect to magnetic field reversal is consistent with intrinsic effects related to vortex dynamics and nonlinear Hall effects (Fig. 5 in the main text and redrawn in Fig. R16), rather than thermal biases, which would generally produce symmetric responses independent of field direction.

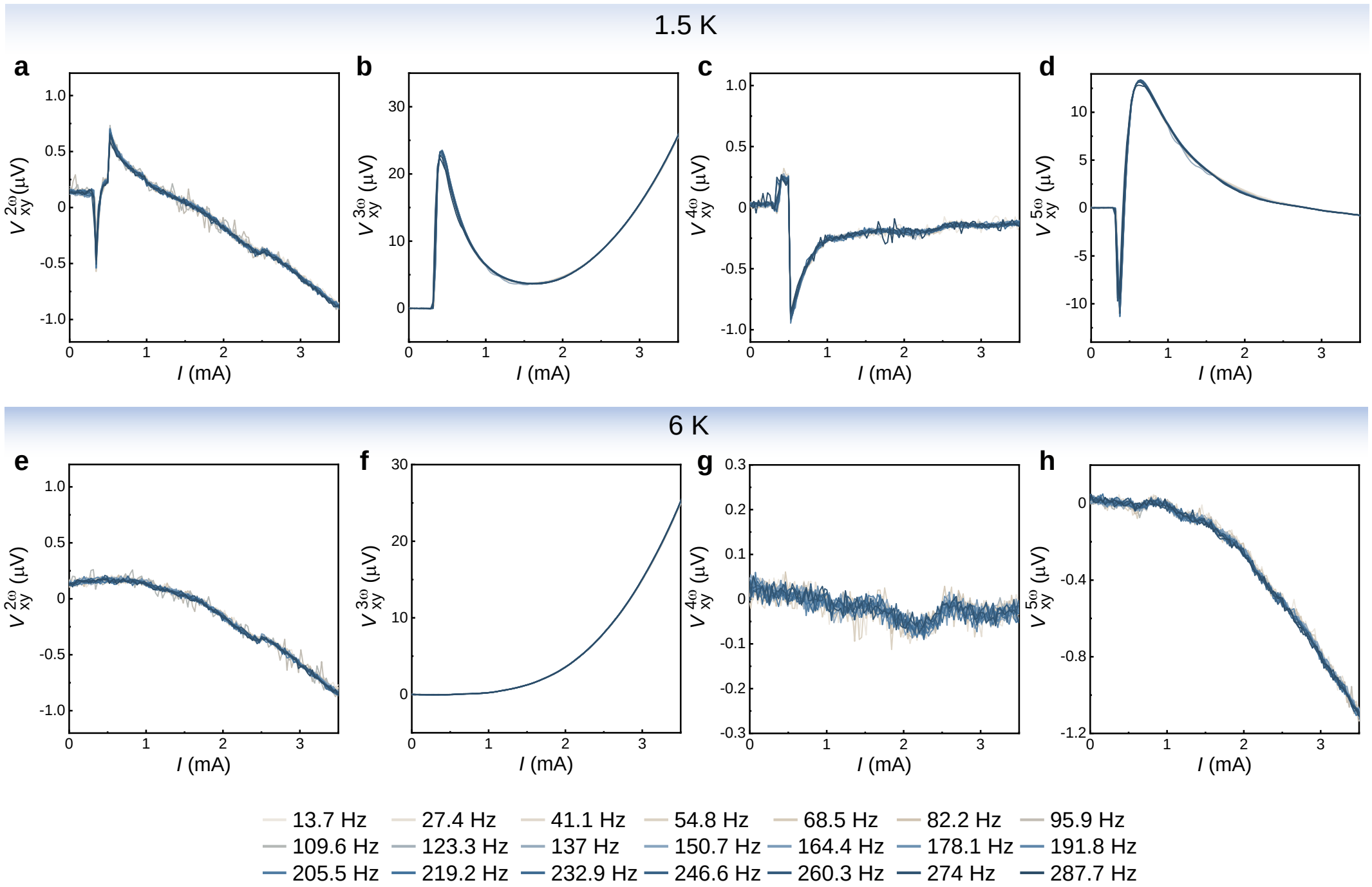

Figure R14. Frequency-dependent multiple-harmonic transverse response as a function of current at 1.5 K (a-d) and 6 K (e-h). The overlapped nonlinear responses also help to exclude the thermal and capacitance coupling effects.

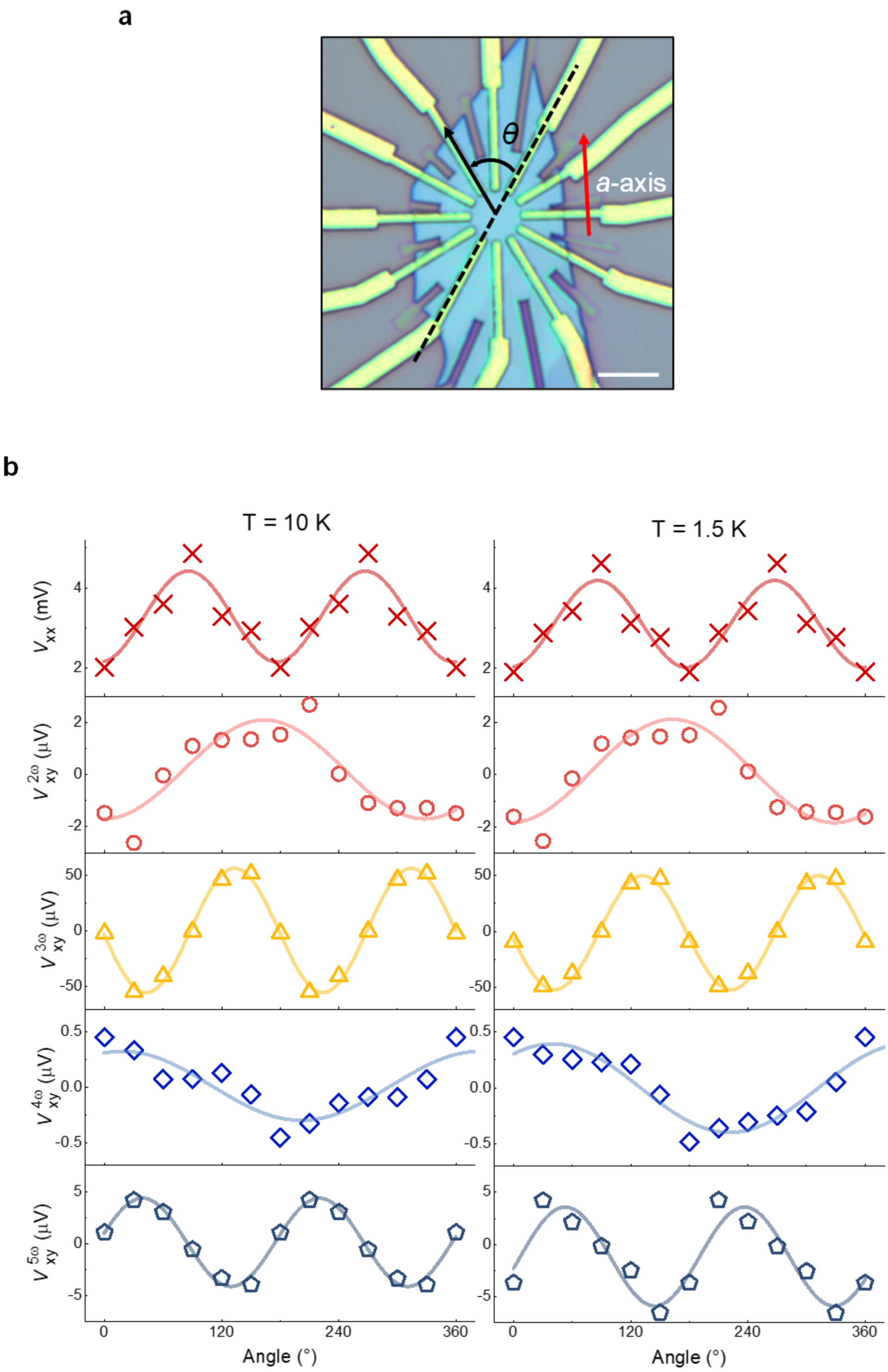

Figure R15. Angular dependence of the nonlinear transport in CsV_3Sb_5 device. (a) Optical image of disk-like device. Scale bar: 5 μm. (b) Angular dependence of the first-order longitudinal resistance (R_{xx}) and second-, third-, fourth-, and fifth-harmonic transverse voltages measured at 1.5 K and 10 K, respectively. The solid lines are fits to sinusoidal functions with one-fold (360° period) and two-fold (180° period) angular symmetries. Odd-order transverse components exhibit a pronounced two-fold angular symmetry, while even-order transverse components show a nearly one-fold response in both the superconducting and CDW states.

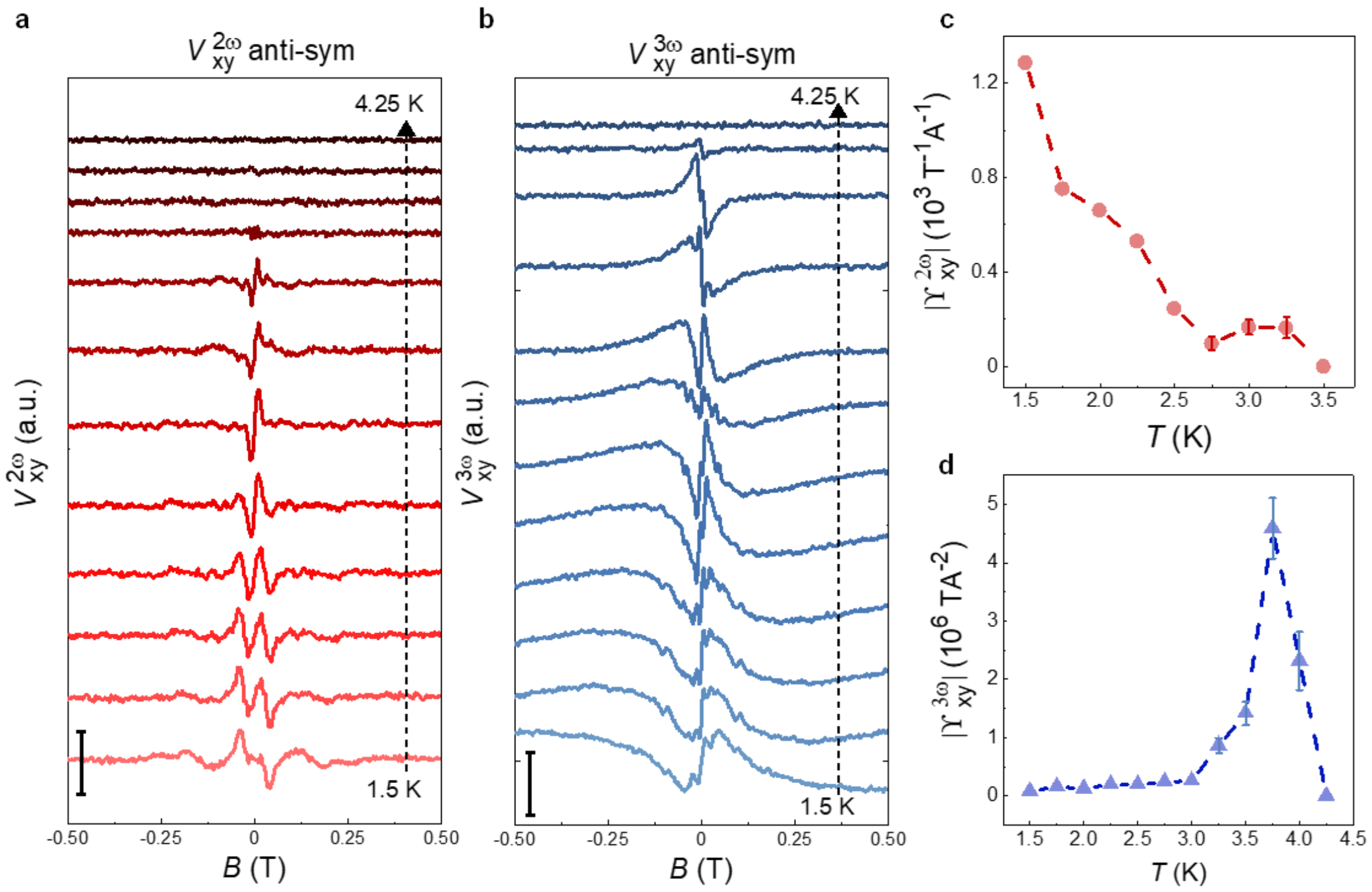

Figure R16. Nonreciprocal transverse transport in CsV_3Sb_5, driven by vortex motion. (a, b) Anti-symmetric symmetric part of $V_{xy}^{2\omega}$ and $V_{xy}^{3\omega}$ evolution with respect of out-of-plane magnetic field. Scale bar: 1 μV. (c, d) Extracted $\gamma_{xy}^{2\omega}$ and $\gamma_{xy}^{3\omega}$ as a function of temperature. The anti-symmetric peak feature of both $V_{xy}^{2\omega}$ and $V_{xy}^{3\omega}$ vanishes above the superconducting transition temperature of 4.2 K.

Response to Reviewer #3

General comment:

I co-reviewed this manuscript with one of the reviewers who provided the listed reports. This is part of the Nature Communications initiative to facilitate training in peer review and to provide appropriate recognition for Early Career Researchers who co-review manuscripts.

Response:

We sincerely thank the Reviewer for the careful reading of our manuscript and for the positive and insightful comments on our work. We appreciate the Reviewer's recognition of the timeliness and the comprehensive nature of our experimental investigations. Please see above for the detailed, point-by-point responses to each of the technical comments.

Reference:

1. Nie, L. *et al.* Charge-density-wave-driven electronic nematicity in a kagome superconductor. *Nature* **604**, 59–64 (2022).
2. Fukushima, K. *et al.* Violation of emergent rotational symmetry in the hexagonal Kagome superconductor CsV3Sb5. *Nat. Commun.* **15**, (2024).
3. Chen, Q., Chen, D., Schnelle, W., Felser, C. & Gaulin, B. D. Charge Density Wave Order and Fluctuations above T_{CDW} and below Superconducting T_C in the Kagome Metal CsV3Sb5. *Phys. Rev. Lett.* **129**, 056401 (2022).
4. Zhao, H. *et al.* Cascade of correlated electron states in the kagome superconductor CsV3Sb5. *Nature* **599**, 216–221 (2021).
5. Wu, Y. *et al.* Nonreciprocal charge transport in topological kagome superconductor CsV3Sb5. *Npj Quantum Mater.* **7**, 105 (2022).
6. Zhang, X. *et al.* Vortex phase diagram of the kagome superconductor CsV3Sb5. *Phys. Rev. B* **109**, 144507 (2024).
7. Ding, P., Lee, C. H., Wu, X. & Thomale, R. Diagnosis of pairing symmetry by vortex and edge spectra in kagome superconductors. *Phys. Rev. B* **105**, 174518 (2022).
8. Kautzsch, L. *et al.* Structural evolution of the kagome superconductors AV3Sb5 (A = K, Rb, and Cs) through charge density wave order. *Phys. Rev. Mater.* **7**, 024806 (2023).
9. Guo, C. *et al.* Correlated order at the tipping point in the kagome metal CsV3Sb5. *Nat. Phys.* **20**, 579–584 (2024).
10. Lai, S. *et al.* Third-order nonlinear Hall effect induced by the Berry-connection polarizability tensor. *Nat. Nanotechnol.* **16**, 869–873 (2021).
11. Liu, H. *et al.* Berry connection polarizability tensor and third-order Hall effect. *Phys. Rev. B* **105**, 045118 (2022).
12. Zhang, C.-P., Gao, X.-J., Xie, Y.-M., Po, H. C. & Law, K. T. Higher-order nonlinear anomalous Hall effects induced by Berry curvature multipoles. *Phys. Rev. B* **107**, 115142 (2023).
13. Liang, Z. *et al.* Three-Dimensional Charge Density Wave and Surface-Dependent Vortex-Core States in a Kagome Superconductor CsV3Sb5. *Phys. Rev. X* **11**, 031026 (2021).
14. Ortiz, B. R. *et al.* Fermi Surface Mapping and the Nature of Charge-Density-Wave Order in the Kagome Superconductor CsV3Sb5. *Phys. Rev. X* **11**, 041030 (2021).
15. Hu, Y. *et al.* Coexistence of trihexagonal and star-of-David pattern in the charge density wave of the kagome superconductor AV3Sb5. *Phys. Rev. B* **106**, L241106 (2022).
16. Feng, X., Jiang, K., Wang, Z. & Hu, J. Chiral flux phase in the Kagome superconductor AV3Sb5. *Sci. Bull.* **66**, 1384–1388 (2021).
17. Christensen, M. H., Birol, T., Andersen, B. M. & Fernandes, R. M. Loop currents in AV3Sb5 kagome metals: Multipolar and toroidal magnetic orders. *Phys. Rev. B* **106**, 144504 (2022).
18. Suárez-Rodríguez, M. *et al.* Nonlinear transport in non-centrosymmetric systems. *Nat. Mater.* **24**, 1005–1018 (2025).

19. Du, Z. Z., Lu, H.-Z. & Xie, X. C. Nonlinear Hall effects. *Nat. Rev. Phys.* **3**, 744–752 (2021).
20. Ortiz, B. R. *et al.* CsV3Sb5 : A Z2 Topological Kagome Metal with a Superconducting Ground State. *Phys. Rev. Lett.* **125**, 247002 (2020).
21. Yu, L. *et al.* Evidence of a hidden flux phase in the topological kagome metal CsV3Sb5. *arXiv.org* https://arxiv.org/abs/2107.10714v1 (2021).
22. Shan, Z. *et al.* Muon spin relaxation study of the layered kagome superconductor CsV3Sb5. *Phys. Rev. Res.* **4**, 033145 (2022).
23. Kang, K., Li, T., Sohn, E., Shan, J. & Mak, K. F. Nonlinear anomalous Hall effect in few-layer WTe_2. *Nat. Mater.* **18**, 324–328 (2019).
24. He, P. *et al.* Quantum frequency doubling in the topological insulator Bi2Se3. *Nat. Commun.* **12**, 698 (2021).
25. Ma, T. *et al.* Growth of bilayer MoTe2 single crystals with strong non-linear Hall effect. *Nat. Commun.* **13**, 5465 (2022).
26. Lu, X. F. *et al.* Nonlinear transport and radio frequency rectification in BiTeBr at room temperature. *Nat. Commun.* **15**, 245 (2024).
27. Sodemann, I. & Fu, L. Quantum Nonlinear Hall Effect Induced by Berry Curvature Dipole in Time-Reversal Invariant Materials. *Phys. Rev. Lett.* **115**, 216806 (2015).
28. Shen, Y. R. *The Principles of Nonlinear Optics*. (Wiley, Hoboken, 2002).

Response to Reviewers' Comments

We thank the Reviewers for their careful reading of our manuscript entitled *"Berry Curvature Multipoles in Kagome Superconductor CsV_3Sb_5"* and for their constructive and insightful comments, which have helped to improve the quality of this work. We are glad that all the reviewers have recommended publication of our work. In response, we have addressed the comment raised by reviewer #2, and detailed responses to each comment are provided below.

Response to Reviewer #1

General comment:

Reviewer #1 (Remarks to the Author):

I have reviewed the revised manuscript and the authors' responses to my previous comments. I am satisfied with the revisions and believe that the authors have addressed the concerns in a reasonable and well-supported manner. The manuscript has been improved substantially, and I support its publication in Nature Communications.

Response:

We sincerely thank the Reviewer for the positive assessment of our revised manuscript and the recommendation for publication of our work.

Response to Reviewer #2

General comment:

The authors have made substantial revisions to the manuscript and addressed all my concerns. I therefore support the publication of the revised manuscript in Nature Communications. The only minor issue is a typo on page 4 of the supplementary information, where "The third-harmonic transverse signal" should be changed to "The fourth-harmonic transverse signal".

Response:

We sincerely thank the Reviewer for providing positive and insightful comments on our manuscript and recommending the publication of our work. We have revised the "The third-harmonic transverse signal" to "The fourth-harmonic transverse signal" in the Supplementary Information.

Response to Reviewer #3

General comment:

I co-reviewed this manuscript with one of the reviewers who provided the listed reports. This is part of the Nature Communications initiative to facilitate training in peer review and to provide appropriate recognition for Early Career Researchers who co-review

manuscripts.

Response:

We sincerely thank the Reviewer for generously investing their time in thoroughly evaluating our work. We appreciate the Reviewer's recognition of the timeliness and the comprehensive nature of our investigations.
